# Association of Short-term Change in Leukocyte Telomere Length With Cortical Thickness and Outcomes of Mental Training Among Healthy Adults: A Randomized Clinical Trial

**DOI:** 10.1001/jamanetworkopen.2019.9687

**Published:** 2019-09-25

**Authors:** Lara M. C. Puhlmann, Sofie L. Valk, Veronika Engert, Boris C. Bernhardt, Jue Lin, Elissa S. Epel, Pascal Vrtička, Tania Singer

**Affiliations:** 1 Research Group, “Social Stress and Family Health,” Max Planck Institute for Human Cognitive and Brain Sciences, Leipzig, Germany; 2 Institute of Systems Neuroscience, Medical Faculty, Heinrich Heine University Düsseldorf, Düsseldorf, Germany; 3 Institute of Neuroscience and Medicine (INM-7: Brain & Behaviour), Research Centre Jülich, Jülich, Germany; 4 Montreal Neurological Institute, McGill University, Montreal, Québec, Canada; 5 Department of Biochemistry and Biophysics, University of California, San Francisco; 6 Department of Psychiatry, University of California, San Francisco; 7 Social Neuroscience Lab, Max Planck Society, Berlin, Germany

## Abstract

**Importance:**

Telomere length is associated with the development of age-related diseases and structural differences in multiple brain regions. It remains unclear, however, whether change in telomere length is linked to brain structure change, and to what extent telomere length can be influenced through mental training.

**Objectives:**

To assess the dynamic associations between leukocyte telomere length (LTL) and cortical thickness (CT), and to determine whether LTL is affected by a longitudinal contemplative mental training intervention.

**Design, Setting, and Participants:**

An open-label efficacy trial of three 3-month mental training modules with healthy, meditation-naive adults was conducted. Data on LTL and CT were collected 4 times over 9 months between April 22, 2013, and March 31, 2015, as part of the ReSource Project. Data analysis was performed between September 23, 2016, and June 21, 2019. Of 1582 eligible individuals, 943 declined to participate; 362 were randomly selected for participation and assigned to training or retest control cohorts, with demographic characteristics matched. The retest control cohorts underwent all testing but no training. Intention-to-treat analysis was performed.

**Interventions:**

Training cohort participants completed 3 modules cultivating interoception and attention (Presence), compassion (Affect), or perspective taking (Perspective).

**Main Outcomes and Measures:**

Change in LTL and CT.

**Results:**

Of the 362 individuals randomized, 30 participants dropped out before study initiation (initial sample, 332). Data were available for analysis of the training intervention in 298 participants (n = 222 training; n = 76 retest control) (175 women [58.7%]; mean [SD] age, 40.5 [9.3] years). The training modules had no effect on LTL. In 699 observations from all 298 participants, mean estimated changes in the relative ratios of telomere repeat copy number to single-copy gene (T/S) were for no training, 0.004 (95% CI, −0.010 to 0.018); Presence, −0.007 (95% CI, −0.025 to 0.011); Affect, −0.005 (95% CI, −0.019 to 0.010); and Perspective, −0.001 (95% CI, −0.017 to 0.016). Cortical thickness change data were analyzed in 167 observations from 67 retest control participants (37 women [55.2%], mean [SD] age, 39.6 [9.0] years). In this retest control cohort subsample, naturally occurring LTL change was related to CT change in the left precuneus extending to the posterior cingulate cortex (mean *t*_161_ = 3.22; *P* < .001; *r* = 0.246). At the individual participant level, leukocyte telomere shortening as well as lengthening were observed. Leukocyte telomere shortening was related to cortical thinning (*t*_77_ = 2.38; *P* = .01; *r* = 0.262), and leukocyte telomere lengthening was related to cortical thickening (*t*_77_ = 2.42; *P* = .009; *r* = 0.266). All analyses controlled for age, sex, and body mass index.

**Conclusions and Relevance:**

The findings of this trial indicate an association between short-term change in LTL and concomitant change in plasticity of the left precuneus extending to the posterior cingulate cortex. This result contributes to the evidence that LTL changes more dynamically on the individual level than previously thought. Further studies are needed to determine potential long-term implications of such change in relation to cellular aging and the development of neurodegenerative disorders. No effect of contemplative mental training was noted in what may be, to date, the longest intervention with healthy adults.

**Trial Registration:**

ClinicalTrials.gov identifier: NCT01833104

## Key Points

**Question:**

Is naturally occurring short-term change in leukocyte telomere length related to structural plasticity of the brain, and can telomere length be influenced through mental training?

**Findings:**

In this randomized clinical trial of 298 healthy adults, the mental training intervention did not affect leukocyte telomere length. Naturally occurring change in leukocyte telomere length over 3 consecutive 3-month intervals was significantly associated with cortical thickness change in the left precuneus extending to the posterior cingulate cortex.

**Meaning:**

This study provides the first evidence to date for an association between short-term change in leukocyte telomere length and brain structure, suggesting that these processes may be mechanistically linked; the mental training used did not influence leukocyte telomere length of healthy, middle-aged adults.

## Teaser

This randomized clinical trial examines the effect of mental attention training on leukocyte telomere length and cortical thickness among healthy adults.

## Introduction

The length of telomeres (ie, protective chromosomal caps) functions as a biomarker for an individual’s health and aging trajectory. Shortened leukocyte telomere length (LTL) has, for example, been associated with greater susceptibility to age-related diseases, including mild cognitive impairment and Alzheimer disease.^1,2,3,4,5,6^ A large-scale, cross-sectional investigation^7^ also identified correlations between shorter LTL and smaller volumes of several brain regions associated with the development of Alzheimer disease, including the hippocampus, amygdala, temporal lobe, precuneus, and posterior cingulate.^8,9^ Cross-sectional evidence therefore suggests that shorter telomeres are associated with neurodegenerative processes. Less is known, however, about how change in telomere length relates to brain structure.

Shorter telomeres have been associated with genetics^10^ and lifestyle factors, such as obesity,^11^ but also with psychological and emotional strains, including early life adversity, chronic stress, rumination, and loneliness.^12,13,14,15,16,17^ Leukocyte telomere length has therefore been described as a psychobiomarker that reflects the combined result of physiological and psychological burdens on an individual’s health and aging profile.^18^ Recent evidence suggests that LTL may change more quickly and dynamically than previously assumed. Leukocyte telomere lengthening, for example, has been observed after 6 months of physical training programs,^19,20^ as well as after an intensive 1-month mental training intervention.^21^ Because telomere lengthening implies a reversal of biological aging processes, these observations have received much attention. However, the biological plausibility of short-term telomere change, herein defined as less than 2 years, remains controversial, particularly for lengthening.^22,23,24^

Relating change in LTL with brain structure may provide insight into the biological implications of short-term LTL change. If short-term LTL change reflects biological processes that are generally meaningful for an individual’s aging trajectory, such change is unlikely to happen in isolation. Rather, LTL change should be associated with changes in other aging- and health-related markers. Structural brain indices are biomarkers of individual differences in aging and health.^25^ The first aim of the present study was therefore to investigate whether naturally occurring aging- or lifestyle-related change in LTL over 9 months was related to structural changes in the brain. Cortical thickness (CT) was selected as our measure of brain structure as a more anatomically specific modality than, for example, volumetric measures,^26,27^ and was accordingly expected to be more sensitive to structural changes, including aging-related gray-matter decline.^27,28^

The second aim of this study was to assess whether training in different mental practices over the same 9-month period could systematically influence LTL, potentially buffering against aging-related shortening or facilitating lengthening. Mental training protocols, such as the mindfulness-based stress reduction program,^29^ have been found to reduce several psychological strains that are associated with shorter telomeres, including rumination, loneliness, and stress.^30,31^ However, of the 9 studies^21,32,33,34,35,36,37,38,39^ that have investigated LTL in association with mindfulness or meditation, as discussed in a review,^40^ only 2 found evidence for a change in LTL. One study observed LTL after a 1-month retreat (N = 28),^21^ and the other after a 5-year lifestyle intervention (N = 10).^32^ The remaining 7 studies used less-intensive or shorter interventions, which may in part explain the absence of LTL change.^33,34,35,36,37,38,39^ Evidence for an effect of mental training on LTL therefore appears preliminary and requires replication on a larger scale, which was possible in the present study.

The present investigation was conducted as part of the ReSource Project,^41^ a longitudinal mental training study that included training cohorts (TCs) and a retest control cohort (RCC). The RCC was used to address our first aim: to assess potential dynamic associations between LTL and CT. We expected associations with CT change in brain regions previously linked cross-sectionally to LTL.^7^ Nonetheless, we conducted analyses on a whole brain level to be able to detect potential aging- or lifestyle-related CT changes more broadly. The main effects of the ReSource Project training on CT have been reported elsewhere.^42^

**Figure 1.**
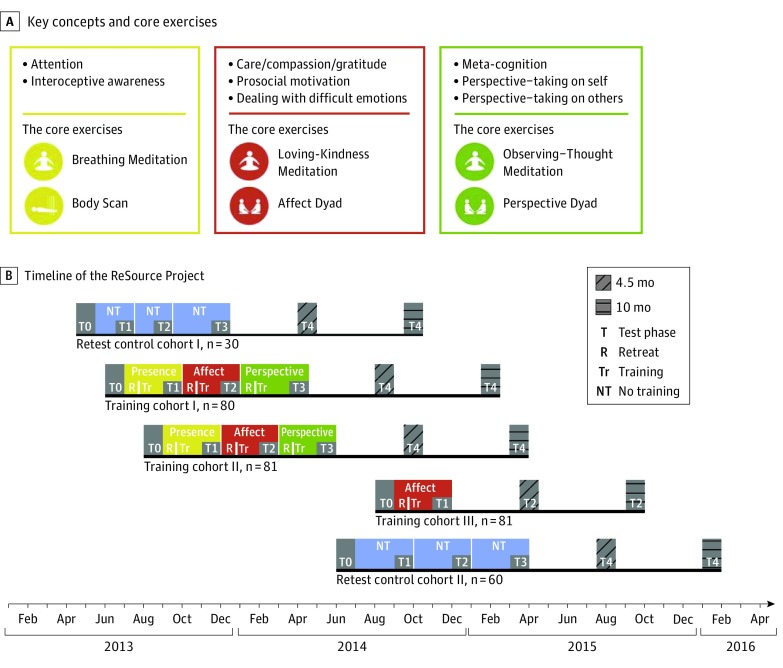
Study Design of the ReSource Project A, Key concepts and core exercises taught during the modules Presence (yellow), Affect (red), and Perspective (green). B, Timeline of the ReSource Project and training sequence per cohort. Retest control participants were recruited in 2 cohorts for logistic reasons but were analyzed jointly. We therefore refer to a single retest control cohort in the text. The displayed study timeline was adapted to most accurately reflect the time points of blood sampling. Test phases for other variables may differ slightly. Samples of retest control cohort I were collected after approximately 2 months of no training before T1 and T2; however, given the smaller sample size of this cohort compared with retest control cohort II, combined RCC sampling occurred approximately every 3 months on average; the same is true for magnetic resonance imaging scans. The full ReSource Project design also included follow-up assessments (T4), but these were not included in the present investigation. Adapted from Singer et al.^43,44^

Our second aim focused on a nonoverlapping sample, namely, participants trained in 3 distinct types of mental practices (TCs). These practices were designed to cultivate attention, interoception, and focus on the present moment (Presence); socioaffective capacities, such as compassion (Affect); and sociocognitive skills, such as meta-cognition (Perspective) (Figure 1).^43,44^ All 3 training modules had the potential to influence LTL by buffering the experience of acute or chronic stress.^31,45^ The Presence module could additionally have influenced LTL by reducing rumination,^46^ whereas the Affect and Perspective modules may have had an effect through reduced loneliness or social isolation.^42,47^ We thus predicted leukocyte telomere lengthening or relative maintenance in the TCs compared with the RCC, which, on average, was expected to show aging-related attrition or no change in LTL. In the case of significant training-induced differences in LTL change, we had planned to subsequently analyze how this systematically induced change relates to structural plasticity of regions associated with LTL in the first aim and exploratively at the whole-brain level.

## Methods

### Participants

**Figure 2.**
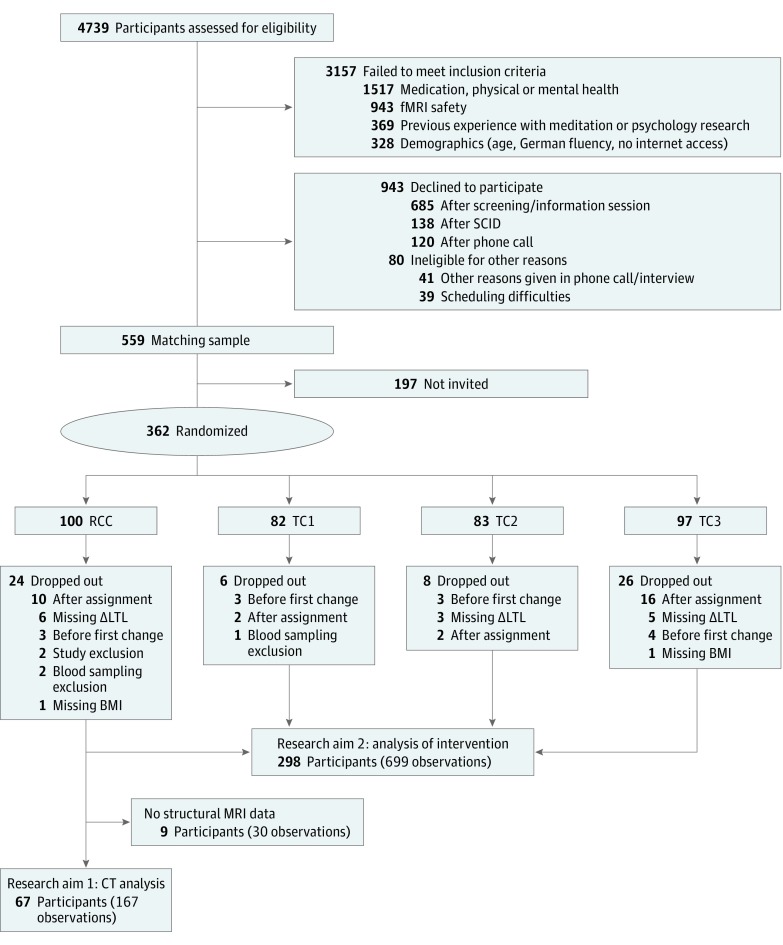
Study Flow Diagram Combined numbers from 2 recruitment periods in 2012-2013 and 2013-2014 are shown. BMI indicates body mass index; CT, cortical thickness; fMRI, functional magnetic resonance imaging; MRI, magnetic resonance imaging; ΔLTL, change in leukocyte telomere length (uncorrected); RCC, retest control cohort; SCID, Structural Clinical Interview for DSM-IV Disorders (Axis I and Axis II)^48,49^; and TC, training cohort. Adapted from Singer et al.^44^

In the context of the ReSource Project,^41^ 362 healthy adults were initially recruited and randomized to retest control or training cohorts (RCC, n=100; TCs, n=262) using bootstrapping without replacement. Following dropout before study commencement, the initial sample consisted of 332 participants (175 women; age, 20-55 years; mean [SD], 40.5 [9.3] years) distributed across the RCC and 3 different training cohorts (RCC, n = 90; TC1, n = 80; TC2, n = 81; TC3, n = 81) (Figure 2).^44,48,49^ The RCC participants underwent all testing but no training, allowing an estimate of retest effects relevant to some measures of the ReSource Project that are not included herein. All participants were meditation-naive and extensively screened for mental and physical illness through questionnaires and 2 clinical diagnostic interviews; further details have been reported elsewhere.^50^ Data for the present investigation were collected between April 22, 2013, and March 31, 2015. Participants gave written informed consent, could withdraw from the study at any time, and were financially compensated. The study was conducted in compliance with the Declaration of Helsinki^51^ and approved by the research ethics committees of the University of Leipzig and the Humboldt University in Berlin, Germany. The trial protocol is available in Supplement 1. This study followed the Consolidated Standards of Reporting Trials (CONSORT) reporting guideline for randomized clinical trials.

### Intervention Design

Data were acquired from participants in the RCC 4 times in intervals of approximately 3 months. The TC participants were tested at similar intervals in which data acquisition followed training in 1 of 3 distinct modules termed Presence, Affect, and Perspective. Training cohorts 1 and 2 practiced the 3 modules in counterbalanced order to account for potential sequence effects. Training cohort 3 completed only the 3-month Affect training to isolate the specific effects of the Presence module from the Affect module (Figure 1B). Further details have been reported elsewhere.^43^ Each module began with a 3-day silent retreat. For the remainder of the training period, participants attended weekly 2-hour group sessions and performed the 2 core practices of each module at home for approximately 30 minutes daily (Figure 1A). The Presence module aimed to cultivate present moment–focused attention and interoception through 2 widely used meditation techniques: Body Scan and Breathing Meditation. The Affect module was designed to cultivate socioaffective qualities, such as compassion, gratitude, and nonjudgmental acceptance of difficult emotions, using Loving-Kindness Meditation and Affect Dyads. The Affect Dyad is a partner exercise specifically aiming at socioaffective abilities.^47^ The Perspective module targeted sociocognitive and metacognitive analytical abilities. The daily core practices of the Perspective module included Observing-Thoughts Meditation and Perspective dyads. As an analog to the Affect Dyad, the Perspective Dyad teaches cognitive perspective taking on the beliefs and thoughts of oneself and others in a partner format. The detailed trial protocol (Supplement 1) has also been published elsewhere.^44^

### Sampling of LTL

Blood was collected at 4 points (T0-T3) and frozen at −80 °C until assay. Genomic DNA was extracted from whole blood (QIAamp DNA blood mini kit; Qiagen). Leukocyte telomere length was measured using quantitative polymerase chain reaction assay as previously described,^52^ and reported as T/S ratios (ie, the relative ratio of telomere repeat copy number to single-copy gene; interassay coefficient of variability, 2.3%). All DNA samples were run twice, each with triplicate wells. If the difference between the 2 runs was greater than 7%, samples were run a third time and the average of the 2 closest values was used for data analysis. DNA samples from the same participant were assayed as 1 batch, using the same reagent lots, and run on the same assay plate.

### Magnetic Resonance Imaging Acquisition

Magnetic resonance imaging (MRI) acquisition was carried out on a 3-T scanner (Siemens Verio; Siemens) with a 32-channel head coil. T1-weighted images were acquired using a 3-dimensional magnetization-prepared rapid gradient-echo sequence (176 sagittal slices; repetition time, 2300 milliseconds; echo time, 2.98 milliseconds; inversion time, 900 milliseconds; flip angle, 7°; field of view, 240 × 256 mm^2^; and matrix, 240 × 256; 1 × 1 × 1 mm^3^ voxels). Imaging hardware and console software (Syngo B17; Siemens Healthineers) were held constant throughout data collection.

### Processing of Structural Data

The T1-weighted MRIs were processed using FreeSurfer, version 5.1.0, to generate cortical surface models for measurements of CT.^53^ We chose the most general cross-sectional image processing procedure to enable baseline data analysis for cross-sectional study goals of the ReSource Project (eg, as used by Valk et al^54^) before the completion of data acquisition, which spanned more than 2 years. The exact processing steps of cortical reconstruction and volumetric segmentation are described in previous publications.^55,56,57^ Briefly, T1-weighted images were intensity normalized and skull stripped, followed by a tessellation of the gray matter/white matter cortical boundary and automated topologic correction. Surface deformation was performed along intensity gradients, placing borders of the inner (gray matter/white matter) and outer (gray matter/cerebrospinal fluid) cortical interfaces at the location where the greatest shift in intensity defined the transition to the other tissue class. Cortical thickness was calculated as the shortest distance from the gray matter/white matter boundary to the gray matter/cerebrospinal fluid boundary at each vertex on the tessellated surface and is reported in millimeters.^58^

### Preprocessing of Dependent Variables

Change in LTL was calculated as difference scores between each participant’s measurements from a set of consecutive testing times (ie, T1 minus T0, T2 minus T1, and T3 minus T2). Specifically, we calculated LTL change as the Verhulst *D* value^59^ (herein termed *D*LTL) to correct for regression to the mean instead of the common practice of controlling for baseline LTL because it has recently been suggested that the latter approach inflates type I error rates.^60^

For statistical analysis of CT, we generated participant-specific CT change maps (∆CT) by subtracting vertexwise thickness maps of subsequent measurement times. Thickness data at each vertex were normalized before change calculation by regressing out the effects of global thickness to emphasize relative region-specific change patterns.

All difference scores diverging more than 3 SDs from the sample mean difference score were defined as outliers. There were no outliers in the ΔCT data analyzed in the present study. Seven *D*LTL outliers were winsorized to the respective upper or lower boundaries of 3 SD. In addition, MRI data were excluded if they did not pass quality control by 2 independent expert raters (S.L.V. and B.C.B.) owing to excessive movement or artifacts in the T1-weighted MRI images (eTable 5 and eTable 6 in Supplement 2). Five scans were excluded because of low image quality.

### Statistical Analysis

Data analysis was conducted between September 23, 2016, and June 21, 2019. All longitudinal analyses were performed using multivariate linear mixed models, which are robust to unbalanced and incomplete data in longitudinal designs,^61^ allowing the inclusion of all eligible data points for a given analysis. Body mass index, age, and sex were selected as covariates owing to their established association with LTL^11,62,63^ and brain structure.^64^ We also included the variable time point to control for potential effects of the time of measurement on ∆CT. Detailed model descriptions can be found in eAppendix 1 in Supplement 2. All analyses were conducted with an α threshold of .05 or less.

The whole-brain, linear mixed-model analysis of CT data was performed using SurfStat for Matlab,^65^ first with the predictors *D*LTL and time only, and subsequently with the added variables of age, body mass index, and sex, to control for their potential influence. Statistical results were corrected for multiple comparisons following random field theory by means of a conservative cluster-determining threshold of *P* < .005 and familywise error corrections of *P* < .01 (2-tailed) for 20 mm full width at half maximum, smoothed surface–based thickness data, which correspond to the recently recommended threshold for the analysis of surface-based anatomic CT data.^66^ All follow-up analyses were conducted on cluster extent ∆CT in the region identified to be significantly associated with *D*LTL (eAppendix 1 in Supplement 2 provides further details). Effect sizes were calculated as effect size correlations *r* through the following formula^67^:


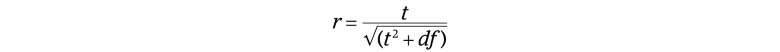
.

Linear mixed models for the analysis of the training intervention were fit using the function lmer of the package lme4^68^ for the statistics software R, version 3.5.1^69^ (eAppendix 1 in Supplement 2). The effect of any term of interest on *D*LTL was evaluated by comparing the fit of a full model with a reduced model lacking only the term of interest, by means of likelihood ratio tests.^70^

## Results

### 

#### Participants

Sample sizes and reasons for missing cases of all variables relevant to the current investigation are described in Figure 2 as well as eTable 5 and eTable 6 in Supplement 2. Of the 362 individuals randomly assigned to TCs or RCC, 30 participants dropped out before study initiation (initial sample, 332). For the analysis of the mental training intervention, data were available in 298 participants (175 women [58.7%]; mean [SD] age, 40.5 [9.3] years; 222 assigned to TCs, 76 to the RCC). For the analysis of ΔCT, all required covariates and MRI data were available in 67 RCC participants (37 women [55.2%]; mean [SD] age, 40.5 [9.3] years), providing 167 observations in total (eTable 5 and eTable 6 in Supplement 2). Mean (SD) LTL in T/S ratios at baseline was 1.016 (0.16) and individual-level *D*LTL ranged from −0.259 to 0.251. Further demographic characteristics are reported in the Table.

**Table.** Demographic Characteristics at Baseline and per Module^a^

Characteristic	Baseline	No Training	No Training MRI	Presence^b^	Affect^c^	Perspective^b^
Observations, No.	285	198	167	145	216	140
Women, No. (%)	166 (58.2)	118 (60.0)	89 (53.0)	85 (58.6)	124 (57.4)	79 (56.4)
Participants, No.	285	76	67	145	216	140
Women, No. (%)	166 (58.2)	46 (60.5)	37 (55.2)	85 (58.6)	125 (57.9)	79 (56.4)
Age, mean (SD), y	40.4 (9.3)	39.5 (9.3)	39.6 (9.0)	41.2 (9.3)	40.8 (9.3)	40.9 (9.6)
BMI, mean (SD)	23.5 (3.2)	23.9 (3.0)	23.9 (2.9)	23.5 (3.3)	23.4 (3.2)	23.6 (3.3)
LTL in T/S ratio, mean (SD)	1.016 (0.16)	1.043 (0.16)	1.040 (0.17)	1.002 (0.16)	0.996 (0.16)	1.011 (0.17)
Smokers, No. (%)	34 (11.9)	8 (10.5)	6 (9.0)	21 (14.5)	28 (13.0)	20 (14.3)
Practice, mean (SD), No./wk						
Meditation	NA	NA	NA	4.76 (1.17)	3.84 (1.31)	3.62 (1.23)
Dyad	NA	NA	NA	NA	3.80 (0.70)	3.51 (0.71)

Abbreviations: BMI, body mass index (calculated as weight in kilograms divided by height in meters squared); LTL, leukocyte telomere length; MRI, magnetic resonance imaging; NA, not applicable; T/S ratio, relative ratio of telomere repeat copy number to single-copy gene.

^a^ Summary statistics include all participants for whom LTL change data and covariate measures were available. An extensive description of baseline demographic characteristics was reported by Singer et al.^71^ eTable 4 in Supplement 2 details LTL difference scores per time point and module, and eAppendix 2 in Supplement 2 provides descriptive analyses.

^b^ Combined number of cohorts 1 and 2.

^c^ Combined number of training cohorts 1 to 3, resulting in a higher number than Presence and Perspective training.

#### LTL Change and CT

**Figure 3.**
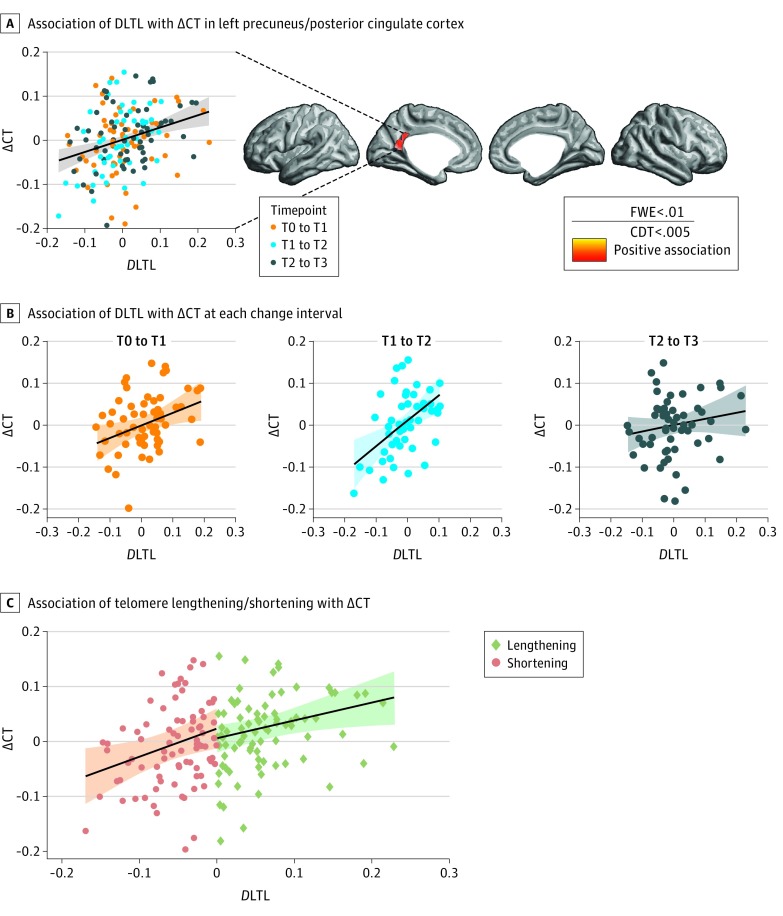
Association Between Leukocyte Telomere Length Change (*D*LTL) and Cortical Thickness Change (∆CT) A, *D*LTL was positively associated with ∆CT in the left precuneus/posterior cingulate cortex (PCC). Automated Anatomical Labeling atlas: 61% (59%) overlap with precuneus (primary region); 24% [26%] overlap with PCC (secondary region); percentages in brackets indicate results from analysis without age, body mass index, and sex. B, Association between *D*LTL and ∆CT at each change interval. C, Association of leukocyte telomere lengthening and shortening with ∆CT. For visual display through scatterplots, ∆CT in the cingulate/PCC region was averaged and plotted against *D*LTL. Each dot represents 1 observation rather than 1 participant. Up to 3 measures of *D*LTL were available from the same participant, each from a different change interval and controlled for by the linear mixed-model analysis. Displayed regression lines in panels B and C were derived from linear models fit independently for each subsample (B: 1 sample per time interval; C: separate samples for leukocyte telomere lengthening/shortening). Shaded areas represent 95% CIs. CDT indicates cluster-determining threshold; FWE, familywise error correction.

The first whole-brain analysis identified a significant association between *D*LTL and ∆CT in the left precuneus extending to the posterior cingulate cortex (PCC) (mean values of cluster extend test: *t*_164_ = 3.21; *P* < .001; *r* = 0.243; 59% overlap with precuneus, 26% overlap with PCC [Automated Anatomical Labeling atlas]) (Figure 3A). In repeating the analysis with the added variables age, body mass index, and sex, the same region was identified (mean *t*_161_ = 3.22; *P* < .001; *r* = 0.246; 61% overlap with precuneus, 24% overlap with PCC).

In subsequent follow-up cluster-level tests, the association between *D*LTL and ∆CT in the identified region was also significant when analyzed with an additional random slope term, as well as when controlling for change in leukocyte cell type counts (eAppendix 3 and eTable 1 in Supplement 2). Further follow-up analyses assessed the consistency of the observed phenomenon by testing whether the association between *D*LTL and ∆CT was reliable across the 3 intervals of change. Linear models showed that, when analyzed separately, change in LTL and CT were significantly associated at 2 of the 3 intervals (T0-T1: *t*_54_ = 2.60; *P* = .006; *r* = 0.334; T1-T2: *t*_48_ = 3.75; *P* < .001; *r* = 0.476; T2-T3: *t*_50_ = 1.28; *P* = .10; *r* = 0.178) (Figure 3B). In an exploratory analysis of change from baseline to 9 months, however, we did not find a significant association (*t*_56_ = 0.46; *P* = .31; *r* = 0.06) (eFigure 1 in Supplement 2).

Leukocyte telomere shortening and lengthening manifested themselves in a similar number of observations (Figure 3A, scatterplot). Examining these separately in follow-up analyses revealed that LTL change was positively associated with ∆CT regardless of direction, that is, leukocyte telomere shortening with a greater tendency for cortical thinning (*t*_77_ = 2.38; *P* = .01; *r* = 0.262), and lengthening with a greater tendency for cortical thickening (*t*_77_ = 2.42; *P* = .009; *r* = 0.266) (Figure 3C). Corresponding to the observed association of *D*LTL with ∆CT in the left hemisphere, exploratory analyses identified a contralateral subthreshold association with ∆CT in the right precuneus/PCC (eAppendix 4 and eFigure 3 in Supplement 2).

#### Mental Training Intervention

**Figure 4.**
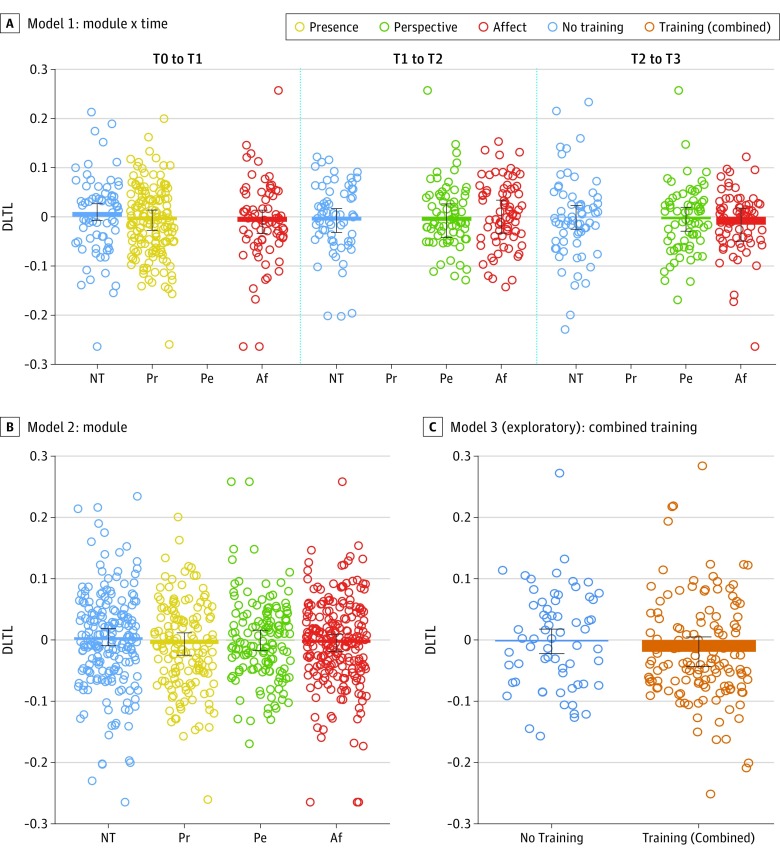
Raw and Estimated Change in Leukocyte Telomere Length (*D*LTL) per Training Module Estimated mean change derived from the linear mixed-model analyses detailed in the Methods section and eAppendix 1 in Supplement 2, with all covariates held constant at their mean. A, Estimates from training model 1, examining the interaction between module and time point of training. B, Estimates from training model 2, examining the mean effects of each module. C, Estimates from an exploratory analysis, examining change over the whole 9-month period. Each circle represents a raw LTL difference score, but the models were fit on LTL scores corrected for regression to the mean (*D*LTL). Error bars represent 95% CIs. Af indicates Affect; NT, no training; Pe, Perspective; and Pr, Presence.

Likelihood ratio tests showed no significant main effect of the training module by time interaction (χ^2^ = 3.26_3_; *P* = .35) or module alone on (χ^2^ = 2.20_3_; *P* = .53) *D*LTL (Figure 4A and B). We could therefore not pursue the hypothesis that systematic training-induced differences in LTL change would be associated with changes in brain structure. Estimated mean change in T/S ratios in model 2, our main model of interest, were, for no training, 0.004 (95% CI, −0.010 to 0.018); Presence, −0.007 (95% CI, −0.025 to 0.011); Affect, −0.005 (95% CI, −0.019 to 0.010); and for Perspective, −0.001 (95% CI, −0.017 to 0.016).

As a follow-up analysis, we explored whether the combined 9-month training period affected *D*LTL; this was not the case (*F*_1,196_ = 1.88; *P* = .17) (Figure 4C; eAppendix 1 in Supplement 2). Additional exploratory analyses showed that the effect of training remained nonsignificant even when considering practice frequency or participant personality or when focusing on within-participant differences (eAppendix 4, eTable 2, and eTable 3 in Supplement 2). Checking the residuals of the above models through visual inspection and diagnostic tests did not reveal any evidence for influential cases, systematic error, or multicollinearity (eAppendix 5 in Supplement 2).

## Discussion

With the present investigation, we sought to improve our understanding of the neural and psychological processes associated with short-term change in LTL through 2 steps. We first related *D*LTL to structural brain changes over 3 consecutive 3-month intervals in an RCC. Second, we assessed the effect of 3 distinct, 3-month mental training modules on *D*LTL.

Repeated measures of *D*LTL in RCC participants were associated with ∆CT in the left precuneus/PCC. When analyzed separately, the association was significant over 2 of the 3 consecutive, 3-month intervals. Precuneus and PCC were previously shown to be linked to LTL in a large, cross-sectional, population-based study.^7^ The longitudinal association between short-term changes in LTL and structural indices of the left precuneus/PCC noted in our study suggests a dynamic association between the 2 phenomena.

To our knowledge, there is only 1 other study associating change in LTL and brain structure. Leukocyte telomere shortening and loss of structural integrity were found to correlate in older adults at a single, 2.9-year follow-up measurement.^72^ In contrast, we observed similar rates of leukocyte telomere shortening and lengthening. Shortening was related to a greater tendency for cortical thinning and lengthening was related to a greater tendency for thickening, even when analyzed separately. The identified association was therefore not predominantly driven by aging-related decline. In an exploratory analysis, *D*LTL from baseline to the 9-month follow-up measurement showed no association with ∆CT, and we cannot say with certainty why this occurred. Our results may, however, indicate that short-term LTL change does not follow a continuous trajectory but rather represents transient change.

Telomere fluctuations that average out over time have previously been observed,^73^ but there is currently no model for the potential underlying biological processes. One mechanism that could be responsible for the associated changes in LTL and CT may be the action of the cellular enzyme telomerase. Telomerase counteracts telomere shortening by replenishing telomeric DNA during cell division.^22,74^ Increases in telomerase activity have been associated with short-term telomere lengthening, most notably following 6-month physical training protocols,^20,75^ and may also have mediated the lengthening observed in this study. Likewise, cortical thickening could be facilitated by an increased telomerase-associated proliferative capacity of critical support cells in the brain capable of undergoing mitosis in the adult, resulting in greater tissue volume, as previously proposed.^7^ Heightened telomerase activity may therefore lead to associated increases in LTL and CT, assuming that central and peripheral telomerase activity are related. In line with this reasoning, telomerase activity was previously found to correlate positively with hippocampal volume, although not with LTL, in a small sample of patients with major depressive disorder.^76^ While it is unclear which neurobiological mechanisms exactly underlie cortical thickening in the age group evaluated herein, these authors described the potential gliogenesis-enhancing and neuroprotective effects of telomerase in more detail. Concomitant telomere shortening and cortical thinning, if not reflective of aging-related decline, could similarly result from accelerated telomere attrition and decreased cellular proliferation under lowered telomerase activity. In the present study, we could not assess whether such fluctuations in telomerase activity occurred and, if so, whether they result from endogenous processes or external influences, such as the lifestyle factors stress or physical exercise.^20,77^ The same lifestyle variables have also been directly associated with differences in telomere length^15,20^ and brain structure,^78,79^ and therefore provide an explanatory framework for the observed concomitant change that may not involve mediation through telomerase activity.

The present study identified significantly less widespread association between change in LTL and brain structure than was reported by King and colleagues.^7^ Likely reasons for this divergence include King and colleagues’^7^ region of interest–based, cross-sectional study design compared with our whole-brain longitudinal analysis approach, as well as their older and considerably larger sample (N = 1960), which sensitized their analyses to associations with very small effect sizes of *R*^2^ less than 1%. Owing to our comparatively smaller participant sample, the present study was not equipped to detect similarly sized effects. Nonetheless, a specific association between LTL change and thickness change in the left precuneus/PCC was identified. The precuneus/PCC region is a prominent metabolic hub and central node of the default mode network.^80^ The observed change in CT may thus, for example, reflect a particularly strong susceptibility to telomerase-related synaptic plasticity grounded in this region’s unique metabolic and connectional properties. Moreover, it is notable that precuneus/PCC structure and function are impaired in age-related neurodegenerative disorders, such as Alzheimer disease.^8,9,81^ In cross-sectional studies, shorter telomeres have been associated with similar age-related conditions.^3,4,5,6^ The specific coupling of short-term LTL change with ΔCT in the precuneus/PCC could be a window to cellular processes implicated in the development of age-related diseases. The long-term dynamics of this association should be investigated in future research using targeted, longitudinal population studies tracking aging-related changes in telomere length in combination with multimodal MRI.

Contrary to our predictions, we found no effect of contemplative mental training on change in LTL. This result contrasts with the findings of 2 preceding interventions that identified telomere lengthening in relation to meditation.^21^ The first study found telomere lengthening in men with low-risk prostate cancer following a relatively broad, 5-year lifestyle intervention.^32^ The differing participant population and intervention design may account for the diverging outcomes. The other study found telomere lengthening in healthy, middle-aged participants after a short, but intense, 1-month residential training retreat, which may have unique benefits.^31^ In contrast, our results provide what we believe to be the largest body of evidence that longitudinal contemplative mental training does not systematically lengthen leukocyte telomeres of healthy adults. Because we detected no mean attrition in the RCC over the entire 9-month study period, we cannot make any conclusions regarding potential relative maintenance of telomere length through mental training.

Effects of the ReSource Project training modules on CT alone have been reported.^42^ Because we found evidence for an association between CT and LTL on the individual participant level, but no effect of training on LTL, it appears that the mechanism underlying the herein observed association is independent of the capacities trained during the intervention. Other work showed that the ReSource Project intervention reduced physiological responses to acute stress and perceived social connectedness.^45,47^ Our present findings therefore provide indirect evidence that alleviating these psychophysiological strains does not lead to short-term telomere lengthening in healthy individuals.

### Limitations

The present study has limitations. The effect of measurement error on short-term LTL change, particularly lengthening, is controversial.^23,24^ In the present sample, given our interassay coefficient of variability of 2.3% and mean LTL T/S ratio of approximately 1.0, measurement error should, on average, be 0.023. On an individual participant level, observed change ranged from −0.259 to 0.251 and is therefore unlikely predominantly attributable to measurement error, although lengthening or shortening classifications of particularly small change values could be distorted. Regarding the association with CT, measurement error should increase the risk of type II rather than type I error. The herein detected robust associations at multiple intervals of change are therefore unlikely attributable to measurement error. Rather, stronger associations may be found if measurement error was minimized. A further limitation is that, by evaluating structural change using CT, we were unable to examine potential associations with subcortical or allocortical regions, such as the hippocampus, which has been associated with LTL in cross-sectional studies.^82^

## Conclusions

Our findings contribute to the evidence that LTL changes more dynamically on the individual level than previously thought and indicate that short-term LTL change is associated with structural brain alteration. Further studies will need to identify the long-term implications of such changes in relation to cellular aging and the development of neurodegenerative disorders, as well as how to activate protective processes that influence LTL. In contrast to our hypotheses and some earlier reports, LTL of healthy adults was not influenced through contemplative mental training over 9 months.

## Notes

Supplement 1. **Trial Protocol**

Supplement 2. **eAppendix 1.** Detailed Methodology **eAppendix 2.** Descriptive Analyses **eAppendix 3.** Random Slopes Model **eTable 1.** Correlations Between Change in LTL and Leukocytic Cell Type Concentrations in the RCC **eAppendix 4.** Exploratory Analyses **eAppendix 5.** Residual Checks **eFigure 1.** Change in Telomere Length and Cortical Thickness From Baseline to 9-Month Follow-up **eFigure 2.** Relative Difference in Telomere Length per Training Module **eFigure 3.** *D*LTL and ∆CT in Right Precuneus/PCC ROI **eTable 2.** Mean (SD) of Baseline Personality Trait Scores **eTable 3.** Results of Exploratory Analyses **eTable 4.** Mean (SD) Unmodelled Change in LTL After Each Training Module **eTable 5.** Available Samples and Reasons for Missing Values in the Raw Data **eTable 6.** Available Samples and Reasons for Missing Values in Change Scores **eReferences**

Supplement 3. **Data Sharing Statement**
