# Supplementary material for: Association of Short-term Change in Leukocyte Telomere Length With Cortical Thickness and Outcomes of Mental Training Among Healthy Adults: A Randomized Clinical Trial
Source: JAMA Netw Open. 2019 Sep 25;2(9):e199687. doi: 10.1001/jamanetworkopen.2019.9687 (PMC6763984; doi:10.1001/jamanetworkopen.2019.9687)
Supplement: Supplement 1. — Trial Protocol [file jamanetwopen-2-e199687-s001.pdf]

Translated from German – Redacted Clinical Protocol for Journal Use

Application to the ethics committee of the University of Leipzig

ADDENDUM III[1]

„ReSource“ Study on the Trainability of Socio-Affective and Socio-Cognitive Skills

Prof. Dr Tania Singer

Director, Max Planck Institute for Human Cognitive and Brain Sciences

Department of Social Neuroscience
Stephanstr. 1a, 04103 Leipzig, Germany
Birthdate: 08.12.1969, German
Tel.: 0049 341 / 9940 149
Fax: 0049 341 / 9940 2356
E-mail: singer@cbs.mpg.de

[1] This document is the 3rd revision of the original ethical application; for more details on the study see also Singer, T., Kok, B. E., Bornemann, B., Zurborg, S., Bolz, M., & Bochow, C. (2016). *The ReSource Project: Background, design, samples, and measurements.* (2nd ed.). Leipzig: Max Planck Institute for Human Cognitive and Brain Sciences.

1 TABLE OF CONTENTS

1 Formalities 6
1.1 Name of Project 6
1.2 Applicant Name 6
1.3 Involved Scientists 6
1.4 Funding and ERC Grant 11
1.5 Ethic Applications to other Ethic Committees 11
1.6 Revision 11
1.7 Physicians 11
2 Aim of the Study and Current Application 12
3 Scientific Background of the Study and Description of Training Content 14
3.1 Definition of Compassion 14
3.2 Previous Training Programs on Compassion 16
3.3 Scientific Evaluation of Compassion Training 16
3.4 Overview of the Design and Cohort Distribution in the Study 17
3.4.1 Overview of the Study Design 17
3.4.2 Training Effort for Participants 18
3.5 Description of the Training Protocol 19
3.5.1 Content of the Presence Module 19
3.5.2 Content of the Perspective Module 20
3.5.3 Content of the Affect Module 21
3.6 Teachers 23
3.7 Technical Support 23
3.7.1 Smartphones 23
3.7.2 Online Platform 23
4 Description of Paradigms and Methods of Investigation 25
4.1 Imaging by means of Magnetic Resonance Tomography 25
4.1.1 Structural Imaging 25
4.1.2 Functional Imaging 25
4.2 Paradigms in Virtual Scenarios 25
4.3 Behavioral Computer Experiments 25
4.3.1 Perception Sensitivity and Meta-Cognition 25
4.3.2 Inhibition and Alerting (Stopp-Signal-Reaction-Time-Task) 25

4.3.3 Working Memory and Meta-Cognition 25
4.3.4 Attentional Blink Paradigm 26
4.3.5 Task-switching Paradigm 26
4.3.6 Emotional Granularity Task 26
4.3.7 Socio-affective Video Task (SoVT) 26
4.3.8 Samson’s Test 26
4.3.9 Economic Games 26
4.3.10 Self-complexity Task 26
4.3.11 Self-reference Task 26
4.3.12 Task for the Assessment of „Mind-Wandering“ 26
4.3.13 Donation Task 26
4.4 Questionnaires 26
4.5 Subjective Methods 26
4.5.1 Weekly Experience Sampling via the Internet platform 26
4.5.2 Daily questions about the exercises via the Internet platform or the smartphone .26
4.5.3 Collection of Experience-sampling Data with Smartphones 27
4.5.4 Observer Reports 27
4.6 Stress Physiology 27
4.6.1 Diurnal Cortisol Profiles 27
4.6.2 Trier Social Stress Test (TSST) 27
4.7 Assessment of autonomic Functions 28
4.7.1 Vagal Tone 28
4.7.2 Autoregulation of the state of excitation 28
4.7.3 Heart Beat Perception 28
4.7.4 Measurement of autonomous functions and movement behavior during meditation 28
4.8 Qualitative Interviews 28
4.8.1 Voluntary Interviews on the Subjective Experiences of the Training 28
4.8.2 Voluntary Elicitation Interviews 28
4.9 Summary of the Time Required for the Measurements 28
4.9.1 Magnetic Resonance Imaging (MRI) and Virtual Reality (VR) Scenarios 28
4.9.2 Behavioral computer paradigms 29
4.9.3 Questionnaires 30

4.9.4 Subjective Methods 30
4.9.5 Stress Physiology 31
4.9.6 Measurement of Autonomic Functions 31
4.9.7 Qualitative Interviews 31
5 Introduction from Addendum I 31
6 Biological Analyses 32
6.1 Summary of planned blood samples 32
6.2 Genetic analyses of gene variants / polymorphisms 32
6.3 Cytokine analyses from blood samples (during the training process, pre-post acute stress) 32
6.4 Immunoglobulin A Analysis from saliva samples (pre-post acute stress) 32
6.5 Oxytocin analyses from blood samples (pre-post acute stress, pre-post of a mental training session) 32
6.6 Cortisol analyses from hair samples (during training) 33
6.7 Telomere length (during training) 33
6.8 Number of cell types in the blood 33
7 Number of subjects, recruitment, exclusion criteria, payment, terms of cancelling participation 33
7.1 Number of Subjects 33
7.2 Recruitment process 34
7.3 Exclusion Criteria 34
7.4 Payment and other benefits for the subjects 36
7.5 Terms of cancelling the study participation 37
8 Risks for the subjects, planned arrangements regarding risk reduction, subject insurance 37
8.1 Possible Risks and Complications; Dealing with Complications 37
8.1.1 Risk of Training 37
8.1.2 Risks of Magnetic Resonance Tomography 38
8.1.3 Risk of Pain Paradigm 38
8.1.4 Deception During Economic Games 38
8.1.5 Virtual Reality Scenarios 38
8.1.6 Behavioral Experiments 38
8.1.7 Trier Social Stress Test 38
8.1.8 Blood Collection 38
8.2 Further Treatment after End of Study 38

8.3 Risk-Benefit Assessment 38
8.4 Participant and Accident Insurance 39
9 Data Collection, Data Processing, Data Usage, and Data Protection 40
10 Declaration that the “Declaration of the World Medical Association of Helsinki” is known 40
11 References 41

1 Formalities

1.1 Name of Project

„ReSource"-Study on Trainability of Socio-affective and Socio-cognitive Skills

Short: ReSource Study

1.2 Applicant Name

Prof. Dr Tania Singer
Direktor, Max Planck Institute for Human Cognitive and Brain Science
Department of Social Neuroscience
Stephanstr. 1a, 04103 Leipzig, Germany
Birthday: 08.12.1969, German
Tel.: 0341 / 9940 149
Fax: 0341 / 9940 2356
E-mail: singer@cbs.mpg.de

1.3 Involved Scientists[2]

Boris Bernhardt, PhD (moved to Canada)
Postdoc at the Max Planck Institute for Human Cognitive and Brain Science
Department of Social Neuroscience
Stephanstr. 1a, 04103 Leipzig, Germany
Birthdate. 23.12.1981, German
Tel.: 0049 341 / 9940 2658
Fax: 0049 341 / 9940 2356
E-mail: bernhardt@cbs.mpg.de

Cade McCall, PhD
Postdoc at the Max Planck Institute for Human Cognitive and Brain Science
Department of Social Neuroscience
Stephanstr. 1a, 04103 Leipzig
Birthdate: 29.05.1974, American (USA)
Tel.: 0049 341 / 9940 2683
Fax: 0341 / 9940 2356
E-mail: mccall@cbs.mpg.de

---

[2] The paragraph lists researchers that were involved in the study at the time of application. Note that due to the longitudinal nature of the project, members of the group left and new personnel were hired over the course of the study.

Joshua Grant, PhD
Postdoc at the Max Planck Institute for Human Cognitive and Brain Science
Department of Social Neuroscience
Stephanstr. 1a, 04103 Leipzig, Germany
Birthdate: 06.05.1974, Canadian
Tel.: 0049 341 / 9940 2653
Fax: 0049 341 / 9940 2356
E-mail: grant@cbs.mpg.de

Dr Veronika Engert
Postdoc at the Max Planck Institute for Human Cognitive and Brain Science
Department of Social Neuroscience
Stephanstr. 1a, 04103 Leipzig, Germany
Birthdate: 15.06.1974, German
Tel.: 0049 341 / 9940 268
Fax: 0049 341 / 9940 2356
E-mail: engert@cbs.mpg.de

Haakon Engen, Clinical Psychologist
PhD candidate at the Max Planck Institute for Human Cognitive and Brain Science
Department of Social Neuroscience
Stephanstr. 1a, 04103 Leipzig, Germany
Birthdate: 04.08.1982, Norwegian
Tel.: 0049 341 / 9940 2655
Fax: 0049 341 / 9940 2356
E-mail: engen@cbs.mpg.de

Bethany Kok, PhD
Postdoc at the Max Planck Institute for Human Cognitive and Brain Science
Department of Social Neuroscience
Stephanstr. 1a, 04103 Leipzig, Germany
Birthdate: 03.05.1984, American (USA)
Tel.: 0049 341 / 9940 2682
Fax: 0049 341 / 9940 2356
E-mail: bethkok@cbs.mpg.de

Dr Philipp Kanske
Postdoc at the Max Planck Institute for Human Cognitive and Brain Science
Department of Social Neuroscience
Stephanstr. 1a, 04103 Leipzig, Germany
Birthdate: 21.01.1980, German
Tel.: 0049 341 / 9940 2693
Fax: 0049 341 / 9940 2356

E-mail: kanske@cbs.mpg.de

Dr Anne Böckler
Postdoc at the Max Planck Institute for Human Cognitive and Brain Science
Department of Social Neuroscience
Stephanstr. 1a, 04103 Leipzig, Germany
Birthdate: 31.08.81, German
Tel.: 0049 341 / 9940 2688
Fax: 0049 341 / 9940 2356
E-mail: aboeckler@cbs.mpg.de

Dr Anita Tusche
Postdoc at the Max Planck Institute for Human Cognitive and Brain Science
Department of Social Neuroscience
Stephanstr. 1a, 04103 Leipzig, Germany
Birthdate: 22.11.81, German
Tel.: 0341 / 9940 2690
Fax: 0341 / 9940 2356
E-Mail: tusche@cbs.mpg.de

Dr Marisa Przyrembel
Postdoc at the Max Planck Institute for Human Cognitive and Brain Science
Department of Social Neuroscience, Satellite Lab Berlin, Humboldt University Berlin, Haus 5, Philippstrasse 13, 10099 Berlin, Germany
Birthdate: 13.09.80, German
Tel.: 0049 30 2093 46185
Fax: 0049 30 2093 46189
E-mail: przyrembel@cbs.mpg.de

Sofie Valk, Master of Science in Brain and Cognitive Sciences
PhD candidate at the Max Planck Institute for Human Cognitive and Brain Science
Department of Social Neuroscience
Stephanstr. 1a, 04103 Leipzig, Germany
Birthdate: 15.10.87, Dutch
Tel.: 0049 341 / 9940 2691
Fax: 0049 341 / 9940 2356
E-mail: valk@cbs.mpg.de

Lea Hildebrandt, Master of Science in Brain and Cognitive Sciences
PhD candidate at the Max Planck Institute for Human Cognitive and Brain Science
Department of Social Neuroscience
Stephanstr. 1a, 04103 Leipzig, Germany
Birthdate: 27.02.1986, German
Tel.: 0049 341 / 9940 2691

Fax: 0049 341 / 9940 2356
E-mail: hildebrandt@cbs.mpg.de

Fynn-Mathis Trautwein, Graduate Psychologist
PhD candidate at the Max Planck Institute for Human Cognitive and Brain Science
Department of Social Neuroscience
Stephanstr. 1a, 04103 Leipzig, Germany
Birthdate: 30.04.1985, German
Tel.: 0049 341 / 9940 2301
Fax: 0049 341 / 9940 2356
E-mail: trautwein@cbs.mpg.de

Boris Bornemann, Graduate Psychologist
Project Assistant and since January 2013 PhD candidate at the Max Planck Institute for Human Cognitive and Brain Science
Department of Social Neuroscience
Stephanstr. 1a, 04103 Leipzig, Germany
Birthdate: 16.05.1984, German
Tel.: 0049 341 / 9940 2533
Fax: 0049 341 / 9940 2356
E-mail: bornemann@cbs.mpg.de

Anna-Lena Lumma, Master of Science in Cognitive Science
PhD candidate at the Max Planck Institute for Human Cognitive and Brain Science
Department of Social Neuroscience
Stephanstr. 1a, 04103 Leipzig, Germany
Birhtdate: 1.10.1986, German
Tel.: 0049 341 / 9940 2662
Fax: 0049 341 / 9940 2356
E-mail: lumma@cbs.mpg.de

Matthias Bolz, Graduate Psychologist
Lab Manager at the Max Planck Institute for Human Cognitive and Brain Science
Department of Social Neuroscience
Stephanstr. 1a, 04103 Leipzig, Germany
Birthdate: 8.11. 1970, German
Tel.: 0341 / 9940 2533
Fax: 0341 / 9940 2356
E-mail: bolz@cbs.mpg.de

Dr Christina Bochow (née Zube)
Lab Manager at the Max Planck Institute for Human Cognitive and Brain Science
Department of Social Neuroscience, Satellite Lab Berlin, Humboldt University Berlin, Haus 5, Philippstrasse 13, 10099 Berlin, Germany

Birthdate: 10.05.1977, German
Tel.: 0049 30 2093 46180
Fax: 0049 30 2093 46189
E-mail: zube@cbs.mpg.de/bochow@cbs.mpg.de

Astrid Ackermann
Lab Manager at the Max Planck Institute for Human Cognitive and Brain Science
Department of Social Neuroscience, Satellite Lab Berlin, Humboldt University Berlin, Haus 5, Philippstrasse 13, 10099 Berlin, Germany
Birthdate: 02.12.81, German
Tel.: 0049 30 2093 46180
Fax: 0049 30 2093 46189
E-mail: aackermann@cbs.mpg.de

Willi Zeidler, Graduate Psychologist
Lab Manager at the Max Planck Institute for Human Cognitive and Brain Science
Department of Social Neuroscience, Satellite Lab Berlin, Humboldt University Berlin, Haus 5, Philippstrasse 13, 10099 Berlin, Germany
Birthdate: 07.01.1978, German
Tel.: 0049 30 2093 46185
Fax: 0049 30 2093 46189
E-mail: zeidler@cbs.mpg.de

Leon Skottnik, Master of Science in Cognitive Neuroscience
PhD candidate at the Max Planck Institute for Human Cognitive and Brain Science
Department of Social Neuroscience
Stephanstr. 1a, 04103 Leipzig, Germany
Birthdate: 10.08.1988, German
Tel.: 0049 341 / 9940 2661
Fax: 0049 341 / 9940 2356

PD Dr Peter Kovacs
Universtiy of Leipzig
Medical Faculty
Interdisciplinary Center for Clinical Research [Interdisziplinäres Zentrum für Klinische Forschung (IZKF)] Leipzig, Germany
Liebigstraße 21
04103 Leipzig, Germany
Birthdate: 08.07.1970, Slovak
Tel.: 0049 341 97-15892
Fax: 0049 341 97-15949
E-mail: peter.kovacs@medizin.uni-leipzig.de

1.4 Funding and ERC Grant

The planned project is funded by the European Research Council (ERC, Reference: ERC-2007-StG; Grant Agreement Number: 205557) and the Max Planck Society.

1.5 Ethic Applications to other Ethic Committees

We have submitted a similar ethics application for the same project to the Research Ethics Committee of the Humboldt University in Berlin. The approval number is 2013-02.

Address:
Ethikkommission der Humboldt Universität zu Berlin
[Research Ethics Committee of the Humboldt University Berlin]
Mathematisch - Naturwissenschaftliche Fakultät II
Institut für Psychologie
Unter den Linden 6
10099 Berlin, Germany

In the context of the approval of the ERC Grant (ERC-2007-StG; Grant Agreement Number: 205557), which forms the basis for this study, a general ethical application for the European Union was submitted several years ago and has been reviewed and approved by the European Union. The present application is now a more detailed application for the respective local ethics committees.

1.6 Revision

This application is the third Addendum to the ethics application # 376-12-05112012.

1.7 Physicians

As the Ethics Committee has already been informed in the letter of 05 April 2012 by the Max Planck Institute in Leipzig, Anna Kosatschek has been the new doctor at the institute since 01.04.2013 and is also the responsible physician for this study.

Since Mrs. Anna Kosatschek is now on maternity leave, Dr med. Leonie Lampe will be responsible for the medical-related aspects of the institute and for this project until her return. Tel: 0049 341 9940 2622; E-mail: lampe@cbs.mpg.de

2 Aim of the Study and Current Application

The main goal of the planned research project (called the "ReSource" Study) is the scientific evaluation of an intervention on mental training of socio-affective and cognitive abilities. The project described in this application is a comprehensive multi-month longitudinal study. **A similar request for the study pilot of this project has already been approved by the committee (# 225-12-ff-02072012) and the Research Ethics Committee of the Humboldt University of Berlin (# 2012-26-F).**

We want to train study participants over several months. The training consists of three training blocks (Presence, Perspective, and Affect). Each of these blocks lasts eight weeks[3]. Before, after and sometimes in between the blocks we would like to examine the effects of the training by means of an interdisciplinary approach and using a wide range of methods.

A major research focus of this study is the plasticity of the social brain and the ability to train socio-emotional skills such as empathy, compassion, emotion regulation, cognitive perspective taking, mindfulness, attention, and memory. We would like to assess whether a long-term training leads to functional and structural changes in the brain as well as to hormonal, health-related, and behavioral changes.

We use experience sampling methods to assess daily changes in subjective well-being. We also want to use behavioral experiments, magnetic resonance tomography (MRI) experiments and psycho-physiological measurements (stress physiology, measurements of the autonomic nervous system). This is extended by experiments in virtual environments, as it allows the examination of participants in a complex and rather naturalistic environment.

Based on structural MRI data, we will assess long-term changes in cortical and subcortical networks of subjects participating in a socio-emotional and cognitive training. Dynamic changes of functional MRI signals, which are obtained during several affective and cognitive tasks, allow an examination of the relationships between functional and structural brain network re-organization as a result of the training. In addition, we examine whether specific psychological characteristics and certain initial brain network structures can be used to predict individual differences in the trainings results of the subjects. We use a variety of models from the field of social psychology and experimental economics, and link them to paradigms in virtual reality worlds that facilitate the measurement of explicit and implicit behavioral and motivational tendencies, to test for training-related changes in, among other things, altruistic behavior, affect regulation, and motivation.

[3] Please note that the length of each module was in total 13 weeks. During the first eight weeks new content was introduced every week. During the final 5 weeks participants were also tested but sessions continued and content was repeated and deepened.

In summary, we are looking for evidence of socio-cognitive and emotional changes on levels of brain plasticity, autonomous physiology, endocrine functions, reported subjective experience, and behavior in standardized laboratory situations.

The expected results from this large-scale longitudinal study would be of great importance not only for basic research in the field of plasticity research, but also for the design of scientifically sound and effective training programs for schools and economic organizations, as well as later on for the treatment of patients who suffer from severe social deficits, such as autistic or psychopathic patients.

A total number of *N* = 330[4] subjects are measured. Of those, 160 subjects are in the test group, which runs through the training. There is a retest control group, which does not participate in the training, but is tested in same intervals (*N* = 30) during the same period.
After the training blocks of the test groups up to *N* = 60 additional control group subjects are tested, which will not receive any training either.
In addition, we have another training control group (active control group, *N* = 80). This cohort receives a shorter training (only the affect module) and has 2–3 measurement time points (i. e. T0 baseline testing, 1 Affect Module retreat, 13 weeks Affect Module training, T1 testing, and voluntary T2 follow-up testing).

We will first describe the training protocol in more detail before we describe the experimental methods. For more detailed timing-related overviews, please refer to Chapter 3.4.2 and Chapter 4.8. We will illustrate the effort for the study participants by splitting it into training effort and testing effort.

[4] As written in the book by Singer et al., 2016, we had a total of 332 participants as two of them dropped out or were excluded very early during the T0 baseline testing phase and were replaced by two other persons from the waiting list. Thus, we had N=81 subjects in the second training cohort and *N*=81subjects in the active control cohort.

3 Scientific Background of the Study and Description of Training Content

3.1 Definition of Compassion

Note: The scientific background presented in this section has already been described in a similar form in a previous ethics application (# 225-12-ff-02072012) and is described in more detail here.

We distinguish a narrow definition of compassion, which is a motivational-emotional state, and a broad definition of compassion that encompasses both cognitive and socio-affective abilities and describes a particular way of perception and attitude to reality and to life (Merleau-Ponty, 1945, Varela, 1991).

In the narrow sense, compassion can be defined as the feeling that we have when we experience the suffering of another, coupled with the motivation to alleviate this suffering (Goetz et al., 2010, Singer & Lamm, 2009). The broader understanding of compassion, which is a certain skill set leading to a certain stable state of mind, can be interpreted as the interaction of a set of cognitive and socio-affective abilities and dispositions. This is depicted in the ReSource Model in Figure 1. This model forms the basis for our training concept.

The following chapter explains the respective domains (Presence, Perspective, and Affect) presented in the model as well as their cognitive and socio-affective sub-processes and their role for the realization of compassion in a broader sense. In chapter 3.5, we present the training protocol in more detail.

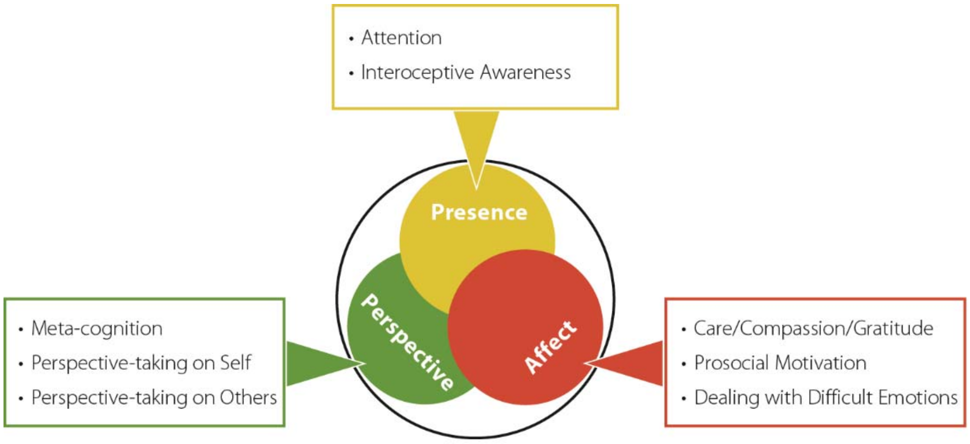

Figure 1: Model regarding the characteristics and skills that are relevant for compassion.

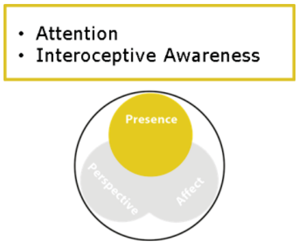

We call the first module Presence. It aims at cultivating both the ability to direct attention to the present moment and also to intercept, that is to train the awareness of internal-physical processes. In addition, the ability to direct attention is necessary for a clear and precise perception of the environment, for the stabilization of mental states and intentions, and thus for targeted behavior (Posner & Rothbart, 2009; Rueda et al., 2005; Tang et al., 2007, Vohs & Baumeister, 2011). Interoceptive awareness is an important basis for the recognition of emotions in oneself and others and for the effective regulation of emotions (Critchley et al., 2004, Ernst et al., 2012, Herbert et al., 2011, Singer et al , Sze et al., 2010; Terasawa et al., 2011).

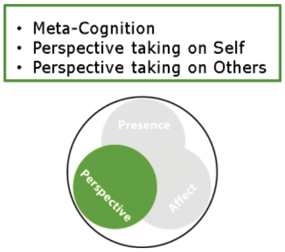

The second module is called Perspective. Perspective taking means the ability to distance oneself from one's own thoughts, values, and reaction tendencies and to take alternative positions. This ability can be divided into three sub-processes: meta-cognition, perspective taking on oneself, and perspective taking on others. Meta-cognition here, means the ability to understand thoughts as mental events, whose development, course, and consequences can be observed as a whole within the organism.

Such a relationship to one's own thoughts increases the margin for emotional and behavioral responses to these thoughts (Hayes & Pierson, 2005) and is considered a characteristic of mental health (Bach & Hayes, 2002, Luoma & Hayes, 2002).

While individual thoughts are observed and reflected in the context of meta-cognition, one’s own different personality parts and inner aspects are observed and reflected on in the context of perspective taking on oneself. The ability to have a distance to inner parts allows for an easier, more flexible handling of one's own reactions, as well as a better integration of different self-components (Berne, 2006; Mones & Schwartz, 2007; Schwartz, 2008). Perspective taking on others refers to the ability to understand other people's opinions, values, feelings, and personality traits, even if they differ greatly from one's own. In the field of psychology and neuroscience this is commonly referred to as "Theory of Mind" (ToM) or cognitive perspective taking (e.g., Gallagher & Frith, 2003; Sodian & Kristen, 2010). The development of this ability is central to understanding of the social world, as well as constructive and supportive social relationships.

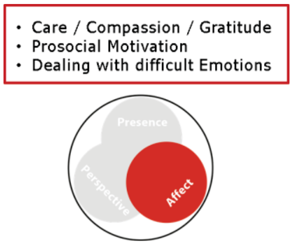

In the Affect Module, we consider the capacity for emotional acceptance, the ability and inclination to exalt benevolent feelings ("opening the heart" (note: care, loving kindness, compassion, gratitude)) and the extent of prosocial motivation. Emotional acceptance refers to an attentive and accepting approach to difficult

emotions. This type of processing has positive effects on health (e.g., emotion-related, somatic stress) (Low et al., 2008) and enables constructive coping when faced with difficult emotions and their triggers (Thompson, 1994). This is the prerequisite for both the benevolent and supportive approach to one's own person, as well as to others. "Opening of the heart" is the activation of the "care" or "affiliation" system (Depue & Morrone-Strupinsky, 2005; Panksepp, 2006), i.e. those psycho-biological processes, which have developed to be able to take care of offspring, or to care for a romantic partner and other members of the family as well as aspects of self-compassion. The activation of this system increases the capacity and inclination of the individual to feel constructive, benevolent, and loving feelings for oneself and for others (Gilbert & Procter, 2006). Prosocial motivation is the lasting ability to behave in a way, which promotes the well-being of other persons (Batson, 1987)

3.2 Previous Training Programs on Compassion

3.3 Scientific Evaluation of Compassion Training

Note: This chapter was already described in a previous ethics application (# 225-12-ff-02072012).

Compassion research is still quite young, but has recently shown some first important results, which we will present here briefly. For example, we have shown in a previous study (Leiberg et al., 2011) that one week of compassion training increases prosocial behavior in a standardized game situation. Pace et al. (2009) examined the effectiveness of a 6-week compassion training program. They were able to show that participants who practiced a lot showed a decrease in the physiological stress response (interleukin-6 level) and the subjective stress experience in a standardized social stress situation (the Trier Social Stress Test (TSST)). Klimecki et al. (2013) investigated how a one-week compassion training program changes the neural and emotional responses to the suffering of others. Prior to compassion training, participants showed negative affect (empathically accepting the affect of the observed person), associated with activations in brain regions associated with empathy for pain (among others: anterior insula and anterior-medial cingulate cortex). After compassion training, subjects responded rather with feelings of caring and readiness to help, and both were experiences as positive feelings and associated with the activation of a brain network associated with positive emotions and affiliation (among others: medial orbitofrontal cortex, putamen, pallidum, and ventral tegmentum).

A compassion-based intervention led to significant changes in symptoms in patients with heightened self-criticism and shame such as a reduction in depression, anxiety, harmful self-criticism, shame, inferiority, and submissive behaviors, and improved the patient's ability to self-soothe and to focus on feelings of warmth and safety (Gilbert & Procter, 2006).

Meta-analyses on the effect of accepting and compassionate attention to body sensations, thoughts and feelings in MBSR courses (Chiesa & Serretti, 2009, Grossman et al., 2004, Ledesma & Kumano, 2009) demonstrated many positive effects on physical and mental health:

Reduced pain perception, increased physical wellbeing, and generally increased life satisfaction.

3.4 Overview of the Design and Cohort Distribution in the Study

3.4.1 Overview of the Study Design

The training consists of three parts, which are based on the above-described areas of characteristics or abilities (Presence, Perspective, and Affect). Each training block begins with a 3-day retreat and then lasts 8 weeks[1], during which time the subjects meet for weekly sessions. The subjects are measured over a period of 5 weeks at the time T0 (before the training) and after the three training blocks (T1, T2, T3) and again about 4.5 months[5] later (T4, "follow-up") (MRI experiments, behavioral experiments, etc.) (see Figure 2). During this period, however, the participants still meet for weekly sessions during which the content of the course is further deepened and repeated.

There are two training cohorts. Both begin the training with the Presence Module, since the trained skills of attention control and interoception of this module form the basis for the abilities conveyed in the other two modules. Training cohort 1 then trains the other modules in the order of Affect-Perspective, while training cohort 2 trains the other modules in the Perspective-Affect. By having these two groups, sequence effects can be estimated in the training results and results at time point T2 allows for a direct comparison of the specific effects caused by socio-cognitive versus socio-affective mental training (e.g., inclusion of active control groups).

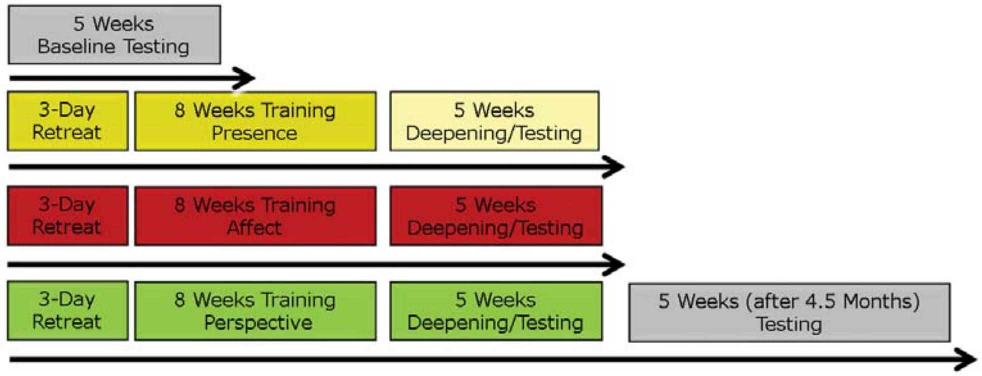

Figure 2: Overview over the study design shown for a participant from training cohort 1 (please note that the order of the green and red modules is interchanged for training cohort 2)[5]

Due to the limited capacity of the scanner, the two training groups begin their respective training with a delayed onset. Both training groups will initially comprise 80 subjects[4].

[5] Please note that we decided to split the follow-up testing phase at a later stage. One half of the participants were tested 4.5 months and the other half 10 months after the last training session.

In addition, we carry out the same experimental measurements with two retest control groups (which do not receive any training). Thus, we are able to control for repeated measurements and temporal dynamics for changes in the measurement results. The first control group comprises 30 subjects and is measured in approximately the same intervals as the training group at the beginning of the study. The second control group comprises up to 60 subjects and is only measured after the two training cohorts (see Figure 3). The reason for the separation into two control groups is the limited scanner capacity. On the other hand, it also offers the possibility of controlling for seasonal fluctuations in the results (e.g., mood). All participants will be invited for a follow-up testing phase approximately 4.5[5] months after the last training, which is used to estimate the stability of the changes. Figure 3 shows an overview of the testing plan of the training and control groups. The exact scanner schedule can be found in the appendix.

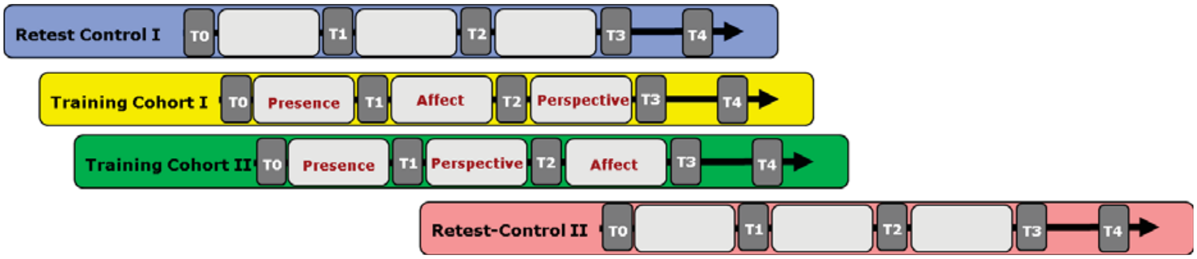

Figure 3: Overview on the study design with training and control cohorts.

In addition, we have another training control group (active control group, *N* = 80[4]). They first have a T0 baseline testing phase, before they start their training with a retreat and then train the Affect Module for 13-weeks (8 weeks + 5 weeks of deepening / repetition). From week 8 onwards, the group has yet another testing phase (T1). On a voluntary basis, the subjects can participate in a follow-up testing phase (T2) after 4.5 months[5]. The measurement paradigms correspond to the other two training groups. The training effects of this group can be compared with the results at T0 and T1 of the other two training groups (i.e. before and after the Presence Module, respectively).

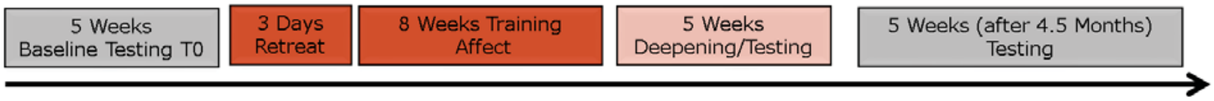

Figure 4: Overview on the active control group (*N*=80)[4], which only participates in the training of the Affect Module and will be tested two times (+ voluntary follow-up)[5].

3.4.2 Training Effort for Participants

At the beginning of each training phase (a total of 3 times), the participants have a 3-day intensive retreat (always Friday-Sunday), during which they are taught within 16 teaching hours the basics for the upcoming training module and the core exercises of the training module (a schedule for the retreat days is attached in the appendix). These retreats will be held with

overnight stays in retreat centers. All travel and accommodation expenses of the participants are covered by the funding agency or Max Planck Society.

Afterwards, the participants meet once a week for a 2-hour lesson, during which they are instructed by teachers. The course will be held in Berlin for participants from Berlin and in Leipzig for participants from Leipzig. We will offer two course dates per week. These dates also last for the 5-week testing phases and serve to deepen and maintain the learned content. On the remaining days of the week, the subjects are asked to perform the core exercises independently at home (daily about 30 minutes), supported by a web platform (see Chapter 3.7.2). An overview schedule for the training courses is attached separately.

A total of 80[4] subjects will be recruited per training cohort and active control cohort, with one half ideally being recruited from Berlin and the other half from Leipzig. The approximately 40 subjects per city are then divided into 2 groups and taught at two possible appointments per week. Therefore, about 20 people will be present in each weekly training group. Since the subjects should participate in the core phase of the training modules (week 1 to 8), it would be advisable if they could schedule their private holidays for instance during the testing phases. The testing can be adjusted flexibly around the absence times of participants.

**Kind of Training**	**Training Effort per Module**
Retreat	3 days per Training Module with ca. 16 hours of training
Weekly Session	2 hours, once a week, for 13 weeks
Practice at Home	up to 30 min daily

3.5 Description of the Training Protocol

For each of the three training modules, we have selected two central exercises (core exercises). **Note: These core exercises have already been described and approved in a previous ethics application (Leipzig: # 225-12-ff-02072012; HU Berlin: # 2012-26F).** Further exercises in the retreats and the weekly sessions serve to consolidate and deepen the contents in order to cultivate those qualities and dispositions in everyday life. The maximum 30-minute daily exercise time for the participants at home consists of performing one of the core exercises or in some cases also short variants of both core exercises.

The participants are always informed about the objectives and the theoretical background of the exercises and have the opportunity to discuss them with their teachers during the weekly sessions.

3.5.1 Content of the Presence Module

Description of the Presence Module:

In this module the participants learn to turn to the reality of the present moment. This includes the ability to direct attention, that is, the awareness of the content of consciousness and its purposeful orientation, to a present object. It also requires interoceptive awareness, that is, to

access internal processes and thus accessing one's own emotions and empathy for instance (e.g., Niedenthal, 2007, Pollatos et al., 2007, Singer & Lamm, 2009).

Description of the Core Exercises:

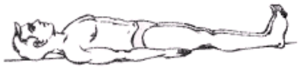

The first core exercise is the **Body Scan** (e.g., Kabat-Zinn, 2006). Participants lie or sit and guide their attention slowly through the body, trying to perceive body sensations consciously and without evaluation. As soon as they notice that attention has wandered, it is directed back to the body. Thus, the whole body is explored, and the attention is brought back to the sensations again and again, which leads to a stabilization of attention and an increase in clarity regarding the body sensations perceived (interoceptive awareness). This exercise takes about 20-30 minutes and is practiced by the subjects at home.

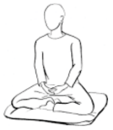

The second core exercise is an **attention meditation with breathing as an object.** The sensations that are associated with the flow of the air are continuously monitored. Here, too, the attention is always drawn back to the breath, when attention is distracted by thoughts or images. In the course of the practice, various aspects of attention can be strengthened by fine modulations of the exercise (for practical examples, see Wallace, 2006) (see also Semple, 2010; Tang et al., 2007; Tang & Posner, 2009 regarding effectiveness). This exercise takes about 10-30 minutes and is also practiced by the subjects at home.

[Further content of this Chapter has been omitted as the training protocol is in the process of being licensed with the Max Planck Society]

3.5.2 Content of the Perspective Module

Description of the Perspective Module:
In this module participants cultivate the cognitive aspects of compassion. This means that they learn to take different perspectives on their thoughts, on the self, and on others. In the case of thoughts, the cultivated perspective is an observation without identification. Thoughts are understood as internal events and their contents reflect a possible but not the only possible description of the world. A similar perspective is cultivated on self aspects. Here, too, subjects try to recognize the inner attitude or role with which they behave toward the world and toward themselves as a possible but not the only possible construction of oneself. The observation of change and transience of thoughts and self-concepts plays a decisive role in the gaining of insights. Finally, participants learn to take on the perspectives of others, i.e. to understand other people with their motives, thoughts, and values (Theory of Mind / mentalizing).

Description of the Core Exercises:

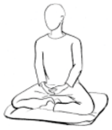

The first core exercise of this training module is a sitting meditation on the **observation of thoughts**. Thoughts are understood as a stream of mental events in the whole organismic structure. The connections of thoughts with emotions, physical states, and tendencies of action are observed. Thus, the gap between the emergence of a thought and the habitual reaction is broken ("de-fusion"), which leads to more

flexible emotional reactions and behavior. A variation of this exercise is also used in "stress management through mindfulness". The positive effects of "de-fusion" have been demonstrated in studies on "Acceptance and Commitment Therapy" (e.g., lower subjective credibility of stressful, anxiety-inducing, or depressive thoughts and associated symptom improvement in a variety of psychological problems (Bach & Hayes, 2002, Luoma & Hayes, 2002)). The exercise lasts 20 to 30 minutes and will be practiced by the subjects at home.

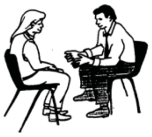

The second core exercise, on **perspective taking on self and others**, is based on the idea of a person as an assembly of inner roles or self-aspects, in the sense of connected patterns of thoughts, feelings, and behavioral patterns (see for example, Holmes, Mones & Schwartz, 2007). Some of these self-aspects, such as for instance the “inner judge, the optimist, the machinist, and the helpless,” are first identified under the guidance of an experienced teacher (e.g., a Gestalt Therapist). The actual exercise is a so-called dyadic exercise (partner exercise), in which two partners describe to each other a situation of their life, taking the perspective of one of their inner parts.

Dyadic exercises have been used in various compassion- and mindfulness-based training programs to support the outcome of the intervention (Mindfulness-Based Relationship Enhancement; Carson et al., 2004, e.g., Compassion Fatigue Specialist Training, Gentry et al., 2004). In particular, if interpersonal skills such as compassion are to be trained, the addition of interactive exercises together with meditation-based training seems sensible. The exercise we use is based on methods that are used within the paradigms of the so-called Inner Family System (Holmes, 2010; Mones & Schwartz, 2007) and transactional analysis (Berne, 2006). One effect of the exercise could be the increase of self-complexity and self-flexibility in the sense of a conscious perception on the variability of the self over different situations and their changeability in case of fixed non-adaptive behavioral patterns. Self-complexity acts as a buffer against stress-related illnesses and depression (Linville, 1985, 1987a, Rafaeli & Hiller, 2010), and attenuates affective responses to defeats and resulting withdrawal reactions (Dixon & Baumeister, 1991).

[Further content of this Chapter has been omitted as the training protocol is in the process of being licensed with the Max Planck Society]

3.5.3 Content of the Affect Module

Description of the Affect Module:
In this module the participants devote themselves to the cultivation of motivational-emotional aspects of compassion. They seek access to feelings of caring, affection, warmth, and love in order to approach themselves and others ("Opening the heart"). They also learn to approach difficult emotions in an accepting way, which is the basis for working with these emotions and their triggers as well as for "opening of the heart". Prosocial motivation as an access to and as a result of feelings of warmth and affection is discussed.

<u>Description of the Core Exercises:</u>

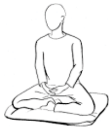

The first core exercise in this training is a so-called **Metta meditation** (Sanskrit for "loving kindness, friendship or benevolence“; see e.g., Salzberg, 1995). In the secular terminology of the training, this meditation will be called "heart meditation." This meditation is about orienting the mind in a way that allows one to affectionately and compassionately turn towards ourselves and other people. The meditating person visualizes different people (themselves, a friend, a neutral, and a "difficult" person) and sends them good wishes (e.g., happiness, health, and safety).

Metta meditation has been used in various interventions to improve psycho-social well-being (Fredrickson et al., 2008; Hofmann et al., 2011; Klimecki et al., 2012; Pace et al., 2009). The effects include, among other things, an increased occurrence of positive and benevolent feelings towards oneself and others (Fredrickson et al., 2008; Hutcherson et al., 2008), subjective improvement of physical symptoms such as back pain (Carson et al., 2005), a lower physiological and subjective stress response to stressful situations (Pace et al., 2009), increased sensual experiences, the improvement of relationships, and perceived enhancement of social support (Fredrickson et al., 2008). The exercise is performed over about 20-30 minutes and will also be practiced at home.

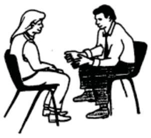

The second core exercise of this module is a dyadic exercise. This exercise is on **dealing with difficult emotions and cultivating gratitude**. Participants will tell each other about situations that were difficult for them in the last day and situations for which they are grateful.

The retrospective narration of difficult moments helps to better deal with them (see Frattaroli, 2006, Rimé, 2007 for a review and a meta-analysis). The effects mainly relate to improvements in mental health (e.g., reduced anger and stress) and perceived physical health (Frattaroli, 2006). The reasons that lead to the effectiveness of the exercise are manifold. First, awareness of emotions and their understanding in the context of the events is an important prerequisite for the regulation of emotions (King, 2002, Southam-Gerow & Kendall, 2002). On the other hand, talking about an event in a social support situation can lead to a more positive re-evaluation of the recollected episode (Klein & Boals, 2001; Pasupathi, 2003), whereby mechanisms such as extinction and conditioning within a positive context also play a role. Further discussion on the underlying mechanisms of communicating negative emotions can be found in Frattaroli (2006) and Rimé (2007).

In the second part of the exercise, subjects systematically cultivate feelings of gratitude. Such interventions are described, for example, by Emmons & McCullough (2003) and Lyubomirsky, Sheldon, & Schkade (2005). A focus on circumstances for which we are grateful helps to makes us aware of resources and opportunities and activates positive feelings. After several weeks of gratitude practices, subjects reported that they were more satisfied with their lives and reported a lower rate of physical symptoms, as well as more athletic activity and better sleep quality (Emmons & McCullough, 2003; Lyubomirsky et al., 2005). Subjects also reported increased feelings of social attachment. Bartlett & DeSteno (2006) also showed that experimentally

induced gratitude leads to an increase in prosocial behavior (see also Emmons & McCullough, 2003).

[Further content of this Chapter has been omitted as the training protocol is in the process of being licensed with the Max Planck Society]

3.6 Teachers

The program is led by a total of eighteen teachers[6]. All have years of experience with meditation and instructing meditation in groups. Many are also psychotherapists according to the psychotherapeutic law and also have experience with the guidance of groups and instruction of partner exercises. An overview of our teaching team is available on our Internet platform (www.resource-project.org). During the weekly teaching session, participants will always be instructed by a team of two teachers. The teachers are available during these sessions as a contact for questions by group participants. They are also available for half an hour after the session in order to answer individual questions from participants. Once a month, each subject has a compulsory individual consultation with a teacher to discuss the training process. For very urgent questions and problems, one teacher (regularly changing) is available around the clock by phone.

3.7 Technical Support

3.7.1 Smartphones

A smartphone is provided for all subjects for the duration of the study. On this smartphone a software was installed which was programmed for the purpose of the study to help subjects perform the contemplative core meditation and dyadic exercises and answer brief questions regarding their situation and states (see Chapter 4.5.3). No personal data is stored on these smartphones. The data is only stored anonymously and via the subject identification number and only the answers relevant to the study are recorded. If a network connection is available, the data is transmitted encrypted to our Max Planck server.

3.7.2 Online Platform

An online platform has been developed for this study that serves various project purposes (www.resource-project.org). This Internet presence is hosted on an internal server of the Max Planck Institute for Human Cognitive and Brain Sciences.

This website will be used as follows:

---

[6] Originally, we had planned to recruit 18 teachers, however, in the end we had a total number of 17 teachers that instructed the participants in the respective modules.

1. Recruitment: People interested in participating in the study can apply for the project via the Internet website www.resource-project.org. The exact recruitment steps are described in Chapter 5.2. Each student must submit his / her consent statement regarding the storage and use of his or her personal data before completing the recruitment process.

   Statement on the recruitment web page: I hereby confirm that the information provided in this form is correct and complete. I am aware that the data collected during the experiments are used anonymously for evaluations and scientific publications of the institute. I agree that the Institute may store my personal data (e.g., name, address, telephone number, etc.) in digital form and use it for scientific purposes. I can revoke this consent at any time by writing an e-mail to the database group.
   Button: "Yes, I agree."

2. In order to support their daily practice, the students are given access to an online platform specially developed for the project.
   Each participant is given personalized login data, through which he/she enters the password-protected practice and testing area. Here, the subjects have the possibility to perform audio- and video-supported meditation exercises, so-called "guided meditations." In addition, the participants perform exercises on this platform, where they are connected to another participant ("dyadic exercise") by means of Internet telephony. The exact description of the exercise can be found in Chapter 3.5. The subjects will answer psychological questions before and after the meditations, as well as before, during, and after the dyads.
   Independent of the exercises, the subjects will in addition answer questionnaires by the help of this platform.
   All data collected via this platform is stored in an anonymous form and pseudo-anonymized by means of a code, so that the results of the questionnaires, exercises, and computer tests can be combined.
   See Chapter 7 for further information regarding the use of subject data, data collection, data processing, and data protection.

4 Description of Paradigms and Methods of Investigation

A summary of the time required for the testing is given at the end of this chapter under section 4.8.

Due to the scope of this application, we will only be able to describe roughly the most important differential hypotheses of the individual paradigms. For all paradigms, however, there are a number of further differentiated hypotheses about the expected changes of the subjects in the respective measurements over the course of the training, i.e. in comparison to T0 and the individual different training modules (Presence, Affect, and Perspective). Furthermore, there are hypotheses for the comparison of retest control participants and participants of the respective training cohorts. The active control group can also be compared with the first part of the training cohorts (Presence Module). Furthermore, we will examine the relationship between individual differences to T0 before the training and individual differences in the respective learning processes during the different training modules. Hypotheses, which compare the individual dimensions with each other to T0 as well as in the course of the change, are not included in this application (for example, there are hypotheses for the interrelations of the subjective well-being and the results of the cortisol daily profiles). However, most important differential hypotheses are listed.

4.1 Imaging by means of Magnetic Resonance Tomography

[This part is omitted as details pertain to manuscripts in progress]

4.1.1 Structural Imaging

[This part is omitted as details pertain to manuscripts in progress]

4.1.2 Functional Imaging

[This part is omitted as details pertain to manuscripts in progress]

4.2 Paradigms in Virtual Scenarios

[This part is omitted as details pertain to manuscripts in progress]

4.3 Behavioral Computer Experiments

[This part is omitted as details pertain to manuscripts in progress]

4.3.1 Perception Sensitivity and Meta-Cognition

[This part is omitted as details pertain to manuscripts in progress]

4.3.2 Inhibition and Alerting (Stopp-Signal-Reaction-Time-Task)

[This part is omitted as details pertain to manuscripts in progress]

4.3.3 Working Memory and Meta-Cognition

[This part is omitted as details pertain to manuscripts in progress]

4.3.4 Attentional Blink Paradigm
[This part is omitted as details pertain to manuscripts in progress]

4.3.5 Task-switching Paradigm
[This part is omitted as details pertain to manuscripts in progress]

4.3.6 Emotional Granularity Task
[This part is omitted as details pertain to manuscripts in progress]

4.3.7 Socio-affective Video Task (SoVT)
[This part is omitted as details pertain to manuscripts in progress]

4.3.8 Samson’s Test
[This part is omitted as details pertain to manuscripts in progress]

4.3.9 Economic Games
[This part is omitted as details pertain to manuscripts in progress]

4.3.10 Self-complexity Task
[This part is omitted as details pertain to manuscripts in progress]

4.3.11 Self-reference Task
[This part is omitted as details pertain to manuscripts in progress]

4.3.12 Task for the Assessment of „Mind-Wandering“
[This part is omitted as details pertain to manuscripts in progress]

4.3.13 Donation Task
[This part is omitted as details pertain to manuscripts in progress]

4.4 Questionnaires
[This part is omitted as details pertain to manuscripts in progress]

4.5 Subjective Methods

4.5.1 Weekly Experience Sampling via the Internet platform
[This part is omitted as details pertain to manuscripts in progress]

4.5.2 Daily questions about the exercises via the Internet platform or the smartphone

Background:
These short meditation-related control questions are carried out to assess the immediate psychological effects after a meditation or dyadic exercise.

Methods:
The participants should answer short questions about their emotions, attention, and thoughts directly before and after their meditations and exercises. This is provided by our platform or our smartphones, as the subjects should log in at home at the beginning of their exercise on the

platform or the smartphone. The dyadic exercises are to be carried out via the platform or cell phone apps and also the meditations can be conducted at home with the help of audio files.
Before and after the dyadic exercises, the subjects also answer questions about the perception of the partner. After all meditations or exercises, the participants evaluate the ease and convenience of the task just completed. An overview of the questions can be found in the appendix[7].

Hypotheses:
We assume that the specific questions about meditation practice and the dyadic exercises can be applied to their differentiated and specific effects. We assume that the subjects, especially after the Presence training (compared to the other blocks), report that they are more aware of themselves after the mediation and are more aware of their body. Furthermore, we assume that the subjects are increasingly thinking about themselves and others after the meditation in the Perspective training (compared to the other modules). The Affect Module, compared to the other modules, causes the subjects to have more positive emotions and less negative emotions after mediation. Throughout the course of the training the participants should report less distracting thoughts the better they perform the exercises. After the perspective and affect training, an increased closeness to the dyadic partner should be detected. During the course of the perspective training, the subjects will report that they accept the inner parts more easily and comfortably. During the course of the Affect training, the subjects will report more positive emotions after the dyadic exercises.

Timeframe:
The questions for this experiment consist of short sentences and the subjects can quickly select an answer. The questions can be answered after login on the platform and appear before the beginning and after the end of an exercise. These take about 4 minutes each.

4.5.3 Collection of Experience-sampling Data with Smartphones

[This part is omitted as details pertain to manuscripts in progress]

4.5.4 Observer Reports

[This part is omitted as details pertain to manuscripts in progress]

4.6 Stress Physiology

4.6.1 Diurnal Cortisol Profiles

[This part is omitted as details pertain to manuscripts in progress]

4.6.2 Trier Social Stress Test (TSST)

[This part is omitted as details pertain to manuscripts in progress]

[7] The exact questions of the questions asked before and after the dyadic exercises can be found in the appendix.

4.7 Assessment of autonomic Functions

[This part is omitted as details pertain to manuscripts in progress]

4.7.1 Vagal Tone

[This part is omitted as details pertain to manuscripts in progress]

4.7.2 Autoregulation of the state of excitation

[This part is omitted as details pertain to manuscripts in progress]

4.7.3 Heart Beat Perception

[This part is omitted as details pertain to manuscripts in progress]

4.7.4 Measurement of autonomous functions and movement behavior during meditation

[This part is omitted as details pertain to manuscripts in progress]

4.8 Qualitative Interviews

[This part is omitted as details pertain to manuscripts in progress]

4.8.1 Voluntary Interviews on the Subjective Experiences of the Training

[This part is omitted as details pertain to manuscripts in progress]

4.8.2 Voluntary Elicitation Interviews

[This part is omitted as details pertain to manuscripts in progress]

4.9 Summary of the Time Required for the Measurements

4.9.1 Magnetic Resonance Imaging (MRI) and Virtual Reality (VR) Scenarios

The MRI measurements (see Chapter 4.1) take place in each testing phase, so to say before the training (T0), after the training modules, and again 4.5[3] months later (T1–T4).

The participants from will have to travel to Leipzig for one day, since there is no MRI scanner available in Berlin. The pure MRI scan time of ca. 140 minutes (see table below) is divided into two sessions (about 70 minutes each) during the day. This allows us to measure up to 3 participants per day. Between the two MRI sessions, subjects have a long scanning break of at least 3 hours, which also allows us to carry out the experiments in a virtual environment (see Figure 4).

The VR experiments, including instructions and preparation of equipment take a maximum of one hour, but the paradigms alone last a maximum of 40 min (see Chapter 4.2). There is no VR laboratory available in Berlin. We perform the MRI and VR measurements on this one day so that participants from Berlin do not have to travel twice or have to stay overnight. This reduces the effort of the subjects. All other paradigms can be carried out at home and in the behavioral labs in Berlin.

MRI-paradigms	Scanning time per testing phase (min)
Structural measurements	20
Calibration	7
Emotion generation/regulation	25
Emotional responsiveness	15
Pain perception	23
Attention	15
Theory of Mind / Empathy	35
**Total**	**ca. 140 min**
**All VR paradigms**	**ca. 60 min**

07:30			
08:00	Person 1		
08:30	MRI Part 1		
09:00			
09:30	Break	Person 2	
10:00		MRI Part 1	
10:30	VR		
11:00		Break	Person 3
11:30	Break		MRI Part 1
12:00		VR	
12:30			Break
13:00	Person 1	Break	
13:30	MRI Part 2		VR
14:00			
14:30		Person 2	Break
15:00		MRI Part 2	
15:30			
16:00			Person 3
16:30			MRI Part 2
17:00			
17:30			

Figure 4. Scanner and VR-plan for one day with 3 subjects. Each subject has 2 scan times and a VR session with pauses between sessions.

4.9.2 Behavioral computer paradigms

The behavioral computer experiments take ca. 5 hours per testing phase. Since multi-computer labs (20 computers each) are available in Berlin and Leipzig, several participants can simultaneously take part in these experiments. These experiments can, for example, be carried out before and after the weekly training sessions (e.g., 30-60 minutes each), or at other times

when the subjects have other appointments. The experiments will always be spread over several session dates.

Computer paradigms	Time per testing phase
Perceptual Sensitivity & Meta Cognition	20
Inhibition & Alerting SSRT	10
Working Memory & Meta-Cognition	20
Attentional Blink Paradigm	30
Task-switching Paradigm	30
SoVT (+ measure autonomous functions)	See below
Task on Emotional Granularity	20
Samson’s Test	25
All economic games	45
Self-complexity task	15
Self-reference task	15 (only 2 x per subject)
Mind-wandering (CRT/WM)	24
Donation task	10
**TOTAL**	**279 (ca. 5 hours, distributed over 6 sessions)**

4.9.3 Questionnaires

The completion of questionnaires (see Chapter 4.5) for each testing phase (T0–T4) is of variable duration. The questionnaires can be completed at home via the Internet platform. The subjects can decide for themselves when to complete the questionnaires during the testing phase. This can also be done in several sessions within one phase. The maximum total filling time is about 3 hours.

4.9.4 Subjective Methods

The assessment of the acute and subjective cognitive and emotional states of the subjects takes place not only in the testing phases, but also during the 8-week training phases.

- Once a week at home: subjects answer questions via the platform. This does not take more than 4 minutes
- Daily monitoring questions during the exercises performed at home via the platform. 4 minutes before the core exercise and 4 minutes after the core exercise
- Experience-sampling using the smartphones combined with the diurnal cortisol profiles. This is done on 2 consecutive days. The saliva samples are taken with salivettes approximately 8 times a day. Meanwhile, the Experience-sampling questions can be answered. The experience sampling also takes place approximately 8 times a day and takes a maximum of 2–4 minutes each. This is done once per training module and at T0 and T4.
- Observers' reports do not represent a time effort for the actual training subjects.

4.9.5 Stress Physiology

The TSST is conducted only once per subject. The subjects are distributed over the training modules (between-subject design). Since the TSST is an acute stress paradigm, it will be carried out on a separate date. The test takes ca. 110 minutes.

4.9.6 Measurement of Autonomic Functions

Some autonomic data collection takes place in parallel to some other experiments (VR, MRT, SoVT, TSST). The assessment of the autonomic functions (vagal tone activity, 4.7.1, autoregulation of the state of excitement, 4.7.2, and heartbeat perception, 4.7.3) can be executed with up to 4 subjects at the same time before or after the training or on a separate day, if the subjects has other appointments. The measurements take ca. 5, 8, and 4 minutes without instructions/sensor attachment. This is usually combined with the implementation of the SoVT (15 minutes), since physiological data are also collected during this time.

**Physiological Data Assessement**	**Duration**
Vagal activity	5 minutes
Autoregulation	8 minutes
Hearbeat perception	4 minutes
SoVT	15 minutes
Instructions	ca. 8 minutes
**Total**	**40 minutes**

4.9.7 Qualitative Interviews

The elicitation interviews last ca. one hour each. The length of the interviews with regard to the subjective experience of the training course can vary. Both interviews would only be conducted on a completely voluntary basis and are only planned with a few volunteers.

5 Introduction from Addendum I

At this point we add the content from the first addendum.

As already described in the main application #376-12-05112012, the main goal of the "ReSource" study is a scientific evaluation of an intervention for the mental training of socio-affective and cognitive abilities trained by healthy volunteers over several months.

In this already approved main application we have described in detail how we want to examine the training potential of these socio-emotional and cognitive abilities and their effect on the subjective well-being, brain-plasticity and behavior. The methods mentioned in the main application have hitherto included investigations of behavioral changes, experience-sampling methods as well as functional and structural paradigms by means of magnetic resonance

tomography, and psycho-physiological measurements (stress physiology, measurements of the autonomous nervous system) and experiments in virtual environments.

In this addendum, we describe additional biological markers that we would like to incorporate into the "ReSource" Project. They would allow the investigation of the influence of one-year mental training on health-relevant aspects (e.g., by obtaining data from some cytokines, immunoglobulin A, cortisol, oxytocin, etc.). Furthermore, we will also look at the correlation between certain gene polymorphisms of the subjects and inter-individual differences in their learning ability during the different training modules.

In particular, we would like to record the oxytocin levels of our subjects before and after a stressor, as well as before and after a certain form of mental training, as we assume that the oxytocin release is enhanced particularly during the course of the affect training module.

A further stress-sensitive measure would be cortisol assessment in the hair of subjects, which represents a new prospective possibility of recording long-term concentrations of cortisol, which correlates with the stress experienced by the subjects in recent months. We assume that the training results in stress reduction in our subjects and this is reflected in the hair samples.

Finally, we would like to assess the telomere length of the subjects at several time points. Since the training is aimed at an improvement of stress resistance and general well-being, we may determine this by a reduced shortening of the telomere length.

6 Biological Analyses

6.1 Summary of planned blood samples

[This part is omitted as details pertain to manuscripts in progress]

6.2 Genetic analyses of gene variants / polymorphisms

[This part is omitted as details pertain to manuscripts in progress]

6.3 Cytokine analyses from blood samples (during the training process, pre-post acute stress)

[This part is omitted as details pertain to manuscripts in progress]

6.4 Immunoglobulin A Analysis from saliva samples (pre-post acute stress)

[This part is omitted as details pertain to manuscripts in progress]

6.5 Oxytocin analyses from blood samples (pre-post acute stress, pre-post of a mental training session)

[This part is omitted as details pertain to manuscripts in progress]

6.6 Cortisol analyses from hair samples (during training)

[This part is omitted as details pertain to manuscripts in progress]

6.7 Telomere length (during training)

[This part is omitted as details pertain to manuscripts in progress]

6.8 Number of cell types in the blood

[This part is omitted as details pertain to manuscripts in progress]

7 Number of subjects, recruitment, exclusion criteria, payment, terms of cancelling participation

7.1 Number of Subjects

Our retest control group, which is measured at approximately the same time as our training cohort, consists of a maximum of 30 participants. This cohort does not receive any training and is only measured several times at the corresponding time intervals in order to control for retest and seasonal effects.

We have two training cohorts, each with a maximum of 80 subjects[4]. One half should be recruited from Berlin and the other half from Leipzig. The approximately 40 subjects are then divided into 2 groups and taught at two possible dates per week. Therefore, about 20 people are present in each weekly training session. Since the subjects are to participate in the training blocks of the core training phase (week 1–8), they are given the opportunity to plan their holidays in the 5-week testing blocks. We will be flexible in rescheduling some of the testing for the holidays of the subjects.

In order to obtain comparable group sizes, we plan to test a retest control cohort with 60 test participants, which will also be tested at similar regular time intervals but does not receive any training (see also Chapter 3.4.1). In addition, we have another training control cohort (active control group, N = 80). This cohort runs through a shortened training (only the Affect Module) and has 2–3 testing time points (i.e. T0 baseline testing, 1 Affect Module retreat, 13 weeks Affect Module training, T1 testing, voluntary T2 follow-up testing). The testing paradigms correspond to those of the other two training cohorts[2].

**Cohort**	**Number of Subjects**
Early retest control cohort	30
First training cohort	80
Second training cohort	80
Late retest control cohort	60
Active control cohort	80
**Total**	**330**[4]

7.2 Recruitment process

Healthy volunteers between the ages of 20 and 55 are recruited via advertisements and flyers.

a) During the recruitment process, the potential subjects are referred to our Internet platform. All potential volunteers first fill out an online contact form and a declaration of consent (see Chapter 3.7.2) and are asked questions regarding the exclusion criteria. This will take about 20 minutes.
b) In a second step, the subjects who have fulfilled all the prerequisites beforehand receive a login for the website. Then they are asked to complete the following questionnaires online: 1) Toronto-Alexithymia-Scale–20 (Kupfer et al., 2001); 2) Major Depression Inventory (MDI) (Bech et al., 2001); 3) State-Trait Anxiety Inventory (Spielberger et al., 1983); 4) SKID-I/II screening-questionnaire. This takes about 15 minutes. If the subjects do not show any clinical abnormalities after completion of these questionnaires, they are randomly divided into training or control cohorts[8].
c) The two groups are invited to different information events, which are obligatory but are offered several times. During this event, each subject receives an exact oral explanation about the purpose and course of the planned study. If the subjects are still interested after participating in such an event and after a personal telephone call, they will be divided into the respective subgroups by means of further questionnaires according to personality traits, IQ, gender, education, health status, and age.
d) The last recruitment step is a personal SKID I/II interview. These interviews are conducted by trained psychological psychotherapists in education (PiA), with 1600h psychiatry / psychosomatic experience as well as training and experience in the SKID procedure.

The subjects are explained the MRI procedure and risks by a doctor at least 24 hours before the MRI experiment. The subjects are told that, in the context of scientifically based studies with magnetic resonance imaging (MRI), unexpected pathological findings (so-called "random findings") are rarely discovered. In the case of a random finding, the subjects are informed verbally in detail about the random findings by a doctor. In addition, a proposal for the further neuroradiological-diagnostic clarification of the random finding is handed to them.

The institute provides a medical service during the testing periods. In case of any incidents, all possibilities of emergency care are given by the nearby University Hospital Leipzig.

7.3 Exclusion Criteria

These exclusion criteria are collected online via our Internet platform (after clarification of consent) (see 3.7.2) (and clarified once again by telephone):

---

[8] Please note that participants were only divided into training or control cohorts after step c had been completed.

- Not between the ages of 22 and 55
- Weight over 130 kg (no normal BMI)
- Not available on retreat dates or information events
- Not MRI compatible
- Left handedness[9]
- No computer, no internet connection
- Not German-speckig
- Reading and spelling impairment, color vision impairment
- Limitations in hearing, e.g., tinnitus
- Particularly sensitive to noise
- Diseases of the movement apparatus; no walking ability
- Involuntary motor disorders
- Heart pacemaker
- Carrying an insulin or medication pump
- Wearing a shunt (brain drainage)
- Metal objects after operations, metal objects / metal chips in the body
- Artificial heart valves
- Internal implants
- Joint prosthesis
- Permanent make-up or tattoos
- Not able to lie still for 2 hours
- Claustrophobia
- Neurological diseases, neuropathic diseases
- Epilepsy or cerebral convulsions
- Head injuries
- Cardiac arrhythmia
- Other heart diseases (vessels, heart valves)
- Vascular disease; arterial blood flow disturbances
- Respiratory tract diseases (e.g., asthma, tuberculosis, chronic bronchitis)
- Surgery within the last two months (including eyes/ears)
- Pregnancy and lactation or definitely-planned pregnancy in the next few months
- Renal function disorders
- Diabetes
- Suicide endangered or suicidal thoughts
- Chronic pain
- Migraine
- Cognitive deficits
- Circulatory disorders (e.g., As PAD, Raynaud's syndrome), venous diseases, skin lesions
- Allergies to adhesive tapes or patches

[9] Note that left handed participants were allowed to participate in the study if they indicated that they could use a computer mouse with their right hand.

- Psychology students; Psychotherapy experience
- In psychological treatment
- Psychological disorders
- Regular intake of cortisone, steroids, corticosteroids (e.g. asthma)
- Shift service workers
- TMJ disorders or other problems with chewing / swallowing
- Alcohol and drug abuse
- Known psychological disorders (through SKID interviews) that are less than 2 years old
- Current or planned intake of medications that affect the CNS (e.g., opiates, psychotropic drugs)
- very regular experience and practice with meditation or yoga
- very regular religious activity (e.g., intense prayers)
- Chronic diseases (e.g., asthma, diabetes mellitus, etc.) that require permanent medication
- Intake of endocrine-affecting or psychoactive drugs

7.4 Payment and other benefits for the subjects

The participants receive a financial compensation of 7 Euros/hour for all behavioral experiments, the TSST[10], the psychophysiological measurements, the experience-sampling and the questionnaires. For MRI experiments and VR experiments a compensation of 8 Euros/hour is paid.

The voluntary "observers" of the participants are also compensated with 7 Euros/hour.

At the end of the first training module, the subjects receive an additional one-off incentive fee of 100 Euros. At the end of the second module, they receive 200 Euros, and after the third module 300 Euros. The control subjects received these amounts after the corresponding intervals as well.

The mental training is free and is carried out by experienced teachers. The retreat costs, travel expenses, and teaching costs are covered by the funding agency or the Max Planck Society.

Furthermore, for each of the economic games (see Chapter 4.3.11), a certain amount of money (e.g., between 2 and 10 Euro) is provided, which the participant can use during the game. From this sum, the subject can lose money to others or gain money. The final amount can be retained after the end of the game. This means that the subjects can never leave the experiment with less money than they had before. In addition to the amount that can be gained in the games, the usual fixed money (7 Euro/hour) is given to each participant.

[10] Please note that we also paid 8 Euros/hour for the TSST experiment.

All the exercises used in the training have scientifically-documented, positive effects on psychological and physical well-being. This also applies in part to short interventions (see for example Tang et al., 2007). The subjects learn mental techniques, which they can continue to use after the end of the study in order to achieve sustainable improvements in their general well-being or in individual aspects of mental health.

7.5 Terms of cancelling the study participation

Rejection or termination of participation is possible at any time and without giving reasons at the request of the subject. There are no disadvantages to the subjects. A text informing the subjects of their rights regarding termination or rejection of their participation can be found in the consent forms.

8 Risks for the subjects, planned arrangements regarding risk reduction, subject insurance

8.1 Possible Risks and Complications; Dealing with Complications

Before the start of the study, subjects are invited to a detailed information session, during which they will be informed about possible risks and complications that may occur during the study. Throughout the study, the participants have the possibility to receive advice from one of the therapists and medication teachers on the spot and by phone. In addition, a refusal or termination of participation at any time and without giving reasons at the request of the subject is possible.

8.1.1 Risk of Training

There are in general only very few unpleasant side effects of meditation known (for a review see Dobkin et al., 2011). This applies at least to people without psychiatric conditions, which we ensure in our study through pre-screening and detailed psychiatric interviews.

Occasional and temporary side effects can be the appearance of unusual or intense body sensations and feelings (Perez-De-Albeniz & Holmes, 2000). These phenomena can be dealt with by the help of an experienced meditation teacher or psychologist. In very rare cases, intense emotional states (e.g. crying) can occur (Dobkin et al., 2011). However, these experiences are, as far as they are adequately supported by the teacher, experienced by the individual and the group as helpful and salutary (Allen et al., 2006).

The communication of negative emotional experiences in the context of the affect dyadic exercise can be experienced as initially irritating by some subjects. In the long run, however, the results suggest that the sharing of negative experiences in a protected, structured framework helps to re-evaluate experience, de-traumatize it, and develop skills for regulating emotions (Frubaroli, 2006, Lyubomirsky et al., 2006, Pasupathi , 2003; Rimé, 2007). All our teachers have extensive experience with the difficulties that may arise during and after meditation and partner exercises and know what measures are appropriate (e.g., relaxation, mediation modification, shorter meetings, etc.).

In the context of the perspective taking exercise, the discussion of the fact that the self is a constantly changing structure can initially have a new and confusing effect on some subjects and may in some cases trigger questions about the complexity of one's own personality. These questions are addressed by the accompanying therapists and teachers in order to support the training process.

8.1.2 Risks of Magnetic Resonance Tomography

8.1.3 Risk of Pain Paradigm

8.1.4 Deception During Economic Games

8.1.5 Virtual Reality Scenarios

8.1.6 Behavioral Experiments

8.1.7 Trier Social Stress Test

8.1.8 Blood Collection

8.2 Further Treatment after End of Study

Only healthy volunteers participate in the study. The data from the genetic analyses are not intended for medical diagnostics. There is therefore no further treatment after the end of the study. Complete or diagnostic DNA analyses will not occur. Blood analysis data will also not be used for diagnostic purposes.

8.3 Risk-Benefit Assessment

The risks for the subjects are extremely low. Our department has gained many years of experience with mental training, meditation, and compassion training and has only made positive experiences so far. The vast majority of subjects reported that the training was of great benefit to them personally and was fun. The unpleasant emotions and personally important questions that occur in very rare cases can be part of the process initiated by mental training, meditation, and dyadic exercises. However, our teaching staff has extensive experience with

this process and is able to accompany subjects adequately. Psychological care is given at all times (on the premises during the training sessions and via phone).
The mental training carried out during this study aims to improve the well-being of already healthy volunteers (see Chapter 3.3 for individual, scientifically-documented effects). Psychologically unstable persons are excluded from these studies, as these need psychotherapeutic support, which clearly must be distinguished from the training formats often used in research.
The research project will help to expand our understanding of socio-affective plasticity of experience and behavior through mental training. Considerable research is still necessary with regards to the ability to train socio-emotional abilities and associated structural brain and behavioral changes (see for example Kotsou et al., 2011 S. 828ff).

No health impairments are known regarding the planned biological methods (salivettes, hair samples, blood analyses). Through the use of these methods, there has been a considerable gain in knowledge in stress research over the last decades. For the participants, there is not much additional testing effort required since the blood samples, hair samples, and saliva samples can be coordinated without additional appointments at the institute. These findings can serve as a starting point for the development of secular, mental training programs to increase mental health. The trained trait, compassion, also plays an important role in improving social cohesion and peaceful, supportive coexistence. The exploration of the plasticity of compassion is thus a contribution to the clarification of urgent questions, which are decisive for the prevention and resolution of conflicts - from the family to the global political level.

8.4 Participant and Accident Insurance

The participants are subject to insurance cover for statutory claims under private law in the framework of the company liability insurance of the Max Planck Society (Basler Securitas Allgemeine Haftpflichtversicherung, Insurance No. 320027-3181262 DI, Policyholder: MPG).

We additionally insured participants for personal accident coverage (HDI Gerling Industrie Versicherung AG, Nr: 39-132504-03452 / 390). The insurers were informed about the additions from this addendum. It was confirmed that the subject insurance also exists with these supplements.

Insurance pledges:

- During the duration of the study, further medical treatment is applied only in agreement with the investigator (except in emergencies)

- Any deterioration in the state of health should be reported immediately to the investigator who assesses whether it is a health hazard that could have occurred as a result of the study.

- In the case of damage, the treating physician is authorized to provide the insurer with information on request.

A separate travel-related accident insurance (HDI Gerling Industrie Versicherung AG, No. 39-132504-03476) has been concluded.

9 Data Collection, Data Processing, Data Usage, and Data Protection

The personal and formal circumstances of the subjects (e.g., name, date of birth, telephone number, address, e-mail address) are used exclusively for the selection and contact for participation in scientific studies.
The results will only be shared with the consent of the subjects. The collected data are stored in an anonymous manner. The data is pseudo-anonymized by means of a code, so that the results of the questionnaires and tests can be combined. Apart from these, the data are only available to the researchers leading this study and, if necessary, to responsible authorities. These sites are subject to confidentiality. Subsequent publications represent summary values of the results of the investigation. Conclusions regarding individual persons are therefore not possible. After completion of the evaluation, the documents are archived according to legal requirements.
The laws of data protection (BDSG, BlnDSG, and ecclesiastical data protection laws) are respected for all collected data.
All hair samples are transferred to the laboratory of the Department of Biopsychology of the Dresden University of Technology, Prof. Clemens Kirschbaum for analysis. No genetic analyzes are carried out with the hair samples.
Only healthy subjects participate in the study. The data from the blood samples are not used for medical diagnostics. There is therefore no further treatment after the end of the study. Complete or diagnostic DNA analyses will not occur.

10 Declaration that the “Declaration of the World Medical Association of Helsinki” is known

We hereby declare that we are aware of the Declaration of the Helsinki Medical Association and that our project has been planned according to the guidelines of this declaration.

11 References

Administration, C. f. t. D. a. R. H. F. a. D. (1998). Guidance for the Submission of Premarket Notifications for Magnetic Resonance Diagnostic Devices.

AIP. (2005). American Institute of Physics.

Aleman, A., Swart, M., & van Rijn, S. (2008). Brain imaging, genetics and emotion. *Biol Psychol, 79*(1), 58-69. doi: 10.1016/j.biopsycho.2008.01.009

Allen, N., Blashki, G., Chambers, R., Ciechomski, L., Gullone, E., Hassed, C., . . . Meadows, G. (2006). Mindfulness-based psychotherapies: a review of conceptual foundations, empirical evidence and practical considerations. *Australian and New Zealand Journal of Psychiatry, 40*(4), 285-294. doi: 10.1111/j.1440-1614.2006.01794.x

Apkarian, A. V., Bushnell, M. C., Treede, R. D., & Zubieta, J. K. (2005). Human brain mechanisms of pain perception and regulation in health and disease. *Eur J Pain, 9*(4), 463-484. doi: 10.1016/j.ejpain.2004.11.001

Apperly, I. A., Warren, F., Andrews, B. J., Grant, J., & Todd, S. (2011). Developmental continuity in theory of mind: speed and accuracy of belief-desire reasoning in children and adults. *Child Dev, 82*(5), 1691-1703. doi: 10.1111/j.1467-8624.2011.01635.x

Aron, A., & Fraley, B. (1999). Relationship closeness as including other in the self: Cognitive underpinnings and measures. *Social Cognition, 17*, 140-160.

Association, R. P. (1991). International Non-Ionizing Radiation Committee of the International Radiation Protection Association. Protection of the patient undergoing a magnetic resonance examination. . *Health Phys, 61*, 923-928.

Bach, P., & Hayes, S. C. (2002). The use of acceptance and commitment therapy to prevent the rehospitalization of psychotic patients: A randomized controlled trial. *J Consult Clin Psychol, 70*(5), 1129-1139. doi: 10.1037/0022-006x.70.5.1129

Baddeley, A. (2003). Working memory: looking back and looking forward. *Nat Rev Neurosci, 4*(10), 829-839. doi: 10.1038/nrn1201

Baenziger, T., Scherer, K. R., Hall, J. A., & Rosenthal, R. (2011). Introducing the MiniPONS: A Short Multichannel Version of the Profile of Nonverbal Sensitivity (PONS). *Journal of Nonverbal Behavior, 35*(3), 189-204.

Baer, R. A. (2010). Self-Compassion as a Mechanism of Change in Mindfulness- and Acceptance-Based Treatments. In R. A. Baer (Ed.), *Assessing Mindfulness and Acceptance Processes in Clients: Illuminating the Theory and Practice of Change* (pp. 135-153). Oakland, CA: New Harbinger Publications.

Bakermans-Kranenburg, M. J., & van Ijzendoorn, M. H. (2008). Oxytocin receptor (OXTR) and serotonin transporter (5-HTT) genes associated with observed parenting. *Soc Cogn Affect Neurosci, 3*(2), 128-134. doi: 10.1093/scan/nsn004

Banaschewski, T., Becker, K., Scherag, S., Franke, B., & Coghill, D. (2010). Molecular genetics of attention-deficit/hyperactivity disorder: an overview. *Eur Child Adolesc Psychiatry, 19*(3), 237-257. doi: 10.1007/s00787-010-0090-z

Barnett, J. H., Jones, P. B., Robbins, T. W., & Muller, U. (2007). Effects of the catechol-O-methyltransferase Val158Met polymorphism on executive function: a meta-analysis of the Wisconsin Card Sort Test in schizophrenia and healthy controls. *Mol Psychiatry, 12*(5), 502-509. doi: 10.1038/sj.mp.4001973

Barrett, L. F. (2004). Feelings or words? Understanding the content in self-report ratings of experienced emotion. *J Pers Soc Psychol, 87*(2), 266-281. doi: 10.1037/0022-3514.87.2.266

Barrett, L. F. (2006). Solving the emotion paradox: categorization and the experience of emotion. *Pers Soc Psychol Rev, 10*(1), 20-46. doi: 10.1207/s15327957pspr1001_2

Barrett, L. F., & Niedenthal, P. M. (2004). Valence focus and the perception of facial affect. *Emotion, 4*(3), 266-274. doi: 10.1037/1528-3542.4.3.266

Barrett, L. F., Quigley, K. S., Bliss-Moreau, E., & Aronson, K. R. (2004). Interoceptive sensitivity and self-reports of emotional experience. *J Pers Soc Psychol, 87*(5), 684-697. doi: 10.1037/0022-3514.87.5.684

Bartlett, M. Y., & DeSteno, D. (2006). Gratitude and Prosocial Behavior. *Psychological Science, 17*(4), 319-325. doi: 10.1111/j.1467-9280.2006.01705.x

Batson, C., D. (1987). Prosocial motivation: is it ever truly altruistic? *Advances in Experimental Social Psychology* (Vol. 20). New York, NY: Academic Press.

Bech, P., Rasmussen, N. A., Olsen, L. R., Noerholm, V., & Abildgaard, W. (2001). The sensitivity and specificity of the Major Depression Inventory, using the Present State Examination as the index of diagnostic validity. *J Affect Disord, 66*(2-3), 159-164.

Bello, D., White-Traut, R., Schwertz, D., Pournajafi-Nazarloo, H., & Carter, C. S. (2008). An exploratory study of neurohormonal responses of healthy men to massage. *J Altern Complement Med, 14*(4), 387-394. doi: 10.1089/acm.2007.0660

Berg, J., Dickhaut, J., & McCabe, K. (1995). Trust reciprocity and social history. *Games and Economic Behavior, 10*, 122-142.

Berne, E. (2006). *Die Transaktions-Analyse in der Psychotherapie: Eine systematische Individual- und Sozialpsychiatrie*. Paderborn: Junfermann Verlag.

Bernhardt, B. C., & Singer, T. (2012). The neural basis of empathy. *Annu Rev Neurosci, 35*, 1-23. doi: 10.1146/annurev-neuro-062111-150536

Bevilacqua, L., & Goldman, D. (2011). Genetics of emotion. *Trends Cogn Sci, 15*(9), 401-408. doi: 10.1016/j.tics.2011.07.009

Bird, G., Silani, G., Brindley, R., White, S., Frith, U., & Singer, T. (2010). Empathic brain responses in insula are modulated by levels of alexithymia but not autism. *Brain, 133*(Pt 5), 1515-1525. doi: 10.1093/brain/awq060

Bond, C., LaForge, K. S., Tian, M., Melia, D., Zhang, S., Borg, L., . . . Yu, L. (1998). Single-nucleotide polymorphism in the human mu opioid receptor gene alters beta-endorphin binding and activity: possible implications for opiate addiction. *Proc Natl Acad Sci U S A, 95*(16), 9608-9613.

Borkenau, P., & Ostendorf, F. (1993). *NEO-Fünf-Faktoren Inventar (NEO-FFI) nach Costa und McCrae. Handanweisung.* . Göttingen: Hogrefe.

Bosch, J. A., de Geus, E. E., Ring, C., Nieuw Amerongen, A. V., & Stowell, J. R. (2004). Academic examinations and immunity: academic stress or examination stress? *Psychosom Med, 66*(4), 625-626; author reply 626-627. doi: 10.1097/01.psy.0000133254.46947.ac

Brown, S. M., Peet, E., Manuck, S. B., Williamson, D. E., Dahl, R. E., Ferrell, R. E., & Hariri, A. R. (2005). A regulatory variant of the human tryptophan hydroxylase-2 gene biases amygdala reactivity. *Mol Psychiatry, 10*(9), 884-888, 805. doi: 10.1038/sj.mp.4001716

Buckholtz, J. W., Callicott, J. H., Kolachana, B., Hariri, A. R., Goldberg, T. E., Genderson, M., . . . Meyer-Lindenberg, A. (2008). Genetic variation in MAOA modulates ventromedial prefrontal circuitry mediating individual differences in human personality. *Mol Psychiatry, 13*(3), 313-324. doi: 10.1038/sj.mp.4002020

Burcu, C., Arnrich, B., Marca, R., & Tröster, G. (2013). Monitoring of mental workload during an everyday life office-work scenario. *Journal Personal and Ubiquitous Computing, 17*(2), 229-239.

Burg, J. M., Wolf, O. T., & Michalak, J. (2012). Mindfulness as Self-Regulated Attention: Associations with Heart Rate Variability. *Swiss Journal of Psychology, 71*(3), 135-139.

Bussmann, J. B. J. (2013). One plus one equals three (or more…): Combining the assessment of movement behavior and subjective states in everyday life. *Front Psychol, 4*, 216.

Bussmann, J. B. J., & Ebner-Priemer, U. W. (2012). Ambulatory assessment of movement behavior: methodology, measurement and application. In M. Mehl & T. Conner (Eds.), *Handbook of Research Methods for Studying Daily Life*: Guilford Press.

Bylsma, L. M., Morris, B. H., & Rottenberg, J. (2008). A meta-analysis of emotional reactivity in major depressive disorder.*, 28*(4), 676-691. doi: 10.1016/j.cpr.2007.10.001

Canli, T., Congdon, E., Gutknecht, L., Constable, R. T., & Lesch, K. P. (2005a). Amygdala responsiveness is modulated by tryptophan hydroxylase-2 gene variation. *J Neural Transm, 112*(11), 1479-1485. doi: 10.1007/s00702-005-0391-4

Canli, T., Ferri, J., & Duman, E. A. (2009). Genetics of emotion regulation.*, 164*(1), 43-54. doi: 10.1016/j.neuroscience.2009.06.049

Canli, T., Omura, K., Haas, B. W., Fallgatter, A., Constable, R. T., & Lesch, K. P. (2005b). Beyond affect: a role for genetic variation of the serotonin transporter in neural activation during a cognitive attention task. *Proc Natl Acad Sci U S A, 102*(34), 12224-12229. doi: 10.1073/pnas.0503880102

Carriere, J. S., Seli, P., & Smilek, D. (2013). Wandering in both mind and body: individual differences in mind wandering and inattention predict fidgeting. *Can J Exp Psycho, 67*(1), 19-31.

Carroll, D., Ring, C., Shrimpton, J., Evans, P., Willemsen, G., & Hucklebridge, F. (1996). Secretory immunoglobulin A and cardiovascular responses to acute psychological challenge. *Int J Behav Med, 3*(3), 266-279. doi: 10.1207/s15327558ijbm0303_6

Carson, J. W., Carson, K. M., Gil, K. M., & Baucom, D. H. (2004). Mindfulness-Based Relationship Enhancement. *Behavior Therapy, 35*, 471-494.

Carson, J. W., Keefe, F. J., Lynch, T. R., Carson, K. M., Goli, V., Fras, A. M., & Thorp, S. R. (2005). Loving-Kindness Meditation for Chronic Low Back Pain. *Journal of Holistic Nursing, 23*(3), 287-304. doi: 10.1177/0898010105277651

Caspi, A., Sugden, K., Moffitt, T. E., Taylor, A., Craig, I. W., Harrington, H., . . . Poulton, R. (2003). Influence of life stress on depression: moderation by a polymorphism in the 5-HTT gene. *Science, 301*(5631), 386-389. doi: 10.1126/science.1083968

Catalino, L. I., & Fredrickson, B. L. (2011). A Tuesday in the life of a flourisher: the role of positive emotional reactivity in optimal mental health.*, 11*(4), 938-950. doi: 10.1037/a0024889

Cawthon, R. M., Smith, K. R., O'Brien, E., Sivatchenko, A., & Kerber, R. A. (2003). Association between telomere length in blood and mortality in people aged 60 years or older. *Lancet, 361*(9355), 393-395. doi: 10.1016/S0140-6736(03)12384-7

Chakrabarti, B., Kent, L., Suckling, J., Bullmore, E., & Baron-Cohen, S. (2006). Variations in the human cannabinoid receptor (CNR1) gene modulate striatal responses to happy faces. *Eur J Neurosci, {Hellhammer, 2007 #66}23*(7), 1944-1948. doi: 10.1111/j.1460-9568.2006.04697.x

Chan, S. R., & Blackburn, E. H. (2004). Telomeres and telomerase. *Philos Trans R Soc Lond B Biol Sci, 359*(1441), 109-121. doi: 10.1098/rstb.2003.1370

Chiesa, A., & Serretti, A. (2009). Mindfulness-based stress reduction for stress management in healthy people: a review and meta-analysis. *Journal of alternative and complementary medicine (New York, N.Y.), 15*(5), 593-600.

Chipchase, L. S., Schabrun, S. M., & Hodges, P. W. (2011a). Corticospinal excitability is dependent on the parameters of peripheral electric stimulation: a preliminary study. *Arch Phys Med Rehabil, 92*(9), 1423-1430. doi: 10.1016/j.apmr.2011.01.011

Chipchase, L. S., Schabrun, S. M., & Hodges, P. W. (2011b). Peripheral electrical stimulation to induce cortical plasticity: a systematic review of stimulus parameters. *Clin Neurophysiol, 122*(3), 456-463. doi: 10.1016/j.clinph.2010.07.025

Conner, T. S., Tennen, H., Fleeson, W., & Barrett, L. F. (2009a). Experience Sampling Methods: A Modern Idiographic Approach to Personality Research. *Soc Personal Psychol Compass, 3*(3), 292-313.

Conner, T. S., Tennen, H., Fleeson, W., & Barrett, L. F. (2009b). Experience Sampling Methods: A Modern Idiographic Approach to Personality Research.*, 3*(3), 292-313. doi: 10.1111/j.1751-9004.2009.00170.x

Copeland, W. E., Sun, H., Costello, E. J., Angold, A., Heilig, M. A., & Barr, C. S. (2011). Child mu-opioid receptor gene variant influences parent-child relations. *Neuropsychopharmacology, 36*(6), 1165-1170. doi: 10.1038/npp.2010.251

Corbetta, M., Patel, G., & Shulman, G. L. (2008). The reorienting system of the human brain: from environment to theory of mind. *Neuron, 58*(3), 306-324. doi: 10.1016/j.neuron.2008.04.017

Critchley, H. D., Wiens, S., Rotshtein, P., Ohman, A., & Dolan, R. J. (2004). Neural systems supporting interoceptive awareness. *Nat Neurosci, 7*(2), 189-195. doi: 10.1038/nn1176

Dahme, B., Richter, R., & Mass, R. (1996). Interoception of respiratory resistance in asthmatic patients. *Biol Psychol, 42*(1-2), 215-229. doi: 10.1016/0301-0511(95)05156-2

Davenport, P. W., Chan, P.-Y. S., Zhang, W., & Chou, Y.-L. (2007). Detection threshold for inspiratory resistive loads and respiratory-related evoked potentials. *Journal of Applied Physiology, 102*(1), 276-285. doi: 10.1152/japplphysiol.01436.2005

Davis, M. H. (1980). A multidimensional approach to individual differences in empathy. . *JSAS Catalog of Selected Documents in Psychology, 10*, 85.

de Jong, P. J., Koster, E. H., van Wees, R., & Martens, S. (2010). Angry facial expressions hamper subsequent target identification. *Emotion, 10*(5), 727-732. doi: 10.1037/a0019353

Depue, R. A., & Morrone-Strupinsky, J. V. (2005). A neurobehavioral model of affiliative bonding: implications for conceptualizing a human trait of affiliation. *Behav Brain Sci, 28*(3), 313-350; discussion 350-395. doi: 10.1017/S0140525X05000063

Diatchenko, L., Nackley, A. G., Slade, G. D., Bhalang, K., Belfer, I., Max, M. B., . . . Maixner, W. (2006). Catechol-O-methyltransferase gene polymorphisms are associated with multiple pain-evoking stimuli. *Pain, 125*(3), 216-224. doi: 10.1016/j.pain.2006.05.024

Dickerson, S. S., & Kemeny, M. E. (2004). Acute stressors and cortisol responses: a theoretical integration and synthesis of laboratory research. *Psychol Bull, 130*(3), 355-391. doi: 10.1037/0033-2909.130.3.355

Dixon, T. M., & Baumeister, R. F. (1991). Escaping the Self - the Moderating Effect of Self-Complexity. *Personality and Social Psychology Bulletin, 17*(4), 363-368. doi: 10.1177/0146167291174002

Dobkin, P. L., Irving, J. A., & Amar, S. (2011). For Whom May Participation in a Mindfulness-Based Stress Reduction Program be Contraindicated? *Mindfulness, 3*(1), 44-50. doi: 10.1007/s12671-011-0079-9

Doucette-Stamm, L. A., Blakely, D. J., Tian, J., Mockus, S., & Mao, J. I. (1995). Population genetic study of the human dopamine transporter gene (DAT1). *Genet Epidemiol, 12*(3), 303-308. doi: 10.1002/gepi.1370120307

Drabant, E. M., Hariri, A. R., Meyer-Lindenberg, A., Munoz, K. E., Mattay, V. S., Kolachana, B. S., . . . Weinberger, D. R. (2006). Catechol O-methyltransferase val158met genotype and neural mechanisms related to affective arousal and regulation. *Arch Gen Psychiatry, 63*(12), 1396-1406. doi: 10.1001/archpsyc.63.12.1396

Dreisbach, G., & Goschke, T. (2004). How positive affect modulates cognitive control: reduced perseveration at the cost of increased distractibility.*, 30*(2), 343-353. doi: 10.1037/0278-7393.30.2.343

Dux, P. E., & Marois, R. (2009). The attentional blink: a review of data and theory.*, 71*(8), 1683-1700. doi: 10.3758/APP.71.8.1683

Dziobek, I., Fleck, S., Kalbe, E., Rogers, K., Hassenstab, J., Brand, M., . . . Convit, A. (2006). Introducing MASC: a movie for the assessment of social cognition. *J Autism Dev Disord, 36*(5), 623-636. doi: 10.1007/s10803-006-0107-0

Ekman, P., & Friesen, W. V. (1978). *Facial Action Coding System: A Technique for the Measurement of Facial Movement.* . Palo Alto: Consulting Psychologists Press.

Ekman, P., Friesen, W. V., & Hager, J. C. (2002). Facial Action Coding System: The Manual on CD ROM. A Human Face. Salt Lake City.

Emmons, R. A., & McCullough, M. E. (2003). Counting blessings versus burdens: an experimental investigation of gratitude and subjective well-being in daily life. *J Pers Soc Psychol, 84*(2), 377-389. doi: 10.1037/0022-3514.84.2.377

Epel, E. S. (2009). Psychological and metabolic stress: a recipe for accelerated cellular aging? *Hormones (Athens), 8*(1), 7-22.

Eriksen, B. A., & Eriksen, C. W. (1974). Effects of noise letters upon the identification of a target letter in a nonsearch task *Perception & Psychophysics, 16*, 143-149.

Erk, S., Meyer-Lindenberg, A., Schnell, K., Opitz von Boberfeld, C., Esslinger, C., Kirsch, P., . . . Walter, H. (2010). Brain function in carriers of a genome-wide supported bipolar disorder variant. *Arch Gen Psychiatry, 67*(8), 803-811. doi: 10.1001/archgenpsychiatry.2010.94

Ernst, J., Northoff, G., Böker, H., Seifritz, E., & Grimm, S. (2012). Interoceptive awareness enhances neural activity during empathy. *Hum Brain Mapp*, n/a-n/a. doi: 10.1002/hbm.22014

Evans, D. W. (1994). Self-complexity and its relation to development, symptomatology and self-perception during adolescence. *Child Psychiatry Hum Dev, 24*(3), 173-182.

Fan, J., McCandliss, B. D., Fossella, J., Flombaum, J. I., & Posner, M. I. (2005). The activation of attentional networks. *Neuroimage, 26*(2), 471-479. doi: 10.1016/j.neuroimage.2005.02.004

Fan, J., McCandliss, B. D., Sommer, T., Raz, A., & Posner, M. I. (2002). Testing the efficiency and independence of attentional networks. *J Cogn Neurosci, 14*(3), 340-347. doi: 10.1162/089892902317361886

Fehr, E., & Fischbacher, U. (2004). Social norms and human cooperation. *Trends Cogn Sci, 8*(4), 185-190. doi: 10.1016/j.tics.2004.02.007

Fehr, E., & Schmidt, K. (1999). A theory of fairness, competition, and cooperation. *Quarterly Journal of Economics, 114*, 817-868.

Ferreira, M. A., O'Donovan, M. C., Meng, Y. A., Jones, I. R., Ruderfer, D. M., Jones, L., . . . Wellcome Trust Case Control, C. (2008). Collaborative genome-wide association analysis supports a role for ANK3 and CACNA1C in bipolar disorder. *Nat Genet, 40*(9), 1056-1058. doi: 10.1038/ng.209

Fillingim, R. B., Kaplan, L., Staud, R., Ness, T. J., Glover, T. L., Campbell, C. M., . . . Wallace, M. R. (2005). The A118G single nucleotide polymorphism of the mu-opioid receptor gene (OPRM1) is associated with pressure pain sensitivity in humans. *J Pain, 6*(3), 159-167. doi: 10.1016/j.jpain.2004.11.008

Fossel, M. (2002). Cell senescence in human aging and disease. *Ann N Y Acad Sci, 959*, 14-23.

Frattaroli, J. (2006). Experimental disclosure and its moderators: a meta-analysis. *Psychol Bull, 132*(6), 823-865. doi: 10.1037/0033-2909.132.6.823

Fredrickson, B. L., Cohn, M. A., Coffey, K. A., Pek, J., & Finkel, S. M. (2008). Open hearts build lives: positive emotions, induced through loving-kindness meditation, build consequential personal resources. *J Pers Soc Psychol, 95*(5), 1045-1062. doi: 10.1037/a0013262

Frenck, R. W., Jr., Blackburn, E. H., & Shannon, K. M. (1998). The rate of telomere sequence loss in human leukocytes varies with age. *Proc Natl Acad Sci U S A, 95*(10), 5607-5610.

Froese, T., & Fuchs, T. (2012). The extended body: A case study in the neurophenomenology of social interaction. *Phenomenology and the Cognitive Sciences, 11*, 205-235.

Furman, D. J., Chen, M. C., & Gotlib, I. H. (2011). Variant in oxytocin receptor gene is associated with amygdala volume. *Psychoneuroendocrinology, 36*(6), 891-897. doi: 10.1016/j.psyneuen.2010.12.004

Gable, S. L., Reis, H. T., Impett, E. A., & Asher, E. R. (2004). What do you do when things go right? The intrapersonal and interpersonal benefits of sharing positive events. *Journal of Personality and Social Psychology, 87*, 228–245.

Galili-Weisstub, E., & Segman, R. H. (2003). Attention deficit and hyperactivity disorder: review of genetic association studies. *Isr J Psychiatry Relat Sci, 40*(1), 57-66.

Gallagher, H. L., & Frith, C. D. (2003). Functional imaging of 'theory of mind'. *Trends Cogn Sci, 7*(2), 77-83. doi: 10.1016/s1364-6613(02)00025-6

Gentry, J. E., Baggerly, J., & Baranowsky, A. (2004). Training-as-treatment: effectiveness of the Certified Compassion Fatigue Specialist Training. *Int J Emerg Ment Health, 6*(3), 147-155.

Germer, C. K. (2009). *The Mindful Path to Self-Compassion*. New York: Guilford Press.

Gilbert, P. (2009a). *Compassion focused therapy*. London: Taylor & Francis.

Gilbert, P. (2009b). Introducing compassion-focused therapy. *Advances in Psychiatric Treatment, 15*(3), 199-208. doi: 10.1192/apt.bp.107.005264

Gilbert, P. (2010). *The Compassionate Mind*. London: Constable & Robinson Ltd.

Gilbert, P., & Procter, S. (2006). Compassionate mind training for people with high shame and self-criticism: overview and pilot study of a group therapy approach. *Clinical Psychology & Psychotherapy, 13*(6), 353-379. doi: 10.1002/cpp.507

Gizer, I. R., Ficks, C., & Waldman, I. D. (2009). Candidate gene studies of ADHD: a meta-analytic review. *Hum Genet, 126*(1), 51-90. doi: 10.1007/s00439-009-0694-x

Goetz, J., L., Keltner, D., & Simon-Thomas, E. (2010). Compassion: An Evolutionary Analysis and Empirical Review. *Psychol Bull, 136*(3), 351–374.

Goldberg, T. E., Egan, M. F., Gscheidle, T., Coppola, R., Weickert, T., Kolachana, B. S., . . . Weinberger, D. R. (2003). Executive subprocesses in working memory: relationship to catechol-O-methyltransferase Val158Met genotype and schizophrenia. *Arch Gen Psychiatry, 60*(9), 889-896. doi: 10.1001/archpsyc.60.9.889

Goldin, P. R., Manber, T., Hakimi, S., Canli, T., & Gross, J. J. (2009). Neural bases of social anxiety disorder: emotional reactivity and cognitive regulation during social and physical threat*., 66*(2), 170-180. doi: 10.1001/archgenpsychiatry.2008.525

Goldin, P. R., McRae, K., Ramel, W., & Gross, J. J. (2008). The neural bases of emotion regulation: reappraisal and suppression of negative emotion*., 63*(6), 577-586. doi: 10.1016/j.biopsych.2007.05.031

Grant, J. A., Courtemanche, J., & Rainville, P. (2011). A non-elaborative mental stance and decoupling of executive and pain-related cortices predicts low pain sensitivity in Zen meditators. *Pain, 152*(1), 150-156. doi: 10.1016/j.pain.2010.10.006

Greenwood, P. M., Sundararajan, R., Lin, M. K., Kumar, R., Fryxell, K. J., & Parasuraman, R. (2009). Both a nicotinic single nucleotide polymorphism (SNP) and a noradrenergic SNP modulate working memory performance when attention is manipulated. *J Cogn Neurosci, 21*(11), 2139-2153. doi: 10.1162/jocn.2008.21164

Gross, J. J. (2007). Emotion regulation: Conceptual foundations.

Gross, J. J., & Barrett, L. F. (2011). Emotion Generation and Emotion Regulation: One or Two Depends on Your Point of View. *Emot Rev, 3*(1), 8-16. doi: 10.1177/1754073910380974

Gross, J. J., Sheppes, G., & Urry, H. L. (2011). Cognition and Emotion Lecture at the 2010 SPSP Emotion Preconference Emotion generation and emotion regulation: A distinction we should make (carefully). *Cognition & Emotion, 25*(5), 765-781. doi: Doi 10.1080/02699931.2011.555753

Grossman, P., Niemann, L., Schmidt, S., & Walach, H. (2004). Mindfulness-based stress reduction and health benefits. A meta-analysis. *J Psychosom Res, 57*(1), 35-43. doi: 10.1016/S0022-3999(03)00573-7

Gutknecht, L., Jacob, C., Strobel, A., Kriegebaum, C., Muller, J., Zeng, Y., . . . Lesch, K. P. (2007). Tryptophan hydroxylase-2 gene variation influences personality traits and disorders related to emotional dysregulation. *Int J Neuropsychopharmacol, 10*(3), 309-320. doi: 10.1017/S1461145706007437

Hariri, A. R., Drabant, E. M., Munoz, K. E., Kolachana, B. S., Mattay, V. S., Egan, M. F., & Weinberger, D. R. (2005). A susceptibility gene for affective disorders and the response of the human amygdala. *Arch Gen Psychiatry, 62*(2), 146-152. doi: 10.1001/archpsyc.62.2.146

Hariri, A. R., Drabant, E. M., & Weinberger, D. R. (2006). Imaging genetics: perspectives from studies of genetically driven variation in serotonin function and corticolimbic affective processing. *Biol Psychiatry, 59*(10), 888-897. doi: 10.1016/j.biopsych.2005.11.005

Hariri, A. R., Mattay, V. S., Tessitore, A., Kolachana, B., Fera, F., Goldman, D., . . . Weinberger, D. R. (2002). Serotonin transporter genetic variation and the response of the human amygdala. *Science, 297*(5580), 400-403. doi: 10.1126/science.1071829

Hayes, S., & Pierson, H. (2005). Acceptance and Commitment Therapy Encyclopedia of Cognitive Behavior Therapy. In A. Freeman, S. H. Felgoise, C. M. Nezu, A. M. Nezu & M. A. Reinecke (Eds.), (pp. 1-4): Springer US.

Hein, G., Lamm, C., Brodbeck, C., & Singer, T. (2011). Skin conductance response to the pain of others predicts later costly helping. *PLoS One, 6*(8), e22759. doi: 10.1371/journal.pone.0022759

Hein, G., Silani, G., Preuschoff, K., Batson, C. D., & Singer, T. (2010). Neural responses to ingroup and outgroup members' suffering predict individual differences in costly helping. *Neuron, 68*(1), 149-160. doi: 10.1016/j.neuron.2010.09.003

Heinz, A., Braus, D. F., Smolka, M. N., Wrase, J., Puls, I., Hermann, D., . . . Buchel, C. (2005). Amygdala-prefrontal coupling depends on a genetic variation of the serotonin transporter. *Nat Neurosci, 8*(1), 20-21. doi: 10.1038/nn1366

Hellhammer, J., Fries, E., Schweisthal, O. W., Schlotz, W., Stone, A. A., & Hagemann, D. (2007). Several daily measurements are necessary to reliably assess the cortisol rise after awakening: state- and trait components. *Psychoneuroendocrinology, 32*(1), 80-86.

Henrich, J., McElreath, R., Barr, A., Ensminger, J., Barrett, C., Bolyanatz, A., . . . Ziker, J. (2006). Costly punishment across human societies. *Science, 312*(5781), 1767-1770. doi: 10.1126/science.1127333

Herbert, B. M., Herbert, C., & Pollatos, O. (2011). On the relationship between interoceptive awareness and alexithymia: is interoceptive awareness related to emotional awareness? *J Pers, 79*(5), 1149-1175. doi: 10.1111/j.1467-6494.2011.00717.x

Hofmann, S. G., Grossman, P., & Hinton, D. E. (2011). Loving-kindness and compassion meditation: potential for psychological interventions. *Clin Psychol Rev, 31*(7), 1126-1132. doi: 10.1016/j.cpr.2011.07.003

Holmes, T. (2007). *Parts Work: An Illustrated Guide to Your Inner Life*. Kalamazoo: Winged Heart Press.

Holmes, T. (2010). *Reisen in die Innenwelt*. München: Kösel-Verlag.

Hout, M. C., Goldinger, S. D., & Ferguson, R. W. (2012). The Versatility of SpAM: A Fast, Efficient, Spatial Method of Data Collection for Multidimensional Scaling. doi: 10.1037/a0028860

Hu, X. Z., Lipsky, R. H., Zhu, G., Akhtar, L. A., Taubman, J., Greenberg, B. D., . . . Goldman, D. (2006). Serotonin transporter promoter gain-of-function genotypes are linked to obsessive-compulsive disorder. *Am J Hum Genet, 78*(5), 815-826. doi: 10.1086/503850

Hutcherson, C. A., Seppala, E. M., & Gross, J. J. (2008). Loving-kindness meditation increases social connectedness. *Emotion, 8*(5), 720-724. doi: 10.1037/a0013237

Inoue, H., Yamasue, H., Tochigi, M., Abe, O., Liu, X., Kawamura, Y., . . . Kasai, K. (2010). Association between the oxytocin receptor gene and amygdalar volume in healthy adults. *Biol Psychiatry, 68*(11), 1066-1072. doi: 10.1016/j.biopsych.2010.07.019

Israel, S., Lerer, E., Shalev, I., Uzefovsky, F., Riebold, M., Laiba, E., . . . Ebstein, R. P. (2009). The oxytocin receptor (OXTR) contributes to prosocial fund allocations in the dictator game and the social value orientations task. *PLoS One, 4*(5), e5535. doi: 10.1371/journal.pone.0005535

Jabbi, M., Korf, J., Kema, I. P., Hartman, C., van der Pompe, G., Minderaa, R. B., . . . den Boer, J. A. (2007). Convergent genetic modulation of the endocrine stress response involves polymorphic variations of 5-HTT, COMT and MAOA. *Mol Psychiatry, 12*(5), 483-490. doi: 10.1038/sj.mp.4001975

Jezova, D., Skultetyova, I., Tokarev, D. I., Bakos, P., & Vigas, M. (1995). Vasopressin and oxytocin in stress. *Ann N Y Acad Sci, 771*, 192-203.

Jha, A. P., Stanley, E. A., Kiyonaga, A., Wong, L., & Gelfand, L. (2010). Examining the protective effects of mindfulness training on working memory capacity and affective experience. *Emotion, 10*(1), 54-64. doi: 10.1037/a0018438

Jinpa, T., Rosenberg, E., McGonigal, K., Cullen, M., Goldin, P., & Ramel, W. (2009). *Compassion Cultivation Training*. Protokoll bezogen über persönliche Kommunikation mit den Autoren.

Johnson, D. R. (2009). Emotional attention set-shifting and its relationship to anxiety and emotion regulation. *Emotion, 9*(5), 681-690. doi: 10.1037/a0017095

Kabat-Zinn, J. (2006). *Gesund durch Meditation: Das große Buch der Selbstheilung*. Frankfurt: Fischer.

Kajantie, E., & Phillips, D. I. (2006). The effects of sex and hormonal status on the physiological response to acute psychosocial stress. *Psychoneuroendocrinology, 31*(2), 151-178. doi: 10.1016/j.psyneuen.2005.07.002

Kanai, R., & Rees, G. (2011). Opinion: The structural basis of inter-individual differences in human behaviour and cognition. *Nature Reviews Neuroscience, 12*, 231-242

Kane, M. J., Brown, L. H., McVay, J. C., Silvia, P. J., Myin-Germeys, I., & Kwapil, T. R. (2007). For whom the mind wanders, and when: an experience-sampling study of working memory and executive control in daily life*., 18*(7), 614-621. doi: 10.1111/j.1467-9280.2007.01948.x

Kemeny, M., Ekman, W., Wallace, A., Giese-Davis, J., & Tsai, J. (2002). *Protokoll bezogen über persönliche Komminkation mit den Autoren*.

Kiesel, A., Steinhauser, M., Wendt, M., Falkenstein, M., Jost, K., Philipp, A. M., & Koch, I. (2010). Control and interference in task switching--a review. *Psychol Bull, 136*(5), 849-874. doi: 10.1037/a0019842

Killgore, W. D. (1998). The Affect Grid: a moderately valid, nonspecific measure of pleasure and arousal. *Psychol Rep, 83*(2), 639-642.

Killingsworth, M. A., & Gilbert, D. T. (2010). A Wandering Mind Is an Unhappy Mind. *Science, 330*(6006), 932. doi: 10.1126/science.1192439

Kim, H., Neubert, J. K., San Miguel, A., Xu, K., Krishnaraju, R. K., Iadarola, M. J., . . . Dionne, R. A. (2004). Genetic influence on variability in human acute experimental pain sensitivity associated with gender, ethnicity and psychological temperament. *Pain, 109*(3), 488-496. doi: 10.1016/j.pain.2004.02.027

King, L. A. (2002). Gain without pain? Expressive writing and self-regulation. In L. S. J. & J. M. Smyth (Eds.), *The writing cure: How expressive writing promotes health and emotional well-being*. Washington, DC: American Psychological Association.

Kirschbaum, C., & Hellhammer, D. (1989). Response variability of salivary cortisol under psychological stimulation. *J Clin Chem Clin Biochem, 27*(4), 237.

Kirschbaum, C., Pirke, K. M., & Hellhammer, D. H. (1993). The 'Trier Social Stress Test'--a tool for investigating psychobiological stress responses in a laboratory setting. *Neuropsychobiology, 28*(1-2), 76-81. doi: 119004

Klein, K., & Boals, A. (2001). Expressive writing can increase working memory capacity. *Journal of Experimental Psychology: General, 130*(3), 520-533. doi: 10.1037/0096-3445.130.3.520

Klimecki, O., & Singer, T. (2012). Empathic distress fatigue rather than compassion fatigue? Integrating findings from empathy research in psychology and social neuroscience. In B. Oakley, A. Knafo, G. Madhavan & D. S. Wilson (Eds.), *Pathological altruism* (pp. 368-383). New York: Oxford University Press.

Klimecki, O. M., Leiberg, S., Lamm, C., & Singer, T. (2012). Functional Neural Plasticity and Associated Changes in Positive Affect After Compassion Training. *Cereb Cortex*. doi: 10.1093/cercor/bhs142

Knafo, A., Israel, S., Darvasi, A., Bachner-Melman, R., Uzefovsky, F., Cohen, L., . . . Ebstein, R. P. (2008). Individual differences in allocation of funds in the dictator game associated with length of the arginine vasopressin 1a receptor RS3 promoter region and correlation between RS3 length and hippocampal mRNA. *Genes Brain Behav, 7*(3), 266-275. doi: 10.1111/j.1601-183X.2007.00341.x

Kolassa, I. T., Kolassa, S., Ertl, V., Papassotiropoulos, A., & De Quervain, D. J. (2010). The risk of posttraumatic stress disorder after trauma depends on traumatic load and the catechol-o-methyltransferase Val(158)Met polymorphism. *Biol Psychiatry, 67*(4), 304-308. doi: 10.1016/j.biopsych.2009.10.009

Koppenhöfer, E. (2004). *Kleine Schule des Genießens*. Lengerich: Pabst.

Kotsou, I., Nelis, D., Grégoire, J., & Mikolajczak, M. (2011). Emotional plasticity: Conditions and effects of improving emotional competence in adulthood. *Journal of Applied Psychology, 96*(4), 827-839. doi: 10.1037/a0023047

Koyama, T., McHaffie, J. G., Laurienti, P. J., & Coghill, R. C. (2005). The subjective experience of pain: where expectations become reality. *Proc Natl Acad Sci U S A, 102*(36), 12950-12955. doi: 10.1073/pnas.0408576102

Kupfer, J., Brosig, B., & Braehler, E. (2001). *TAS-26: Toronto-Alexithymie-Skala-26 (deutsche Version), Manual*. Göttingen: Hogrefe.

Kuppens, P., Allen, N. B., & Sheeber, L. B. (2010). Emotional inertia and psychological maladjustment.*, 21*(7), 984-991. doi: 10.1177/0956797610372634

Kuppens, P., Sheeber, L. B., Yap, M. B. H., Whittle, S., Simmons, J. G., & Allen, N. B. (2012). Emotional inertia prospectively predicts the onset of depressive disorder in adolescence.*, 12*(2), 283-289. doi: 10.1037/a0025046

Kustanovich, V., Ishii, J., Crawford, L., Yang, M., McGough, J. J., McCracken, J. T., . . . Nelson, S. F. (2004). Transmission disequilibrium testing of dopamine-related candidate gene polymorphisms in ADHD: confirmation of association of ADHD with DRD4 and DRD5. *Mol Psychiatry, 9*(7), 711-717. doi: 10.1038/sj.mp.4001466

Lachman, H. M., Papolos, D. F., Saito, T., Yu, Y. M., Szumlanski, C. L., & Weinshilboum, R. M. (1996). Human catechol-O-methyltransferase pharmacogenetics: description of a functional polymorphism and its potential application to neuropsychiatric disorders. *Pharmacogenetics, 6*(3), 243-250.

Lamm, C., Decety, J., & Singer, T. (2011). Meta-analytic evidence for common and distinct neural networks associated with directly experienced pain and empathy for pain. *Neuroimage, 54*(3), 2492-2502. doi: 10.1016/j.neuroimage.2010.10.014

Lang, P. J., Bradley, M. M., & Cuthbert, B. N. (2008). *International affective picture system (IAPS): Affective ratings of pictures and instruction manual. Technical Report A-8.* University of Florida, Gainesville, FL.

Le Merrer, J., Becker, J. A. J., Befort, K., & Kieffer, B. L. (2009). Reward processing by the opioid system in the brain. . *Physiol Rev, 89*, 1379-1412.

Ledesma, D., & Kumano, H. (2009). Mindfulness-based stress reduction and cancer: a meta-analysis. *Psycho-Oncology, 18*(6), 571-579. doi: 10.1002/pon.1400

Lee, M. R., Gallen, C. L., Zhang, X., Hodgkinson, C. A., Goldman, D., Stein, E. A., & Barr, C. S. (2011). Functional polymorphism of the mu-opioid receptor gene (OPRM1) influences reinforcement learning in humans. *PLoS One, 6*(9), e24203. doi: 10.1371/journal.pone.0024203

Lehrer, P. M., Vaschillo, E., & Vaschillo, B. (2000). Resonant Frequency Biofeedback Training to Increase Cardiac Variability: Rationale and Manual for Training. *Applied Psychophysiology and Biofeedback, 25*(3), 177-191.

Leiberg, S., Klimecki, O., & Singer, T. (2011). Short-Term Compassion Training Increases Prosocial Behavior in a Newly Developed Prosocial Game. *PLoS One, 6*(3), e17798. doi: 10.1371/journal.pone.0017798

Lesch, K. P., Bengel, D., Heils, A., Sabol, S. Z., Greenberg, B. D., Petri, S., . . . Murphy, D. L. (1996). Association of anxiety-related traits with a polymorphism in the serotonin transporter gene regulatory region. *Science, 274*(5292), 1527-1531.

Levy, F. (1991). The dopamine theory of attention deficit hyperactivity disorder (ADHD). *Aust N Z J Psychiatry, 25*(2), 277-283.

Lindquist, K. A., Wager, T. D., Kober, H., Bliss-Moreau, E., & Barrett, L. F. (2012). The brain basis of emotion: A meta-analytic review. *35*(03), 121-202. doi: 10.1017/S0140525X11001476

Linville, P. W. (1985). Self-Complexity and Affective Extremity: Don't Put All of Your Eggs in One Cognitive Basket. *Social Cognition, 3*(1), 94-120. doi: 10.1521/soco.1985.3.1.94

Linville, P. W. (1987a). Self-complexity as a cognitive buffer against stress-related illness and depression. *J Pers Soc Psychol, 52*(4), 663-676. doi: 10.1037/0022-3514.52.4.663

Linville, P. W. (1987b). Self-complexity as a cognitive buffer against stress-related illness and depression. *J Pers Soc Psychol, 52*(4), 663-676. doi: 10.1037/0022-3514.52.4.663

Logan, G. D., Cowan, W. B., & Davis, K. A. (1984). On the ability to inhibit simple and choice reaction time responses: a model and a method. *J Exp Psychol Hum Percept Perform, 10*(2), 276-291.

Lonsdorf, T. B., Golkar, A., Lindstom, K. M., Fransson, P., Schalling, M., Ohman, A., & Ingvar, M. (2011). 5-HTTLPR and COMTval158met genotype gate amygdala reactivity and habituation. *Biol Psychol, 87*(1), 106-112. doi: 10.1016/j.biopsycho.2011.02.014

Lotrich, F. E., & Pollock, B. G. (2004). Meta-analysis of serotonin transporter polymorphisms and affective disorders. *Psychiatr Genet, 14*(3), 121-129.

Lotsch, J., Stuck, B., & Hummel, T. (2006). The human mu-opioid receptor gene polymorphism 118A > G decreases cortical activation in response to specific nociceptive stimulation. *Behav Neurosci, 120*(6), 1218-1224. doi: 10.1037/0735-7044.120.6.1218

Low, C. A., Stanton, A. L., & Bower, J. E. (2008). Effects of acceptance-oriented versus evaluative emotional processing on heart rate recovery and habituation. *Emotion, 8*(3), 419-424. doi: 10.1037/1528-3542.8.3.419

Lowe, N., Kirley, A., Mullins, C., Fitzgerald, M., Gill, M., & Hawi, Z. (2004). Multiple marker analysis at the promoter region of the DRD4 gene and ADHD: evidence of linkage and association with the SNP -616. *Am J Med Genet B Neuropsychiatr Genet, 131B*(1), 33-37. doi: 10.1002/ajmg.b.30071

Lucht, M. J., Barnow, S., Sonnenfeld, C., Rosenberger, A., Grabe, H. J., Schroeder, W., . . . Rosskopf, D. (2009). Associations between the oxytocin receptor gene (OXTR) and

affect, loneliness and intelligence in normal subjects. *Prog Neuropsychopharmacol Biol Psychiatry, 33*(5), 860-866. doi: 10.1016/j.pnpbp.2009.04.004

Luo, W., Watkins, D., & Lam, R. Y. (2009). Validating a new measure of self-complexity. *J Pers Assess, 91*(4), 381-386. doi: 10.1080/00223890902936223

Luoma, J., & Hayes, S. C. (2002). Cognitive defusion. In W. T. O'Donohue, J. E. Fisher & S. C. Hayes (Eds.), *Empirically supported techniques of cognitive behavior therapy: A step by step guide for clinicians*. New York: Wiley.

Lyubomirsky, S., Sheldon, K. M., & Schkade, D. (2005). Pursuing Happiness: The Architecture of Sustainable Change. *Review of General Psychology, 9*(2), 111-131. doi: 10.1037/1089-2680.9.2.111

Lyubomirsky, S., Sousa, L., & Dickerhoof, R. (2006). The costs and benefits of writing, talking, and thinking about life's triumphs and defeats. *J Pers Soc Psychol, 90*(4), 692-708. doi: 10.1037/0022-3514.90.4.692

MacLean, K. A., Ferrer, E., Aichele, S. R., Bridwell, D. A., Zanesco, A. P., Jacobs, T. L., . . . Saron, C. D. (2010). Intensive meditation training improves perceptual discrimination and sustained attention. *Psychol Sci, 21*(6), 829-839. doi: 10.1177/0956797610371339

Maguire, E. A., Gadian, D. G., Johnsrude, I. S., Good, C. D., Ashburner, J., Frackowiak, R. S., & Frith, C. D. (2000). Navigation-related structural change in the hippocampi of taxi drivers. *Proc Natl Acad Sci U S A, 97*(8), 4398-4403. doi: 10.1073/pnas.070039597

Maisel, N. C., Gable, S. L., & Strachman, A. (2008). Responsive behaviors in good times and in bad. *Personal Relationships, 15*(3), 317-338.

Malhotra, A. K., Kestler, L. J., Mazzanti, C., Bates, J. A., Goldberg, T., & Goldman, D. (2002). A functional polymorphism in the COMT gene and performance on a test of prefrontal cognition. *Am J Psychiatry, 159*(4), 652-654.

Mar, R. A. (2011). The neural bases of social cognition and story comprehension. *Annu Rev Psychol, 62*, 103-134. doi: 10.1146/annurev-psych-120709-145406

Maratos, F. A., Mogg, K., & Bradley, B. P. (2008). Identification of angry faces in the attentional blink. *Cogn Emot, 22*(7), 1340-1352. doi: 10.1080/02699930701774218

Markett, S., Montag, C., & Reuter, M. (2011). The nicotinic acetylcholine receptor gene CHRNA4 is associated with negative emotionality. *Emotion, 11*(2), 450-455. doi: 10.1037/a0021784

Markett, S. A., Montag, C., & Reuter, M. (2010). The association between dopamine DRD2 polymorphisms and working memory capacity is modulated by a functional polymorphism on the nicotinic receptor gene CHRNA4. *J Cogn Neurosci, 22*(9), 1944-1954. doi: 10.1162/jocn.2009.21354

Martens, S., & Johnson, A. (2009). Working memory capacity, intelligence, and the magnitude of the attentional blink revisited.*, 192*(1), 43-52. doi: 10.1007/s00221-008-1551-1

Mascaro, J. S., Rilling, J. K., Tenzin, N. L., & Raison, C. L. (in press). Compassion meditation enhances empathic accuracy and related neural activity. *Soc Cogn Affect Neurosci*.

McCall, C., & Blascovich, J. (2009). How, when, and why to use digital experimental virtual environments to study social behavior. *Social and Personality Psychology Compass*(3), 744-758.

McRae, K., Ciesielski, B., & Gross, J. J. (2012). Unpacking cognitive reappraisal: goals, tactics, and outcomes.*, 12*(2), 250-255. doi: 10.1037/a0026351

Merleau-Ponty, M. (1945). *Phénoménologie de la perception*. Paris: Gallimard.

Meyer-Lindenberg, A., Buckholtz, J. W., Kolachana, B., A, R. H., Pezawas, L., Blasi, G., . . . Weinberger, D. R. (2006). Neural mechanisms of genetic risk for impulsivity and violence in humans. *Proc Natl Acad Sci U S A, 103*(16), 6269-6274. doi: 10.1073/pnas.0511311103

Meyer-Lindenberg, A., Domes, G., Kirsch, P., & Heinrichs, M. (2011). Oxytocin and vasopressin in the human brain: social neuropeptides for translational medicine. *Nat Rev Neurosci, 12*(9), 524-538. doi: 10.1038/nrn3044

Michalak, J., Troje, N., & Heidenreich, T. (2011). The effects of mindfulness-based cognitive therapy on depressive gait patterns. *Journal of Cognitive and Behavioral Psychotherapies, 11*, 13-27.

Mickey, B. J., Zhou, Z., Heitzeg, M. M., Heinz, E., Hodgkinson, C. A., Hsu, D. T., . . . Zubieta, J. K. (2011). Emotion processing, major depression, and functional genetic variation of neuropeptide Y. *Arch Gen Psychiatry, 68*(2), 158-166. doi: 10.1001/archgenpsychiatry.2010.197

Miyake, A., & Friedman, N. P. (2012). The Nature and Organization of Individual Differences in Executive Functions: Four General Conclusions.*, 21*(1), 8-14. doi: 10.1177/0963721411429458

Mobes, J., Joppich, G., Stiebritz, F., Dengler, R., & Schroder, C. (2008). Emotional speech in Parkinson's disease. *Movement Disorders, 23*(6), 824-829. doi: 10.1002/mds.21940

Mones, A. G., & Schwartz, R. C. (2007). The functional hypothesis: A family systems contribution toward an understanding of the healing process of the common factors. *Journal of Psychotherapy Integration, 17*(4), 314-329. doi: 10.1037/1053-0479.17.4.314

Most, S. B., Chun, M. M., Widders, D. M., & Zald, D. H. (2005). Attentional rubbernecking: cognitive control and personality in emotion-induced blindness.*, 12*(4), 654-661.

Murphy, D. L., Lerner, A., Rudnick, G., & Lesch, K. P. (2004). Serotonin transporter: gene, genetic disorders, and pharmacogenetics. *Mol Interv, 4*(2), 109-123. doi: 10.1124/mi.4.2.8

Nater, U. M., & Rohleder, N. (2009). Salivary alpha-amylase as a non-invasive biomarker for the sympathetic nervous system: current state of research. *Psychoneuroendocrinology, 34*(4), 486-496. doi: 10.1016/j.psyneuen.2009.01.014

Neff, K. (2009). *Mindful Self-Compassion Training*. Protokoll bezogen über persönliche Kommunikation mit den Autoren.

Neff, K. (2011). *Self-Compassion*. New York: HarperCollins.

Negi, L. T. (2009). *Emory Compassion Meditation Protocol*. Protokoll bezogen über persönliche Kommunikation mit dem Autor.

Niedenthal, P. M. (2007). Embodying emotion. *Science, 316*(5827), 1002-1005. doi: 10.1126/science.1136930

Njajou, O. T., Hsueh, W. C., Blackburn, E. H., Newman, A. B., Wu, S. H., Li, R., . . . Health, A. B. C. s. (2009). Association between telomere length, specific causes of death, and years of healthy life in health, aging, and body composition, a population-based cohort study. *J Gerontol A Biol Sci Med Sci, 64*(8), 860-864. doi: 10.1093/gerona/glp061

Oldenhof, J., Vickery, R., Anafi, M., Oak, J., Ray, A., Schoots, O., . . . Van Tol, H. H. (1998). SH3 binding domains in the dopamine D4 receptor. *Biochemistry, 37*(45), 15726-15736. doi: 10.1021/bi981634+

Pace, T. W., Negi, L. T., Adame, D. D., Cole, S. P., Sivilli, T. I., Brown, T. D., . . . Raison, C. L. (2009). Effect of compassion meditation on neuroendocrine, innate immune and behavioral responses to psychosocial stress. *Psychoneuroendocrinology, 34*(1), 87-98. doi: 10.1016/j.psyneuen.2008.08.011

Panksepp, J. (2006). The core emotional systems of the mammalian brain: the fundamental substrates of human emotions. In J. Corrigall, H. Payne & H. Wilkinson (Eds.), *About a Body: working with the embodied mind in psychotherapy*. Routledge: Hove, UK & NYC.

Parasuraman, R. (1986). Vigilance, monitoring and search. In R. Boff, L. Kaufman & J. P. Thomas (Eds.), *Handbook of perception and human performance: Vol. 2. Cognitive processes and performance.* Oxford, England: Wiley.

Parasuraman, R., Greenwood, P. M., Kumar, R., & Fossella, J. (2005). Beyond heritability: neurotransmitter genes differentially modulate visuospatial attention and working memory. *Psychol Sci, 16*(3), 200-207. doi: 10.1111/j.0956-7976.2005.00804.x

Parker, A. G., Boldero, J. M., & Bell, R. C. (2006). Borderline personality disorder features: the role of self-discrepancies and self-complexity. *Psychol Psychother, 79*(Pt 3), 309-321. doi: 10.1348/147608305X70072

Pasupathi, M. (2003). Emotion regulation during social remembering: Differences between emotions elicited during an event and emotions elicited when talking about it. *Memory, 11*(2), 151-163. doi: 10.1080/741938212

Paulmann, S., Pell, M. D., & Kotz, S. A. (2008). How aging affects the recognition of emotional speech. *Brain and Language, 104*(3), 262-269. doi: 10.1016/j.bandl.2007.03.002

Perez-De-Albeniz, A., & Holmes, J. (2000). Meditation: Concepts, effects and uses in therapy. *International Journal of Psychotherapy, 5*(1), 49-58. doi: 10.1080/13569080050020263

Petitmengin, C., Baulac, M., & Navarro, V. (2006). Seizure anticipation: are neurophenomenological approaches able to detect preictal symptoms? *Epilepsy Behav, 9*(2), 298-306.

Pezawas, L., Meyer-Lindenberg, A., Drabant, E. M., Verchinski, B. A., Munoz, K. E., Kolachana, B. S., . . . Weinberger, D. R. (2005). 5-HTTLPR polymorphism impacts human cingulate-amygdala interactions: a genetic susceptibility mechanism for depression. *Nat Neurosci, 8*(6), 828-834. doi: 10.1038/nn1463

Plessow, F., Fischer, R., Kirschbaum, C., & Goschke, T. (2011). Inflexibly focused under stress: acute psychosocial stress increases shielding of action goals at the expense of reduced cognitive flexibility with increasing time lag to the stressor.*, 23*(11), 3218-3227. doi: 10.1162/jocn_a_00024

Pollatos, O., Gramann, K., & Schandry, R. (2007). Neural systems connecting interoceptive awareness and feelings. *Hum Brain Mapp, 28*(1), 9-18. doi: 10.1002/hbm.20258

Posner, M. I. (1980). Orienting of attention. *Q J Exp Psychol, 32*(1), 3-25.

Posner, M. I., & Rothbart, M. K. (2009). Toward a physical basis of attention and self regulation. *Phys Life Rev, 6*(2), 103-120. doi: 10.1016/j.plrev.2009.02.001

Pragst, F., & Balikova, M. A. (2006). State of the art in hair analysis for detection of drug and alcohol abuse. *Clin Chim Acta, 370*(1-2), 17-49.

Rafaeli, E., & Hiller, A. (2010). Self-Complexity - A Source of Resilience? In J. W. Reich, A. J. Zautra & J. S. Hall (Eds.), *Handbook of Adult Resilience* (pp. 171-192). New York: Guilford Press.

Rasch, B., Spalek, K., Buholzer, S., Luechinger, R., Boesiger, P., Papassotiropoulos, A., & de Quervain, D. J. (2009). A genetic variation of the noradrenergic system is related to differential amygdala activation during encoding of emotional memories. *Proc Natl Acad Sci U S A, 106*(45), 19191-19196. doi: 10.1073/pnas.0907425106

Ravizza, S. M., & Carter, C. S. (2008). Shifting set about task switching: behavioral and neural evidence for distinct forms of cognitive flexibility.*, 46*(12), 2924-2935. doi: 10.1016/j.neuropsychologia.2008.06.006

Raymond, J. E., & O'Brien, J. L. (2009). Selective visual attention and motivation: the consequences of value learning in an attentional blink task. *Psychol Sci, 20*(8), 981-988. doi: 10.1111/j.1467-9280.2009.02391.x

Reinvang, I., Lundervold, A. J., Rootwelt, H., Wehling, E., & Espeseth, T. (2009). Individual variation in a cholinergic receptor gene modulates attention. *Neurosci Lett, 453*(3), 131-134. doi: 10.1016/j.neulet.2009.02.029

Reuter, M., Esslinger, C., Montag, C., Lis, S., Gallhofer, B., & Kirsch, P. (2008). A functional variant of the tryptophan hydroxylase 2 gene impacts working memory: a genetic imaging study. *Biol Psychol, 79*(1), 111-117. doi: 10.1016/j.biopsycho.2007.12.002

Reuter, M., Ott, U., Vaitl, D., & Hennig, J. (2007). Impaired executive control is associated with a variation in the promoter region of the tryptophan hydroxylase 2 gene. *J Cogn Neurosci, 19*(3), 401-408. doi: 10.1162/jocn.2007.19.3.401

Rimé, B. (2007). Interpersonal Emotion Regulation. In J. J. Gross (Ed.), *Handbook of emotion regulation*. New York: Guilford Press.

Rodrigues, S. M., Saslow, L. R., Garcia, N., John, O. P., & Keltner, D. (2009). Oxytocin receptor genetic variation relates to empathy and stress reactivity in humans. *Proc Natl Acad Sci U S A, 106*(50), 21437-21441. doi: 10.1073/pnas.0909579106

Rogers, T. B., Kuiper, N. A., & Kirker, W. S. (1977). Self-reference and the encoding of personal information. *J Pers Soc Psychol, 35*(9), 677-688.

Rubin, L. H., Carter, C. S., Drogos, L., Pournajafi-Nazarloo, H., Sweeney, J. A., & Maki, P. M. (2010). Peripheral oxytocin is associated with reduced symptom severity in schizophrenia. *Schizophr Res, 124*(1-3), 13-21. doi: 10.1016/j.schres.2010.09.014

Rueda, M. R., Posner, M. I., & Rothbart, M. K. (2005). The development of executive attention: contributions to the emergence of self-regulation. *Dev Neuropsychol, 28*(2), 573-594. doi: 10.1207/s15326942dn2802_2

Sabol, S. Z., Hu, S., & Hamer, D. (1998). A functional polymorphism in the monoamine oxidase A gene promoter. *Hum Genet, 103*(3), 273-279.

Sahdra, B. K., MacLean, K. A., Ferrer, E., Shaver, P. R., Rosenberg, E. L., Jacobs, T. L., . . . Saron, C. D. (2011). Enhanced response inhibition during intensive meditation training predicts improvements in self-reported adaptive socioemotional functioning. *Emotion, 11*(2), 299-312. doi: 10.1037/a0022764

Salzberg, S. (1995). *Loving-Kindness - The Revolutionary Art of Happiness*. Boston, MA: Shambala Publications.

Samson, D., Apperly, I. A., Braithwaite, J. J., Andrews, B. J., & Bodley Scott, S. E. (2010). Seeing it their way: Evidence for rapid and involuntary computation of what other people see. *Journal of Experimental Psychology: Human Perception and Performance, 36*(5), 1255-1266. doi: 10.1037/a0018729

Saxe, R., & Kanwisher, N. (2003). People thinking about thinking people. The role of the temporo-parietal junction in "theory of mind". *Neuroimage, 19*(4), 1835-1842.

Schandry, R. (1981). Heart Beat Perception and Emotional Experience. *Psychophysiology, 18*(4), 483-488. doi: 10.1111/j.1469-8986.1981.tb02486.x

Schinka, J. A., Busch, R. M., & Robichaux-Keene, N. (2004). A meta-analysis of the association between the serotonin transporter gene polymorphism (5-HTTLPR) and trait anxiety. *Mol Psychiatry, 9*(2), 197-202. doi: 10.1038/sj.mp.4001405

Scholz, J., Klein, M. C., Behrens, T. E., & Johansen-Berg, H. (2009). Training induces changes in white-matter architecture. *Nat Neurosci, 12*(11), 1370-1371. doi: 10.1038/nn.2412

Schwartz, M. S., & Andrasik, F. (2003). *Biofeedback - A practitioner's guide*. New York: Guilford Press.

Schwartz, R. (2008). *IFS Das System der inneren Familie: Ein Weg zu mehr Selbstführung*. Norderstedt: Books on Demand.

Semple, R. (2010). Does Mindfulness Meditation Enhance Attention? A Randomized Controlled Trial. *Mindfulness, 1*(2), 121-130. doi: 10.1007/s12671-010-0017-2

Sen, S., Burmeister, M., & Ghosh, D. (2004). Meta-analysis of the association between a serotonin transporter promoter polymorphism (5-HTTLPR) and anxiety-related personality traits. *Am J Med Genet B Neuropsychiatr Genet, 127B*(1), 85-89. doi: 10.1002/ajmg.b.20158

Singer, T., Critchley, H. D., & Preuschoff, K. (2009). A common role of insula in feelings, empathy and uncertainty. *Trends Cogn Sci, 13*(8), 334-340. doi: 10.1016/j.tics.2009.05.001

Singer, T., Kiebel, S. J., Winston, J. S., Dolan, R. J., & Frith, C. D. (2004a). Brain responses to the acquired moral status of faces. *Neuron, 41*(4), 653-662.
Singer, T., & Lamm, C. (2009). The social neuroscience of empathy. *Ann N Y Acad Sci, 1156*, 81-96. doi: 10.1111/j.1749-6632.2009.04418.x
Singer, T., Seymour, B., O'Doherty, J., Kaube, H., Dolan, R. J., & Frith, C. D. (2004b). Empathy for pain involves the affective but not sensory components of pain. *Science, 303*(5661), 1157-1162. doi: 10.1126/science.1093535
Singer, T., Seymour, B., O'Doherty, J. P., Stephan, K. E., Dolan, R. J., & Frith, C. D. (2006). Empathic neural responses are modulated by the perceived fairness of others. *Nature, 439*(7075), 466-469. doi: 10.1038/nature04271
Singer, T., Snozzi, R., Bird, G., Petrovic, P., Silani, G., Heinrichs, M., & Dolan, R. J. (2008). Effects of oxytocin and prosocial behavior on brain responses to direct and vicariously experienced pain. *Emotion, 8*(6), 781-791. doi: 10.1037/a0014195
Singer, T., & Steinbeis, N. (2009). Differential roles of fairness- and compassion-based motivations for cooperation, defection, and punishment. *Ann N Y Acad Sci, 1167*, 41-50. doi: 10.1111/j.1749-6632.2009.04733.x
Slagter, H. A., Lutz, A., Greischar, L. L., Francis, A. D., Nieuwenhuis, S., Davis, J. M., & Davidson, R. J. (2007). Mental training affects distribution of limited brain resources., *5*(6), e138. doi: 10.1371/journal.pbio.0050138
Smallwood, J., Fitzgerald, A., Miles, L. K., & Phillips, L. H. (2009a). Shifting moods, wandering minds: negative moods lead the mind to wander. *Emotion, 9*(2), 271-276. doi: 10.1037/a0014855
Smallwood, J., Nind, L., & O'Connor, R. C. (2009b). When is your head at? An exploration of the factors associated with the temporal focus of the wandering mind. *Conscious Cogn, 18*(1), 118-125. doi: 10.1016/j.concog.2008.11.004
Smallwood, J., Schooler, J. W., Turk, D. J., Cunningham, S. J., Burns, P., & Macrae, C. N. (2011). Self-reflection and the temporal focus of the wandering mind. *Conscious Cogn, 20*(4), 1120-1126. doi: 10.1016/j.concog.2010.12.017
Smith, T. W., Cribbet, M. R., Nealey-Moore, J. B., Uchino, B. N., Williams, P. G., Mackenzie, J., & Thayer, J. F. (2011). Matters of the variable heart: respiratory sinus arrhythmia response to marital interaction and associations with marital quality. *J Pers Soc Psychol, 100*(1), 103-119. doi: 10.1037/a0021136
Smolka, M. N., Buhler, M., Schumann, G., Klein, S., Hu, X. Z., Moayer, M., . . . Heinz, A. (2007). Gene-gene effects on central processing of aversive stimuli. *Mol Psychiatry, 12*(3), 307-317. doi: 10.1038/sj.mp.4001946
Smolka, M. N., Schumann, G., Wrase, J., Grusser, S. M., Flor, H., Mann, K., . . . Heinz, A. (2005). Catechol-O-methyltransferase val158met genotype affects processing of emotional stimuli in the amygdala and prefrontal cortex. *J Neurosci, 25*(4), 836-842. doi: 10.1523/JNEUROSCI.1792-04.2005
Sodian, B., & Kristen, S. (2010). Theory of Mind:Towards a Theory of Thinking. In B. Glatzeder, V. Goel & A. Müller (Eds.), (pp. 189-201): Springer Berlin Heidelberg.
Somerville, L. H., Wagner, D. D., Wig, G. S., Moran, J. M., Whalen, P. J., & Kelley, W. M. (2012). Interactions Between Transient and Sustained Neural Signals Support the Generation and Regulation of Anxious Emotion. doi: 10.1093/cercor/bhr373
Somerville, L. H., Whalen, P. J., & Kelley, W. M. (2010). Human bed nucleus of the stria terminalis indexes hypervigilant threat monitoring., *68*(5), 416-424. doi: 10.1016/j.biopsych.2010.04.002
Southam-Gerow, M. A., & Kendall, P. C. (2002). Emotion regulation and understanding: implications for child psychopathology and therapy. *Clin Psychol Rev, 22*(2), 189-222. doi: 10.1016/s0272-7358(01)00087-3

Spielberger, C. D., Gorsuch, R. L., Lushene, R., Vagg, P. R., & Jacobs, G. A. (1983). *Manual for the State-Trait Anxiety Inventory*. Palo Alto, CA: Consulting Psychologists Press.

Stahl, B., Goldstein,E., Santorelli, S. Kabat-Zinn, J. (2010). *A Mindfulness-Based Stress Reduction Workbook*. Oakland, CA: New Harbinger Publications.

Stalder, T., Evans, P., Hucklebridge, F., & Clow, A. (2010). Associations between psychosocial state variables and the cortisol awakening response in a single case study. *Psychoneuroendocrinology, 35*(2), 209-214.

Stalder, T., & Kirschbaum, C. (2012). Analysis of cortisol in hair - State of the art and future direction. *Brain Behav Immun, 26*, 1019-1029.

Steptoe, A., O'Donnell, K., Badrick, E., Kumari, M., & Marmot, M. (2008). Neuroendocrine and inflammatory factors associated with positive affect in healthy men and women: the Whitehall II study. *Am J Epidemiol, 167*(1), 96-102. doi: 10.1093/aje/kwm252

Sternberg, S. (1966). High-speed scanning in human memory. *Science, 153*(3736), 652-654.

Sze, J. A., Gyurak, A., Yuan, J. W., & Levenson, R. W. (2010). Coherence between emotional experience and physiology: does body awareness training have an impact? *Emotion, 10*(6), 803-814. doi: 10.1037/a0020146

Tang, Y. Y., Ma, Y., Wang, J., Fan, Y., Feng, S., Lu, Q., . . . Posner, M. I. (2007). Short-term meditation training improves attention and self-regulation. *Proc Natl Acad Sci U S A, 104*(43), 17152-17156. doi: 10.1073/pnas.0707678104

Tang, Y. Y., & Posner, M. I. (2009). Attention training and attention state training. *Trends Cogn Sci, 13*(5), 222-227. doi: 10.1016/j.tics.2009.01.009

Terasawa, Y., Fukushima, H., & Umeda, S. (2011). How does interoceptive awareness interact with the subjective experience of emotion? An fMRI Study. *Hum Brain Mapp*, n/a-n/a. doi: 10.1002/hbm.21458

Thayer, J. F., Ahs, F., Fredrikson, M., Sollers, J. J., 3rd, & Wager, T. D. (2012). A meta-analysis of heart rate variability and neuroimaging studies: implications for heart rate variability as a marker of stress and health. *Neurosci Biobehav Rev, 36*(2), 747-756. doi: 10.1016/j.neubiorev.2011.11.009

Thayer, J. F., & Lane, R. D. (2009). Claude Bernard and the heart-brain connection: further elaboration of a model of neurovisceral integration. *Neurosci Biobehav Rev, 33*(2), 81-88. doi: 10.1016/j.neubiorev.2008.08.004

Thompson, R. A. (1994). Emotion regulation: a theme in search of definition. *Monogr Soc Res Child Dev, 59*(2-3), 25-52.

Toivonen, R., Kivela, M., Saramaki, J., Viinikainen, M., Vanhatalo, M., & Sams, M. (2012). Networks of emotion concepts. *PLoS One, 7*(1), e28883. doi: 10.1371/journal.pone.0028883

Tost, H., Kolachana, B., Hakimi, S., Lemaitre, H., Verchinski, B. A., Mattay, V. S., . . . Meyer-Lindenberg, A. (2010). A common allele in the oxytocin receptor gene (OXTR) impacts prosocial temperament and human hypothalamic-limbic structure and function. *Proc Natl Acad Sci U S A, 107*(31), 13936-13941. doi: 10.1073/pnas.1003296107

Trull, T. J., & Ebner-Priemer, U. (2013). Ambulatory Assessment. *Annu Rev Cli Psychol, 9*, 151-176.

Tugade, M. M., & Fredrickson, B. L. (2004). Resilient individuals use positive emotions to bounce back from negative emotional experiences*., 86*(2), 320-333. doi: 10.1037/0022-3514.86.2.320

Tugade, M. M., Fredrickson, B. L., & Barrett, L. F. (2004). Psychological resilience and positive emotional granularity: examining the benefits of positive emotions on coping and health*., 72*(6), 1161-1190. doi: 10.1111/j.1467-6494.2004.00294.x

Ukkola, L. T., Onkamo, P., Raijas, P., Karma, K., & Jarvela, I. (2009). Musical aptitude is associated with AVPR1A-haplotypes. *PLoS One, 4*(5), e5534. doi: 10.1371/journal.pone.0005534

van Leeuwen, S., Müller, N. G., & Melloni, L. (2009). Age effects on attentional blink performance in meditation.*, 18*(3), 593-599. doi: 10.1016/j.concog.2009.05.001

Van Overwalle, F. (2009). Social cognition and the brain: a meta-analysis. *Hum Brain Mapp, 30*(3), 829-858. doi: 10.1002/hbm.20547

Vandenbergh, D. J., Persico, A. M., Hawkins, A. L., Griffin, C. A., Li, X., Jabs, E. W., & Uhl, G. R. (1992). Human dopamine transporter gene (DAT1) maps to chromosome 5p15.3 and displays a VNTR. *Genomics, 14*(4), 1104-1106.

Varela, F., & Shear, J. (1999). The View from Within: First-Person Methodologies in the Study of Consciousness. *Journal of Consciousness Studies, 6*(2-3).

Varela, F. J., Thompson, E. T., & Rosch, E. (1992). *The Embodied Mind: Cognitive Science and Human Experience*. Cambridge, MA: The MIT Press.

Varela, F. T., E.; Rosch E. (1991). *The Embodied Mind: Cognitive Science and Human Experience*. Boston, MA: MIT Press.

Villien, F., Yu, M., Barthélémy, P., & Jammes, Y. (2005). Training to yoga respiration selectively increases respiratory sensation in healthy man. *Respiratory Physiology & Neurobiology, 146*(1), 85-96. doi: 10.1016/j.resp.2004.11.010

Vohs, K. D., & Baumeister, R. F. (Eds.). (2011). *Handbook of Self-Regulation*. New York: The Guilford Press.

Volkow, N. D., Ding, Y. S., Fowler, J. S., Wang, G. J., Logan, J., Gatley, J. S., . . . et al. (1995). Is methylphenidate like cocaine? Studies on their pharmacokinetics and distribution in the human brain. *Arch Gen Psychiatry, 52*(6), 456-463.

Wallace, B. A. (1996). *Choosing Reality*. Ithaca, NY: Snow Lion Publications.

Wallace, B. A. (2006). *The Attention Revolution*. Somerville, MA: Wisdom Publications.

Walum, H., Lichtenstein, P., Neiderhiser, J. M., Reiss, D., Ganiban, J. M., Spotts, E. L., . . . Westberg, L. (2012). Variation in the oxytocin receptor gene is associated with pair-bonding and social behavior. *Biol Psychiatry, 71*(5), 419-426. doi: 10.1016/j.biopsych.2011.09.002

Way, B. M., Taylor, S. E., & Eisenberger, N. I. (2009). Variation in the mu-opioid receptor gene (OPRM1) is associated with dispositional and neural sensitivity to social rejection. *Proc Natl Acad Sci U S A, 106*(35), 15079-15084. doi: 10.1073/pnas.0812612106

Zak, P. J., Kurzban, R., & Matzner, W. T. (2004). The neurobiology of trust. *Ann N Y Acad Sci, 1032*, 224-227. doi: 10.1196/annals.1314.025

Zaki, J., Weber, J., Bolger, N., & Ochsner, K. (2009). The neural bases of empathic accuracy. *Proc Natl Acad Sci U S A, 106*(27), 11382-11387. doi: 10.1073/pnas.0902666106

Zhou, Z., Zhu, G., Hariri, A. R., Enoch, M. A., Scott, D., Sinha, R., . . . Goldman, D. (2008). Genetic variation in human NPY expression affects stress response and emotion. *Nature, 452*(7190), 997-1001. doi: 10.1038/nature06858

Zubieta, J. K., Heitzeg, M. M., Smith, Y. R., Bueller, J. A., Xu, K., Xu, Y., . . . Goldman, D. (2003). COMT val158met genotype affects mu-opioid neurotransmitter responses to a pain stressor. *Science, 299*(5610), 1240-1243. doi: 10.1126/science.1078546
