# Supplementary material for: Association of Short-term Change in Leukocyte Telomere Length With Cortical Thickness and Outcomes of Mental Training Among Healthy Adults: A Randomized Clinical Trial
Source: JAMA Netw Open. 2019 Sep 25;2(9):e199687. doi: 10.1001/jamanetworkopen.2019.9687 (PMC6763984; doi:10.1001/jamanetworkopen.2019.9687)
Supplement: Supplement 2. — eAppendix 1. Detailed Methodology eAppendix 2. Descriptive Analyses eAppendix 3. Random Slopes Model eTable 1. Correlations Between Change in LTL and Leukocytic Cell Type Concentrations in the RCC eAppendix 4. Exploratory Analyses eAppendix 5. Residual Checks eFigure 1. Change in Telomere Length and Cortical Thickness From Baseline to 9-Month Follow-up eFigure 2. Relative Difference in Telomere Length per Training Module eFigure 3. DLTL and ∆CT in Right Precuneus/PCC ROI eTable 2. Mean (SD) of Baseline Personality Trait Scores eTable 3. Results of Exploratory Analyses eTable 4. Mean (SD) Unmodelled Change in LTL After Each Training Module eTable 5. Available Samples and Reasons for Missing Values in the Raw Data eTable 6. Available Samples and Reasons for Missing Values in Change Scores eReferences [file jamanetwopen-2-e199687-s002.pdf]

**Supplementary Online Content**

Puhlmann LMC, Valk SL, Engert V, et al. Association of short-term change in leukocyte telomere length with cortical thickness and outcomes of mental training among healthy adults: a randomized clinical trial. *JAMA Netw Open*. 2019;2(9):e199687. doi:10.1001/jamanetworkopen.2019.9687


**eAppendix 1.** Detailed Methodology

**eAppendix 2.** Descriptive Analyses

**eAppendix 3.** Random Slopes Model

**eTable 1.** Correlations Between Change in LTL and Leukocytic Cell Type Concentrations in the RCC

**eAppendix 4.** Exploratory Analyses

**eAppendix 5.** Residual Checks

**eFigure 1.** Change in Telomere Length and Cortical Thickness From Baseline to 9-Month Follow-up

**eFigure 2.** Relative Difference in Telomere Length per Training Module

**eFigure 3.** *D*LTL and ΔCT in Right Precuneus/PCC ROI

**eTable 2.** Mean (SD) of Baseline Personality Trait Scores

**eTable 3.** Results of Exploratory Analyses

**eTable 4.** Mean (SD) Unmodelled Change in LTL After Each Training Module

**eTable 5.** Available Samples and Reasons for Missing Values in the Raw Data

**eTable 6.** Available Samples and Reasons for Missing Values in Change Scores

**eReferences**


This supplementary material has been provided by the authors to give readers additional information about their work.

© 2019 Puhlmann LMC et al. *JAMA Network Open.*

**eAppendix 1. Detailed Methodology**

*LMM for the analysis of brain structure.*

We assessed the relation between *D*LTL and cortical thickness change (ΔCT) in a whole-brain linear mixed model (LMM) with the following terms:

$\Delta CT_{ij} = ß_0 + ß_1 * age_i + ß_2 * BMI_i + ß_3 * sex_i + ß_4 * \text{time-point}_j + \mathbf{ß_5 * \mathit{D}LTL_{ij}} + rand(subj)_i$

where $ß_0$ is the intercept, i = subject and j = time point of measurement. To account for subject-specific variance in total change rates we included a random intercept for each subject, rand(subj).

*LMM for the analysis of training effects.*

The impact of a term of interest, such as 'module', on *D*LTL was evaluated by comparing the fit of a full model (see below) with a reduced model lacking only the term of interest, by means of a likelihood ratio test.[1] Models were fitted using maximum likelihood (ML) estimation to accurately compare the best fitting model estimates. The full model included the following terms:

$DLTL_{ij} = ß_0 + ß_1 * age_i + ß_2 * BMI_i + ß_3 * sex_i + ß_4 * \text{time-point}_j + \mathbf{ß_{5-7} * module_{ij}} +$

$\mathbf{ß_{8-10} * timepoint_j \times module_{ij}} + rand(subj)_i$ ,

where $ß_0$ is the the intercept, i = subject and j = time point of measurement. We included a random intercept for each subject, rand(subj), to account for subject-specific variance in total change rates.

Two main model comparisons were conducted to assess potential training effects. In the first comparison, the above full model ('training model 1') was compared to a model lacking the timepoint by module interaction term ('training module 2'). This test examined whether the effect of the modules differed depending on the timepoint at which they were practiced. Since the three training cohorts completed most modules at different timepoints, a significant module by time interaction could also be evidence that the effect of module differed depending on which training cohort practiced the module. In the second model comparison, training module 2 was contrasted with a model lacking both the timepoint by module interaction term and the simple term module. This test examined whether there were any differences in the average effects of the modules, including no training.

N = 699 observations from 298 unique participants, who provided data for all relevant covariates, were included in the analysis of training intervention effects (up to three measures from the same participants).

© 2019 Puhlmann LMC et al. *JAMA Network Open.*

*LMMs for follow-up analyses.*

We conducted several unplanned analyses to further clarify the results of our whole brain analysis. These follow-up analyses were conducted as cluster level tests, i.e. only analyzed ΔCT of the vertices in the region that was significant in the main analysis. We first aimed to assess if measured telomere shortening and lengthening were both associated with ΔCT. For this purpose, we split the sample into observations of shortening and lengthening. We then analyzed the relation to cluster-level ΔCT by means of two separate LMMs, one for shortening and one for lengthening. The variables of these LMMs were the same as for the main analysis (see *LMM for the analysis of brain structure*).

Secondly, we wanted to assess whether the observed association between *D*LTL and ΔCT was reliable across the nine months. To this end, we analyzed the association again, this time through three separate linear models, one for each individual change interval (T0 to T1, T1 to T2 and T2 to T3). For these analyses each subject had only one data point, which is why we used linear models rather than linear mixed models. The variables of the linear models were the same as for the main LMM, except the variable "time-point" was redundant here and therefore not included, and there was no random intercept per subject.

Finally, we conducted an exploratory follow-up analysis to the null effect of training modules. In this analysis, we tested whether a training effect could be detected when examining telomere length change from baseline to the nine-month follow-up, combining the entire training period of TC1 and TC2, compared to no training in the RCC. Even though no significant effects of modules were detected in our main analyses, training modules may still have had incremental effects each, which could sum up to a significant effect. For example, stress reduction is one of the mechanisms that may affect LTL, and all three training modules had the potential to reduce stress, as outlined in the introduction. Thus, comparing change after the entire nine months is a powerful way to test whether the sum of all training periods had an effect on telomere length compared to no training.

To this end, we recalculated *D*LTL and ΔCT from the baseline and nine months follow-up scores. For this analysis each subject had only one data point, which is why we used a linear model rather than linear mixed model. The full model included a random intercept, terms for age, BMI and sex, as well as a binary variable coding the 'training' or 'no training' groups. The reduced model lacked only this binary variable. Since the models did not include any random terms, they could be compared using an F-statistic rather than a $\chi^2$ likelihood ratio test..

**eAppendix 2. Descriptive analyses.**

Baseline (T0) LTL correlated negatively with age ($r$ = -0.37, $p$ <.001) and BMI ($r$ = -0.13, $p$ = 0.021). A student $t$-test showed no significant sex differences in baseline LTL ($t$(303) = 1.01, $p$ = .31). The LTL difference scores corrected for regression to the mean (*D*LTL) are here expressed so that a negative value indicates attrition and a positive value gain. They were highly correlated with simple LTL difference scores ($r$ = 0.948, $p$ < .001). All of the reported results show the same pattern if calculated with simple difference scores instead of *D*LTL.

© 2019 Puhlmann LMC et al. *JAMA Network Open.*

**eAppendix 3. Random slopes model.**

The strength of the relation between change in telomere length and cortical thickness likely differs between participants. For participants whose data follow a pattern close to the overall average, the model will provide a better fit than for others. This can lead to systematic under- or over-estimations of change for particular subjects, and consequently to dependence between subject ID and model residuals. Since subjects of the RCC provided repeated measures of LTL under the same conditions, it is possible to estimate and control for this subject-level variance through random slopes. To this end, we analyzed the association between *D*LTL and ΔCT again with an additional random slopes term for subjects' LTL added to the original linear mixed model. For this follow-up analysis, we conducted a cluster level test, i.e. only analyzed ΔCT of the vertices in the region that was significant in the main analysis. The results showed that even when including random slopes, change in telomere length was significantly associated with cortical thickness change (mean $t(161) = 3.77$; $p < .001$, $r = .285$).

*Blood count change*

The LTL assay of the current study included peripheral blood mononuclear cells [PBMC] (monocytes and lymphocytes), as well as granulocytes (neutrophils, basophils, and eosinophils). Measures of leukocyte telomere length (LTL) can be influenced by the composition of these leukocytic cell types in a given blood sample, since some cell types possess shorter telomeres than others.[2,3] Covarying the change in concentrations of white blood cell types in statistical analyses can correct for their influence on measured LTL change, achieving a better estimate of the actual change in length that is independent of changes in cell distributions. To this end, we first assessed the relation between change in LTL and change in each of the five assayed types of white blood cells through correlational analyses. Changes in leukocytic cell types were partially correlated, but no change in cell type concentration was significantly related to *D*LTL (see eTable 1), which suggests that the observed *D*LTL was not driven by a redistribution of cell types. To control for any potential influences within the our analysis of interest, we nonetheless analyzed the association between *D*LTL and ΔCT again in the cluster identified in the main analysis, with terms for change in each cell type concentration added to the original linear mixed model.

The results showed that even when controlling for redistribution of leukocytic cell types, *D*LTL was significantly associated with ΔCT (mean $t(141) = 3.78$; $p < .001$; $r = 0.304$).

© 2019 Puhlmann LMC et al. *JAMA Network Open.*

**eTable 1. Correlations Between Change in LTL and Leukocytic Cell Type Concentrations in the RCC.**

| | Δlymph. | Δmonoc. | Δbasoph. | Δeosinoph. | Δneutroph. |
|---|---|---|---|---|---|
| ***D*LTL** | $r$ = .096<br>$p$ = .236 | $r$ = .049<br>$p$ = .550 | $r$ = .132<br>$p$ = .103 | $r$ = -.054<br>$p$ = .505 | $r$ = -.067<br>$p$ = .408 |
| **Δlymphocytes** | 1 | $r$ = .237<br>$p$ = .003** | $r$ = .186<br>$p$ = .022* | $r$ = .127<br>$p$ = .117 | $r$ = -.891<br>$p$ <.001** |
| **Δmonocytes** | - | 1 | $r$ = .016<br>$p$ = .845 | $r$ = .055<br>$p$ = .504 | $r$ = -.455<br>$p$ < .001** |
| **Δbasophiles** | - | - | 1 | $r$ = .070<br>$p$ = .394 | $r$ = -.209<br>$p$ = .010* |
| **Δeosinophils** | - | - | - | 1 | $r$ = -.473<br>$p$ < .001** |
| **Δneutrophils** | - | - | - | - | 1 |

*: significant with $p$<.05; **: significant with $p$<.005; all $p$-values are uncorrected. "Δ" indicates simple difference scores; *D*LTL, leukocyte telomere length change corrected for regression to the mean.

*Smoking sensitivity analysis.*

Smoking has been associated with reduced cortical thickness and grey matter volume.[4,5] Similarly, robust cross-sectional associations between cigarette smoking and shorter telomere length have been interpreted as evidence that smoking increases telomere attrition[6,7]; although there is recent evidence from a convincing meta-analysis that the detected cross-sectional associations are unlikely to represent a causal association between smoking and telomere attrition.[8] Nonetheless, it may be argued that smoking can account for the association between LTL shortening and cortical thinning identified here. Smoking was an exclusion criterion in the current study, but since this behavior could not be monitored or actively controlled, we encouraged honesty in reporting any smoking behavior after study commencement. Six participants of the RCC subsample with MRI data reported that they smoked five or more cigarettes a week. To account for this, we performed a smoking sensitivity analysis, in which our main model was recalculated without the six smokers (losing a total of 14 cortical thickness change measures). The results did not vary from those found when including the smokers (mean $t(147) = 3.68$; $p$ <.001; $r$ = .291).

© 2019 Puhlmann LMC et al. *JAMA Network Open.*

**eAppendix 4. Exploratory Analyses**

Our main analysis of CT revealed an association of *D*LTL specifically with ΔCT in the left precuneus/PCC. In a subsequent analysis we explored whether a similar but weaker association may nonetheless be detected with ΔCT in the right precuneus/PCC when using a ROI based approach. To this end, we extracted the coordinates of the vertex with the highest association to *D*LTL in the left precuneus/PCC and identified the corresponding vertex on the right hemisphere. We then created a 10mm Gaussian smooth around this vertex and averaged ΔCT in this region. The resulting measure of ΔCT correlated significantly positively with *D*LTL ($r = 0.205$, $p = 0.008$, eFigure 3). This outcome indicates that the observed association between *D*LTL and precuneus/PCC structure, while stronger in the left hemisphere, may be contralaterally consistent.

Furthermore, in addition to our main analysis of the effects of the training intervention, we conducted several exploratory analyses that addressed specific conditions under which an effect of training may be identified.

Firstly, we re-assessed potential training effects of the three modules in a within-subject analysis. This analysis was designed to be particularly sensitive to differential effects of the training modules and may thus identify differences despite the non-significant main analysis. We applied within-subject centering to emphasize potential relative differences in the effects of the training modules for each participant. Further, we focused only on differences within subjects from the TC1 and TC2, who completed all three training modules. Accordingly, and in contrast to our main analysis, all model comparison were here conducted on the same set of participants, resulting in a cleaner comparison of within-subject differences. Potential sequence or carry-over effects of the training modules were, however, not controlled for. Significance was tested following the same steps as described above (see *LMM for the analysis of training effects)*. The full model had the same terms as the 'training model 2', and the reduced model lacked the term 'module'. Likelihood ratio tests identified no effect of module, $\chi^2_1 = 1.72$, p = 0.19 (eFigure 2).

We further explored whether the effect of training was moderated through practice frequency or by participants' personality traits, since openness, neuroticism, and agreeableness were previously found to moderate training effects on LTL.[9] Even though no overall effect of training was observed, a significant interaction with a moderating variable could identify subgroups that did benefit of the training, particularly in light of the considerable inter-individual variation in LTL change (see Figure 3 in the paper). Neuroticism, agreeableness, and openness values were acquired at baseline from all study participants, using the NEO five factor inventory (NEO-FFI,[10,11]). Average neuroticism, agreeableness, and openness scores are shown in eTable 2. We assessed the potential moderating effects of each of these variables through

© 2019 Puhlmann LMC et al. *JAMA Network Open.*

five additional linear mixed models. Each model was made up of the same terms as the model for our main analysis (see *LMM for the analysis of training effects)*, plus one additional term for a personality trait or practice frequency, which was also interacted with the term module. The fit of these full models was then compared with a reduced model that lacked the term of interest, by means of a likelihood ratio test.[1] Models were fitted using maximum likelihood (ML). P-values of these exploratory analyses were corrected for multiple testing using the Bonferroni method within each conceptual framework (personality traits and practice frequency, respectively).

LMM comparisons showed that practice frequency of dyads or meditation core exercises did not moderate the effect of training modules. Similarly, participants' baseline levels of neuroticism, agreeableness, or openness did not significantly moderate the effect of training when corrected for multiple comparisons (eTable 3).

**eAppendix 5. Residual Checks**

Residual checks were conducted to examine whether the assumptions of linear mixed models were met. For the assessment of residual distributions from cortical thickness analyses conducted using SurfStat, we averaged residuals as well as predicted ΔCT values from each model across all vertices in the identified cluster. Visual inspection of residual plots revealed that all models' residuals displayed satisfactory approximation to normal distribution. Variance inflation factors of the models' main effects indicated uncritical levels of multicollinearity.[12] Estimates of cook's distances showed no evidence of highly influential observations (all cook's $d < 0.4$)[13]

© 2019 Puhlmann LMC et al. *JAMA Network Open.*

**eFigure 1. Change in Telomere Length and Cortical Thickness From Baseline to 9-Month Follow-up.**

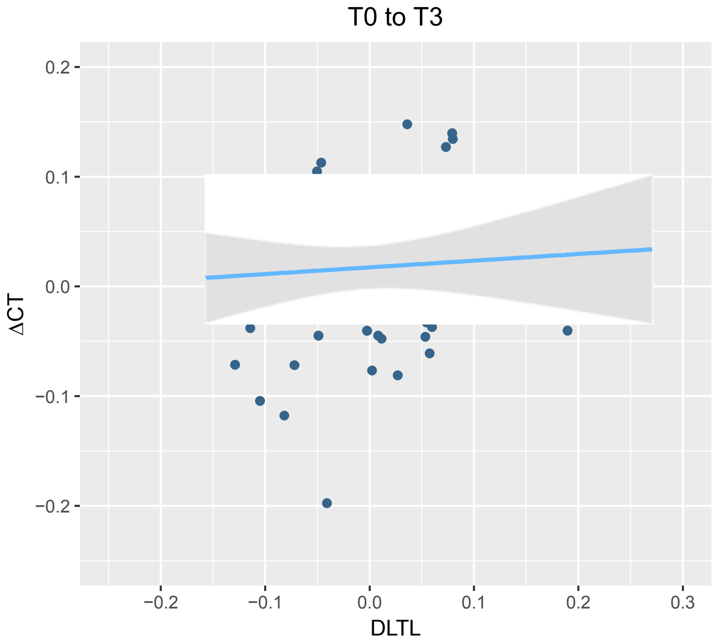


For visual display through scatter plots, ΔCT in the precuneus/PCC region was averaged and plotted against *D*LTL. The displayed regression line was derived from a linear model controlling for age, BMI and sex. Shaded areas represent 95% confidence intervals. *D*LTL, leukocyte telomere length change; ΔCT, cortical thickness change.

© 2019 Puhlmann LMC et al. *JAMA Network Open.*

**eFigure 2. Relative Difference in Telomere Length per Training Module.**

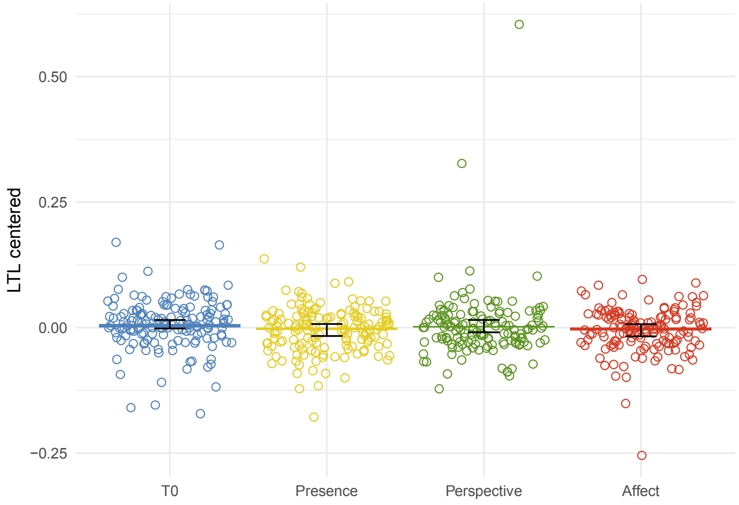


Bar plots show estimated mean relative difference in telomere length per module. Estimates were derived from the linear mixed model analysis of within-subject effects on within-subject mean centered scores, with all covariates held constant at their mean. Each circle represents a within-subject mean centered score. Error bars represent 95% confidence intervals. T0 represents the telomere length at study baseline. LTL, leukocyte telomere length.

© 2019 Puhlmann LMC et al. *JAMA Network Open.*

**eFigure 3. *D*LTL and ΔCT in right precuneus/PCC ROI**

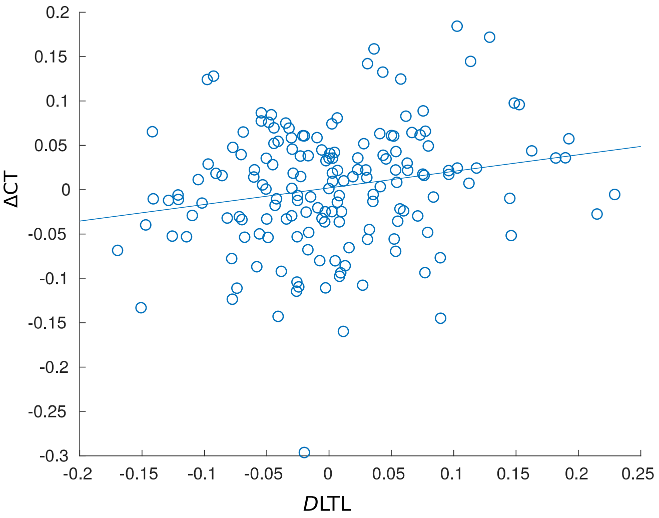


Correlation between *D*LTL and ΔCT in the right precuneus/PCC region around the vertex with the coordinates [10, -60, 10].

© 2019 Puhlmann LMC et al. *JAMA Network Open.*

**eTable 2. Mean (SD) of Baseline Personality Trait Scores.**

| | Total | No training | Presence | Affect | Perspective |
|---|---|---|---|---|---|
| **Neuroticism** (BL) | 17.8 (7.3) | 18.8 (7.0) | 18.4 (7.2) | 18.0 (7.1) | 18.3 (7.2) |
| **Openness** (BL) | 27.6 (4.5) | 27.3 (5.1) | 27.6 (4.4) | 27.7 (4.3) | 27.4 (4.3) |
| **Agreeableness** (BL) | 32.3 (3.6) | 31.8 (3.8) | 32.1 (3.5) | 32.4 (3.4) | 32.0 (3.4) |

All values in the above table represent baseline personality measures. The columns No training, Presence, Affect and Perspective describe the sample characteristics of each training group in terms of baseline personality scores. BL, baseline; SD, standard deviation.

© 2019 Puhlmann LMC et al. *JAMA Network Open.*

**eTable 3. Results of Exploratory Analyses**

| | **sample N** | $\chi^2$ | **df** | **p-value** (uncorr.) | **p-value** (corr.) |
|---|---|---|---|---|---|
| **Neuroticism** (BL)[a] | 690 | 1.64 | 3 | 0.651 | >.9 |
| **Agreeableness** (BL)[a] | 690 | 7.80 | 3 | 0.050[(*)] | 0.150 |
| **Openness** (BL)[a] | 690 | 0.19 | 3 | 0.979 | >.9 |
| **Meditation practice frq.**[b] | 490 | 0.821 | 3 | 0.844 | >.9 |
| **Dyad practice frq.**[b] | 348 | 0.242 | 2 | 0.886 | >.9 |

To control for the inflated likelihood of type II errors due to multiple testing, p-values of these secondary analyses were corrected for each additional analysis within the same conceptual framework (personality traits and practice frequency) using the Bonferroni method. BL, Baseline; SD, standard deviation.

[a]: compared to a model only including the baseline variable without interaction

[b]: compared to a model not including practice time (a main effect of practice time would suggest that practice influenced the efficacy of training. An interaction term with module was included to test whether the influence of practice time differed for the distinct training modules)

**eTable 4. Mean (SD) unmodelled change in LTL after each training module**

| | T0 to T1 | T1 to T2 | T2 to T3 |
|---|---|---|---|
| **No Training** | 0.0035 (0.083) | -0.0019 (0.072) | -0.0021 (0.085) |
| **Presence** | -0.0110 (0.072) | | |
| **Affect** | -0.0108 (0.083) | 0.0074 (0.067) | -0.0101 (0.063) |
| **Perspective** | | -0.0014 (0.066) | -0.0059 (0.069) |

Change in LTL after each type of training module (Affect, Presence and Perspective) or training-free interval (No Training). Telomere length is reported as uncorrected change in T/S ratio, the relative ratio of telomere repeat copy number to single-copy gene. LTL, leukocyte telomere length; SD, standard deviation.

© 2019 Puhlmann LMC et al. *JAMA Network Open.*

**eTable 5. Available Samples and Reasons for Missing Values in the Raw Data**

| Variable | Assess-ment TPs | Available N (raw data) | Reasons for missingness |
|---|---|---|---|
| **Age** | T0 | 332 | N/A |
| **BMI** | T0 | 317 | Study dropout (N = 2) |
| | | | Study exclusion (N = 2) |
| | | | No BMI assessed (N = 11) |
| **Sex** | T0 | 332 | N/A |
| **NEO-questionaire** (BL) | T0 | 323 | Study dropout (N = 2) |
| | | | Study exclusion (N = 2) |
| | | | No Questionnaire (N = 5) |
| **LTL** (T/S ratio) | T0-T3 | **T0** N = 314 | Study dropout (N = 2) |
| | | | Study exclusion (N = 2) |
| | | | No blood sampling (exclusion) (N = 3) |
| | | | No blood sampling (missing) (N = 11) |
| | | **T1** N = 297 | Study dropout (N = 11) |
| | | | No blood sampling (missing) (N = 17) |
| | | **T2** N = 213 | Study dropout (N = 6) |
| | | | TC3 Study completed (N = 77*) |
| | | | No blood sampling (missing) (N = 18) |
| | | **T3** N = 217 | Study dropout (N = 7) |
| | | | No blood sampling (missing) (N = 7) |
| | | | No blood sampling (missing) (N = 16) |
| **MRI** (RCC) | T0-T3 | **T0** N = 81 | MRI dropout (N = 2) |
| | | | MRI exclusion [medical] (N = 5) |
| | | | Quality control (N = 2) |
| | | **T1** N = 75 | MRI dropout (N = 4) |
| | | | MRI exclusion [medical] (N = 1) |
| | | | No MRI (missing) (N = 2) |
| | | | Quality control (N = 1) |
| | | **T2** N = 70 | MRI dropout (N = 3) |
| | | | MRI exclusion [medical] (N = 2) |
| | | | No MRI (missing) (N = 1) |
| | | | Quality control (N = 2) |
| | | **T3** N = 72 | No MRI (missing) (N = 1) |

Study dropouts continue to count towards missing data at the subsequent measurement points. MRI specific dropouts are unrelated from general study dropouts. BL, Baseline; TP, time-point; LTL, leukocyte telomere length; RCC, retest control cohort.

© 2019 Puhlmann LMC et al. *JAMA Network Open.*

**eTable 6. Available Samples and Reasons for Missing Values in Change Scores**

| | Available N (per TP) | Reasons for missingness |
|---|---|---|
| **LTL** (T/S ratio) | **T0 to T1** N = 287 | Study dropout, T0 & T1 (N = 13) |
| | | Study exclusion (N = 2) |
| | | No blood sampling (exclusion) (N = 3) |
| | | No blood sampling at T0 or T1 (N = 27) |
| | **T1 to T2** N = 209 | Study dropout T2 (N = 6) |
| | | TC3 Study completed (N = 77*) |
| | | No blood sampling at T1 or T2 (N = 22) |
| | **T2 to T3** N = 205 | Study dropout T3 (N = 7) |
| | | No blood sampling at T2 or T3 (N = 19) |
| **Cortical thickness** (RCC) | **T0 to T1** N = 74 | MRI dropout T0 & T1 (N = 6) |
| | | MRI exclusion [medical] T0 & T1 (N = 6) |
| | | MRI missing T1 (N = 2) |
| | | Quality control T0 (N = 1), T1 (N = 1) |
| | **T1 to T2** N = 68 | MRI dropout T2 (N = 3) |
| | | MRI exclusion [medical] T2 (N = 2) |
| | | No MRI T2 (N = 1) |
| | | Quality control T1&T2 (N = 1), T2 (N = 1) |
| | **T2 to T3** N = 69 | No MRI T3 (N = 1) |

Study dropouts continue to count towards missing data at the subsequent measurement points. MRI specific dropouts are unrelated from general study dropouts. Total Ns are based on the available raw values and may not match Ns included in linear mixed models due to missing values in the covariates. LTL, Leukocyte telomere length; RCC, retest control cohort; TP, time-point.

© 2019 Puhlmann LMC et al. *JAMA Network Open.*

**eReferences**


1. Dobson AJ. *An Introduction to Generalized Linear Models.* Boca Raton, Florida: Chapman & Hall/CRC; 2002.
2. Epel E. How “reversible” is telomeric aging? *Cancer Prev Res*. 2012;5(10):1163-1168. doi:10.1158/1940-6207.CAPR-12-0370.
3. Aviv A, Valdes AM, Spector TD. Human telomere biology: Pitfalls of moving from the laboratory to epidemiology. *Int J Epidemiol*. 2006;35(6):1424-1429. doi:10.1093/ije/dyl169.
4. Kühn S, Schubert F, Gallinat J. Reduced thickness of medial orbitofrontal cortex in smokers. *Biol Psychiatry*. 2010;68(11):1061-1065. doi:10.1016/j.biopsych.2010.08.004.
5. Gallinat J, Meisenzahl E, Jacobsen LK, et al. Smoking and structural brain deficits: A volumetric MR investigation. *Eur J Neurosci*. 2006;24(6):1744-1750. doi:10.1111/j.1460-9568.2006.05050.x.
6. Valdes AM, Andrew T, Gardner JP, et al. Obesity, cigarette smoking, and telomere length in women. *Lancet*. 2005;366(9486):662-664. doi:10.1016/S0140-6736(05)66630-5.
7. Astuti Y, Wardhana A, Watkins J, Wulaningsih W. Cigarette smoking and telomere length: A systematic review of 84 studies and meta-analysis. *Environ Res*. 2017;158(March):480-489. doi:10.1016/j.envres.2017.06.038.
8. Bateson M, Aviv A, Bendix L, et al. Smoking does not accelerate leucocyte telomere attrition: a meta-analysis of 18 longitudinal cohorts. *R Soc Open Sci*. 2019;6(190420).
9. Conklin QA, King BG, Zanesco AP, et al. Insight meditation and telomere biology: The effects of intensive retreat and the moderating role of personality. *Brain Behav Immun*. 2018. doi:10.1016/j.bbi.2018.03.003.
10. Borkenau P, Ostendorf F. *NEO-Fünf-Faktoren-Inventar (NEO-FFI) Nach Costa Und McCrae, Revidierte Auflage. [NEO Five-Factor Inventory (NEO-FFI) According to Costa & McCrae, Rev. Ed.]*. Göttingen: Hogrefe; 2008.
11. Borkenau P, Ostendorf F. *NEO-Fünf-Faktoren Inventar (NEO-FFI) Nach Costa Und McCrae: Handanweisung.* Göttingen: Hogrefe; 1993.
12. Hair JF, Black WC, Babin BJ, Anderson RE, Tatham RL. *Multivariate Data Analysis*. 5th ed. New York: Prentice Hall Upper Saddle River; 1998. doi:10.1007/978-3-319-01517-0_3.
13. Fox J. *Regression Diagnostics: An Introduction*. Vol 79. Sage; 1991.


© 2019 Puhlmann LMC et al. *JAMA Network Open.*
