# Supplementary material for: Association of Short-term Change in Leukocyte Telomere Length With Cortical Thickness and Outcomes of Mental Training Among Healthy Adults: A Randomized Clinical Trial
Source: JAMA Netw Open. 2019 Sep 25;2(9):e199687. doi: 10.1001/jamanetworkopen.2019.9687 (PMC6763984; doi:10.1001/jamanetworkopen.2019.9687)
Supplement: Supplement 3. — Data Sharing Statement [file jamanetwopen-2-e199687-s003.pdf]

Data Sharing Statement

Puhlmann. Association of Short-term Change in Leukocyte Telomere Length With Cortical Thickness and Outcomes of Mental Training Among Healthy Adults. *JAMA Netw Open*. Published September 25, 2019. 10.1001/jamanetworkopen.2019.9687

Data
Data available: No

Additional Information
Explanation for why data not available: In line with new data regulations (General Data Protection Regulation, GDPR), we regret that data cannot be shared publicly because we did not obtain explicit participant agreement for data-sharing with third parties. Our work is based on personal data (age, sex and medical data) that could be matched to individuals. The data is therefore pseudonominized rather than anonymized and falls under the GDPR. Data are available upon request (contact via puhlmann@cbs.mpg.de).
